# A global systematic scoping review of studies analysing indicators, development, and content of national-level physical activity and sedentary behaviour policies

**DOI:** 10.1186/s12966-018-0742-9

**Published:** 2018-11-28

**Authors:** Bojana Klepac Pogrmilovic, Grant O’Sullivan, Karen Milton, Stuart J. H. Biddle, Adrian Bauman, Fiona Bull, Sonja Kahlmeier, Michael Pratt, Zeljko Pedisic

**Affiliations:** 1 Institute for Health and Sport, Victoria University, Ballarat Road, Footscray, Melbourne, VIC 3001 Australia; 2 Norwich Medical School, University of East Anglia, Norwich Research Park, Norwich, Norfolk, NR4 7TJ UK; 3 Institute for Resilient Regions, University of Southern Queensland, 37 Sinnathamby Boulevard, Springfield Central, QLD 4300 Australia; 4 Sydney School of Public Health, University of Sydney, Camperdown, Sydney, NSW Australia; 5 Surveillance and Population Based Prevention, Prevention of Noncommunicable Disease, World Health Organization, Geneva 27, Switzerland; 6 Faculty of Human Science, The University of Western Australia, Perth, Australia; 7 Epidemiology, Biostatistics, and Prevention Institute, University of Zürich, Hirschengraben 84, 8001 Zürich, Switzerland; 8 San Diego School of Medicine, University of California, 9500 Gilman Drive, San Diego, USA

**Keywords:** Physical activity, National policy, Sedentary behaviour, Sitting, Physical inactivity, National plan, Strategy

## Abstract

**Background:**

National policy approaches to physical activity (PA) promotion and sedentary behaviour (SB) reduction are needed to address rising rates of non-communicable diseases. Understanding the policy process and impact through robust research and evaluation is crucial for facilitating successful reforms in national health policy. This scoping review, therefore, aimed to map the evidence on indicators, development, and content of national PA and/or SB policies globally.

**Methods:**

A systematic search of academic and grey literature was conducted through six bibliographic databases, Google, and websites of three large organisations for PA promotion.

**Results:**

Out of 24,872 screened documents, 203 publications from 163 studies were selected. The selected studies investigated PA/SB policies in 168 countries worldwide, and we provided summary results for each of the countries. Overall, 69, 29, and 2% of the analyses of national PA/SB policies were conducted for high-, middle-, and low-income countries, respectively. Twenty-two percent of the studies mentioned SB policies as part of their analysis, with only one study focusing solely on assessing SB policies. Operational definitions of policy were found in only 13% of publications. Only 15% of the studies used a conceptual or theoretical framework. A large variety of methods were used for data collection and analysis of PA/SB policy.

**Conclusions:**

We found that PA policy research is much more developed than it was considered several years ago. Research around SB policies is still in its infancy, but it seems to have experienced some positive progress in the last few years. Three key issues were identified that should be addressed in further research: [i] there is a lack of PA/SB policy research in low- and middle-income countries, which is an important limitation of the current body of evidence; [ii] the definition of policy varied significantly across studies, and most studies did not rely on any theoretical framework, which may impede cross-study comparisons; and [iii] studies have used a variety of methods to analyse policy, which may also cause problems with comparability. Future PA/SB policy research should aim towards a clearer conceptualisation of policy, greater reliance on existing theoretical frameworks, and the use and further development of standardised methods for PA/SB policy analysis.

**Electronic supplementary material:**

The online version of this article (10.1186/s12966-018-0742-9) contains supplementary material, which is available to authorized users.

## Background

More than 40 million people a year die from noncommunicable diseases (NCDs), of which 15 million deaths are considered premature [1]. This accounts for around 70% of overall global mortality [2], with high rates in low-, middle-, and high-income countries [1]. Insufficient physical activity (PA) and sedentary behaviour (SB) are among the key risk factors for NCDs. Global estimates indicate that the latter was responsible for 3.8% of deaths from 2002 to 2011 [3] and the former for 9% of deaths in 2008 [4]. In 2013, the estimated cost of insufficient PA to worldwide health-care systems was around 53.8 billion international dollars [5]. Insufficient PA and prolonged sitting are, therefore, not just significant health risk factors for global mortality but also a vast economic burden for national health care systems. National policy approaches to PA promotion and SB reduction are an essential aspect needed to address rising rates of NCDs [6].

The policy environment is perceived as one of the important determinants influencing active living at the population level [7]. The main goals of public policy related to PA are to allow for creating supportive programs, infrastructure, and environments for people to engage in physically active lifestyles [8, 9]. Research related to PA has informed the development of policy in the health sector and non-health sectors such as education, transport, sport, and environment [10–12]. PA policy research has been developing since 1990s. This field of research lagged behind the research on health outcomes of PA by more than 30 years [13, 14]. Therefore, PA policy research is still widely considered to be an area in need of more research, particularly in terms of large-scale evaluations of implementation and impact [13, 15, 16].

Since 2000, two key global efforts have occurred in PA planning and policy [17]. In 2002, The World Health Organization (WHO) and the Centers for Disease Control and Prevention (CDC) in the United States of America (USA) conducted international consultations on PA policy development [18]. The consultations informed the development of the *Global Strategy on Diet, Physical Activity and Health,* which is perceived to be the first major global effort related to PA policy [19]. The strategy targeted governments, along with non-governmental agencies, as the main agents of social change that can enhance population PA levels by creating supportive environments. The second major initiative was the United Nations (UN) high-level meeting on NCDs in 2011, where physical inactivity was acknowledged as an important determinant of NCDs globally [17]. Along with these major global efforts, various international leadership and advocacy networks were established to support the promotion of PA, such as: *Red Actividad Fisica de las Americas*/Physical Activity Network of the Americas (RAFA/PANA) in 2000; Asia Pacific Physical Activity Network (AP-PAN) and the European Network for the Promotion of Health-Enhancing Physical Activity (HEPA Europe) in 2005; Global Advocacy for Physical Activity (GAPA) in 2007; Africa Physical Activity Network (AFRO-PAN) in 2010; Global Observatory for Physical Activity (GoPA!) in 2012; and Active Healthy Kids Global Alliance in 2014.

Studies on SB form a relatively new field of behavioural epidemiology. Interest in this area has started growing rapidly in the last decade, after epidemiological evidence indicated that long periods of sitting might pose a health risk, irrespective of one’s PA level [20]. It should be noted, however, that recent studies have questioned the validity of evidence on SB as an independent health risk factor [21–24]. The main goals of emerging SB related public policy is to allow for creating supportive programs, infrastructure, and environments to support people to minimise their time spent in SB and to break prolonged periods of SB. Although evidence on the prevalence, trends, determinants, and health outcomes of SB is emerging rapidly, the research around SB policies is scarce and still in its infancy. The Sedentary Behaviour Research Network was recently established as an international association for researchers and health professionals focusing specifically on SB, to support research in this area [25].

The development of the Global Strategy on Diet, Physical Activity and Health, along with several other global awareness-raising initiatives from the early 2000s, was viewed as a potential turning point after which more countries would establish national policies and strategies related to PA [26]. However, after a decade, the majority of countries had made limited progress on PA policy development [27, 28]. It has been suggested that further research is needed to provide new theoretical and practical insights to inform future PA and SB policy development [16, 17]. Understanding the policy process and impact through robust research and evaluation is crucial for facilitating successful reforms in national health policy [29] and to support all countries to prioritise and commit to increasing PA promotion [30].

A comparative scoping review from 2016 analysed three types of scientific evidence to inform physical activity policy [31] and a structured literature review and citation network analysis published in 2018 mapped the historical development of PA and health research [13]. However, the actual level of development of the PA/SB policy research has never been systematically evaluated. This systematic scoping review of academic and grey literature aimed to map the evidence on the indicators, development, and content of national PA and/or SB policies. We addressed the following four key questions: (i) Which countries and world regions have been covered by this type of research?; (ii) How is ‘policy’ conceptualised within the studies and to what extent were PA/SB policy studies based on conceptual/theoretical frameworks?; (iii) Which methods have been used for analysing PA/SB policies?; and (iv) What are the potential future directions of research in this area? This review will help inform national PA/SB policy development, public health promotion of physically active lifestyles, and future research on PA and/or SB policies.

## Methods

### Literature search

The primary search was performed through PubMed/MEDLINE, Scopus, Web of Science (including Science Citation Index Expanded - SCI-EXPANDED, Social Sciences Citation Index - SSCI, Arts & Humanities Citation Index - A&HCI, Conference Proceedings Citation Index- Science - CPCI-S, and Conference Proceedings Citation Index- Social Science & Humanities - CPCI-SSH), SPORTDiscus, Open Access Theses and Dissertations (OATD), and Networked Digital Library of Theses and Dissertations (NDLTD) databases using the entries *“physical inactivity”*, *“physical activity”*, *sitting*, and sedentar* in combination with the entries *policy* and *policies.* The full search syntaxes used for each database are available in Additional file 1. The search was performed through titles, abstracts and keywords of the articles. The secondary search was done through the references of all articles selected in the primary search and authors’ own archives. Additionally, for governmental reports and other non-academic documents, searches were conducted through Google and websites of the WHO and two major international PA promotion networks: the GoPA and the Active Healthy Kids Global Alliance.

### Inclusion criteria

To be included in the review publications had to meet the following criteria:

1. One of the aims of the publication was to analyse PA and/or SB policy or obesity, NCD prevention, sport for all/recreation, and/or other health-related policies that included an analysis of PA and/or SB;

2. The study analysed national-level policies. For federations and multi-state countries, only studies analysing the highest level governmental policies were taken into account (for example Australia and the USA). In the United Kingdom (UK), policy development can occur for all of the UK, as well as for individual home countries. Thus, policies were also included for Scotland, England, Wales, or Northern Ireland, for consistency with previous analyses of national PA/SB policies globally [11, 32–34].

3. The policy analysis was focused on the process of policy development and/or content of policy;

4. The full publication or at least its abstract was available in English.

We excluded publications that: evaluated impact of policy changes on levels of PA or SB; evaluated public opinion and/or knowledge about PA/SB policy/guidelines; analysed international, subnational (e.g. local, regional, territorial, provincial), or non-governmental PA/SB policies/guidelines; focused on policy implementation; or provided general, non-country specific policy recommendations.

### Definition of policy

In accordance with Colebatch [35] and Birkland [36], for the purpose of this study we defined public policy as ‘a broad orientation’, ‘an indication of normal practice’, ‘a specific commitment’, or ‘a statement of values’ [35] with the following attributes: (i) it is made by governments on the *“public’s”* behalf; (ii) it is structured as a response to a problem and orientated towards a desired state or a goal to solve the problem; and (iii) it is implemented and interpreted by private and public actors who have various understandings of solutions and problems [36]. It should be noted that this definition does not represent authors’ general view on how public policy should be defined. While some studies proposed more specific definitions of policy [11, 26, 37], we used this broad and inclusive definition simply because the aim of our scoping review was to capture all the various research related to PA and SB policy. In previous studies, national PA/SB guidelines were considered as a policy document [38] or a policy paper [39], an area of policy content [40] or an element of a successful policy approach [41, 42]. Some authors suggested, however, a clear distinction should be made between a *policy* (defined as a policy document) and PA and health *guidelines* or *recommendations* [11, 43, 44]. For the purpose of this review we considered national PA/SB guidelines as an indicator of government policy, because the act of issuing national PA/SB guidelines indicates that the government (as their issuing body) has policy supportive of promoting PA and reducing SB. To be as inclusive as possible, in the current study we, therefore, included studies analysing national-level PA/SB guidelines formally adopted and/or published by the government. We acknowledge, however, that there is no consensus among the researchers on this matter and that our definition of national PA/SB guidelines as an indicator of government policy may not necessarily be applicable in future PA/SB studies.

### Definition of policy analysis

No consensus has been achieved among researchers on what constitutes a policy analysis. Kustec Lipicer stated that synonyms for *policy evaluation* available in the literature are *analysis, appraisal, assessment, adjudgement, judgement, examination, critique, review, inspection, measuring* and *grading* of policy [45]. For the purpose of this study we considered the term *policy analysis* broadly and used it as a synonym for *evaluation*, *assessment*, and *review* of policy.

### Study selection and data extraction

The study selection was conducted in July 2017, independently by two authors, BKP and GO, whilst a third author, ZP, resolved discrepancies between the study selections. Extraction and tabulation of data was done by one author (BKP). Two authors (BKP and ZP) independently checked for inconsistencies in the extracted data and revised the tables (Additional files 2, 3, 4 and 5). From every included study, we extracted data on its scope (national or international), number of covered countries, focus of the study (including type of the analysed policy, country, and specific target population), the period from which policies were analysed, summary of methods used to analyse policies, and main national-level and international-level findings.

### Categorisation of countries

The World Bank’s list of 218 economies from June 2017 was used as the list of countries/states/regions/economies [46]. As mentioned above, we included four UK’s home nations separately, so the total number of countries encompassed in this review was 221. The authors are aware that some countries/states/regions/economies on the World Bank’s list cannot be termed as “countries” because of disputable political and legal issues. However, for the purpose of brevity, we used the term *country* as an abbreviation for “countries/states/regions/economies” on the World Bank’s list. The categorisation of the countries into four income groups: low income; lower middle income; upper middle income; and high income, as well as the division of countries into regions was also done using the World Bank’s list. According to the list *Europe and Central Asia* constitute one region. To enable drawing conclusions about geographically more specific areas, we additionally divided Europe into four regions as defined by the Publications Office of the European Union (EU) as part of EuroVoc.

## Results

### General findings

**Fig. 1:**
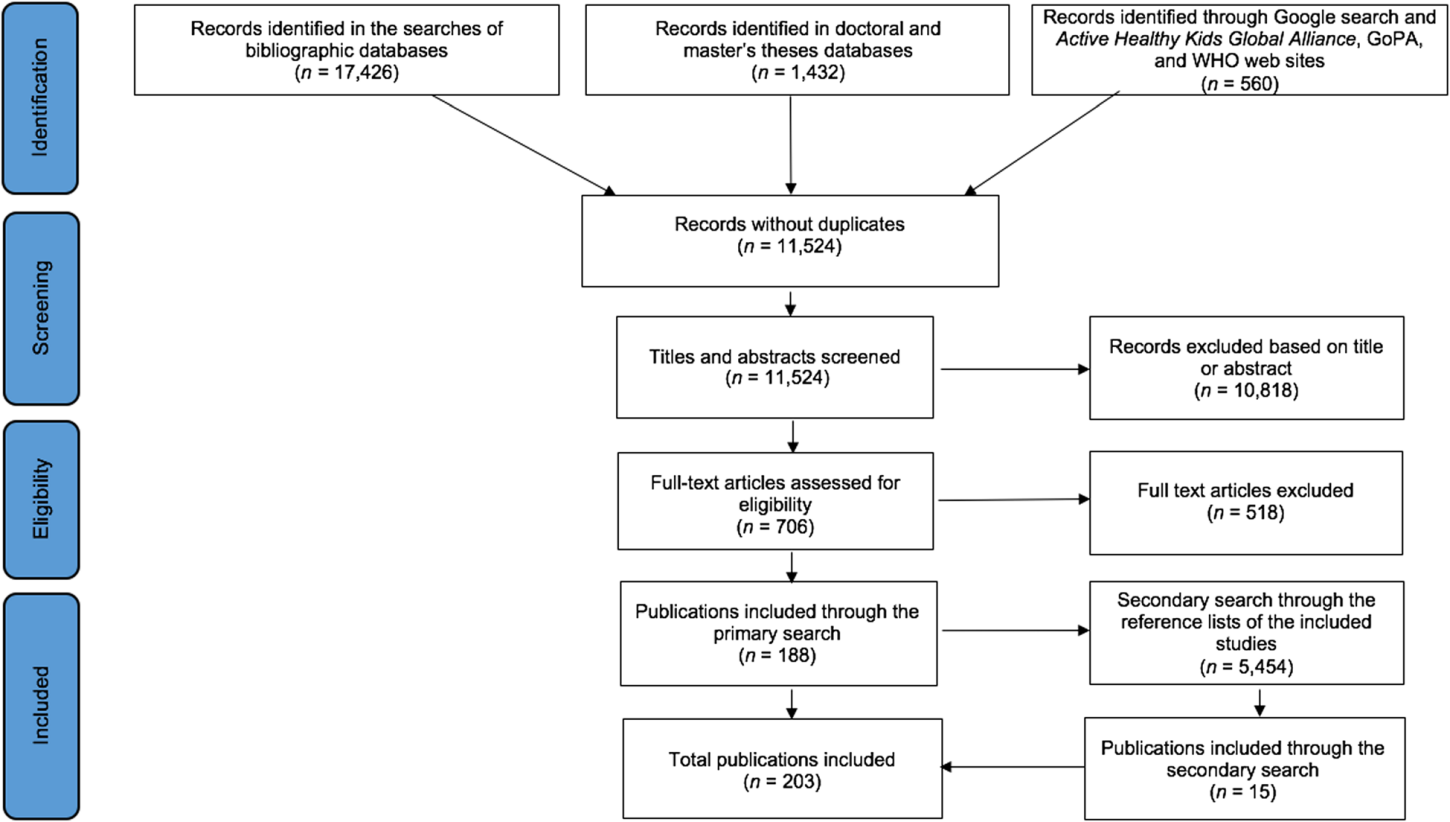
Flow diagram of the search and study selection process

In total, we screened 24,872 documents. Two hundred and three publications [6, 8, 10–12, 26, 32–34, 37–40, 43, 44, 47–234] from 163 original studies met the selection criteria (Fig. 1). A list of all studies with a short description, including the year of publication, key focus, study period, and methods, is presented in Additional file 2. We extracted data from each of the 163 studies (some of which included a single country and some of which included multiple countries) to create a breakdown of policy studies for each individual country. If a study included, for example, four countries, it is listed under each of these four countries separately in Additional file 3, creating 635 country-specific policy analyses in total. The full-texts of 12 academic publications were not in English but in Chinese (*n* = 2), Czech (*n* = 2), French (*n* = 2), Korean (*n* = 1), Portuguese (*n* = 2), and Spanish (*n* = 3). These publications were translated into English for data extraction purposes. The selected studies investigated PA and/or SB policies in 168 out of 221 countries worldwide. From these studies, seven were focused exclusively on PA/SB guidelines. The large majority of studies (72%) focused only on one country, whilst the remaining 28% of studies compared or presented an overview of two or more countries. The key findings of the included studies for each of the 168 countries separately are summarised in Additional file 3, whilst international (non-country specific) findings are presented in Additional file 4.

Active Healthy Kids Report Cards for Children and Youth or published articles based on the report card data comprised 40% of all the included studies. The Report Cards are developed under the Active Healthy Kids Global Alliance, a network of researchers, stakeholders, and health professionals [235]. This large international project is based on a Canadian initiative that now includes 38 countries [34]. Some countries like Canada, publish their Report Cards annually, but most other countries published them biennially. The Report Cards aim to assess how each country is performing in promoting and facilitating PA opportunities for children and youth [236]. The common nine indicators incorporated in most countries’ report cards are: (i) overall levels of PA; (ii) organized sport and PA; (iii) active play; (iv) active transportation; (v) SB; (vi) support from family and peers; (vii) school environment; (viii) community and the built environment; and (ix) government strategies and investments [237]. A group of experts from each country responsible for the development of the report card assesses performance against each of the indicators and provides usually alphabetical grades for each indicator (from A to F and INC as *incomplete*). The key findings from the last indicator, that is, *Government’s Strategies and Investments*, and the respective grade country experts assigned to their country are summarised in Additional file 3, whilst the joint findings and comparison of grades from the 2014 report cards [33] and the 2016 report cards [34] are summarised in Additional file 4.

A major contribution to worldwide PA policy monitoring was also provided by the GoPA [32, 176]. GoPA is a Council of the International Society of Physical Activity and Health (ISPAH), and was established to measure global progress in the area of PA research, surveillance, and policy [176]. GoPA collected data for 217 countries and confirmed data accuracy for 139 countries. For 53 countries, in our overall results, the only data included in the current review were from the GoPA country cards. GoPA developed PA country cards with six key indicators reported by key country informants: (i) general information on the country (including the Capital city, number of inhabitants, and life expectancy); (ii) PA prevalence among adults; (iii) health burden of insufficient PA (not meeting PA guidelines); (iv) existence of a national PA plan (yes/no); (v) information about PA surveillance (presence, year); and (vi) a research output metric based on bibliographical assessment of published peer reviewed journal articles on PA. The fourth indicator on the availability of a national or subnational PA plan was extracted for the purpose of this review. GoPA provided descriptive data on PA policy for 139 countries, which constitutes 22% of all findings identified in this review.

We found some discrepancies in findings, especially for those countries that were analysed by multiple independent studies (see Additional file 3). Some of the possible reasons include: authors’ subjectivity in assessment of the data; different methods used for analysing and obtaining the data; different interviewees involved in the study; and actual change in policy that occurred in the periods between studies.

### Findings by regions and economic standard

**Fig. 2:**
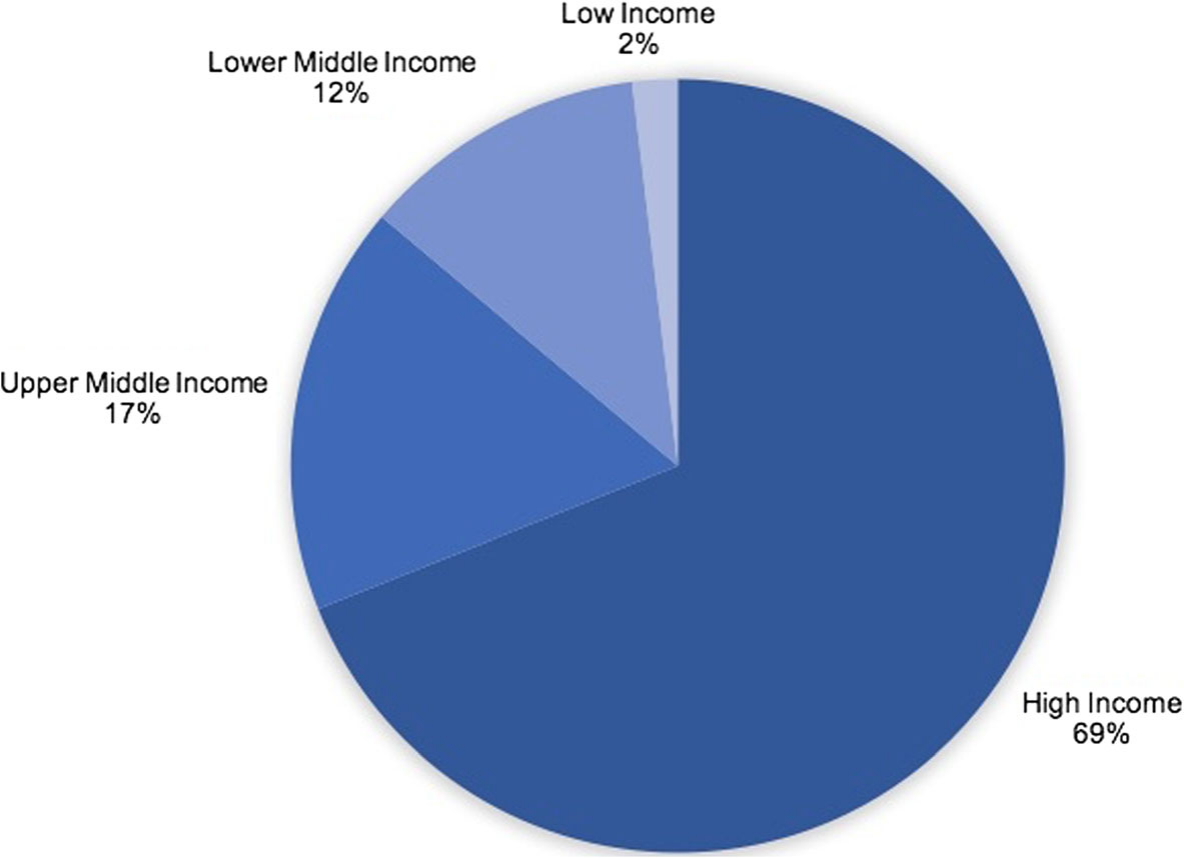
Distribution of PA/SB policy research across countries by economic standard

**Fig. 3:**
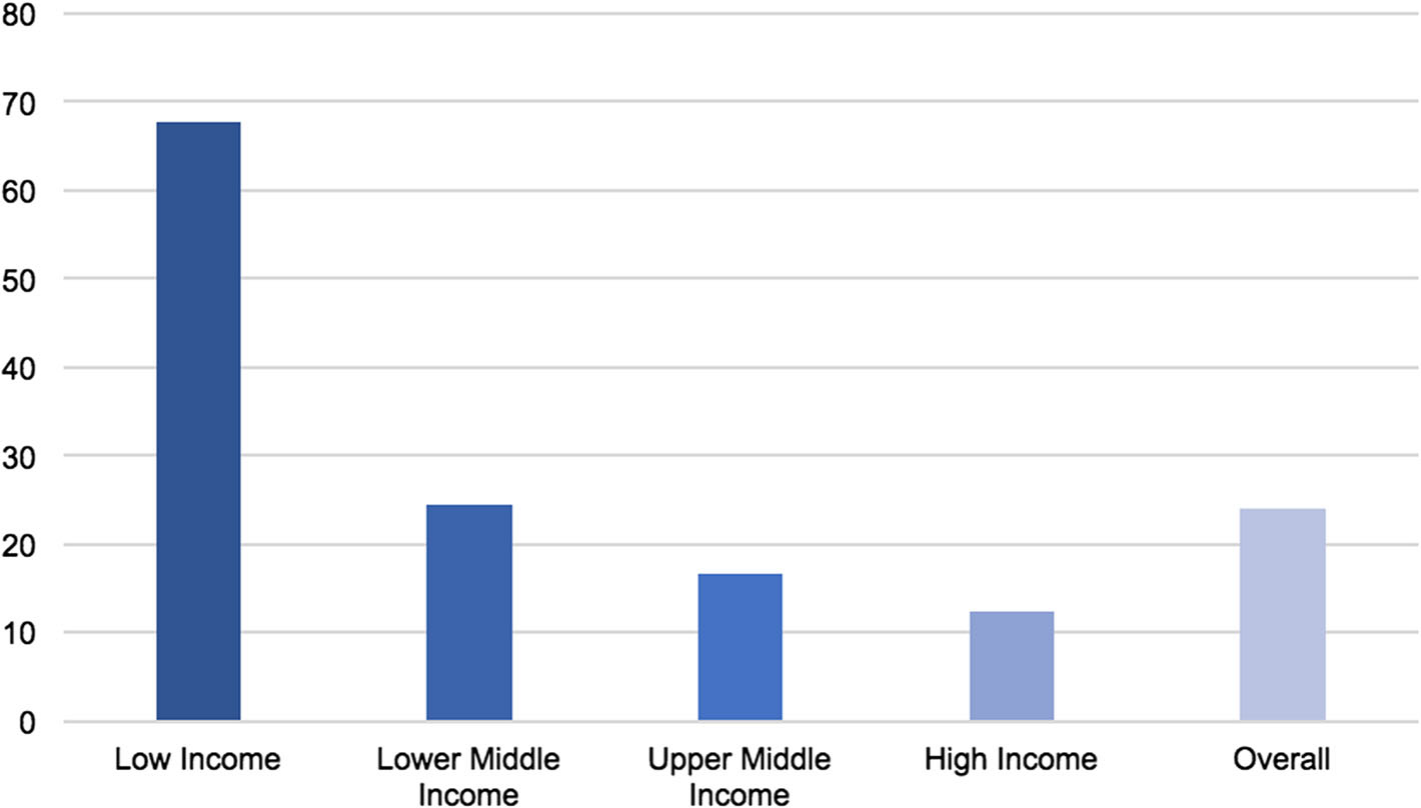
Percentage of countries with no available PA/SB policy studies; overall and by economic standard

Sixty-nine percent (*n* = 438) of 635 country-specific policy analyses focused on high-income countries, out of which 63% (*n* = 277) related to European Union (EU) member states (Fig. 2). No studies were identified for ten out of 81 high-income countries: the Bahamas, the British Virgin Islands, Channel Islands, Curaçao, Gibraltar, Isle of Man, Lichtenstein, Saint Maarten (Dutch part), Taiwan, and Turks and Caicos Islands. For 23 high-income countries only one country-specific policy analysis was found; with most of those findings arising from the GoPA’s *1st Physical Activity Almanac* [32]. Middle-income countries were investigated in 29% of country-specific policy analyses, and low-income countries in only 2%. For 21 out of a total of 31 low-income countries globally, and 22 out of 109 middle-income countries, no PA/SB studies were found (Fig. 3). The most eSxtensive policy review for low- and middle-income countries was performed by Lachat et al. [140]. They assessed the existence and content of governmental NCD, health, or nutrition policy documents from 83 WHO member states. However, the paper includes brief findings related to PA policies for only 35 countries. For 7% of all low- and middle-income countries presented in the current review - namely Cambodia, Djibouti, Jamaica, Madagascar, Mauritius, Niger, and the Philippines - findings on PA/SB policy were only available from the Lachat et al. [140] paper.

For 63 countries, only descriptive data, stating the existence and/or name of a policy document was found. More detailed analysis of PA related policies were available for Australia [6, 187], Brazil [96, 187], Canada [95, 187, 219], Chile [186], England [40], Finland [10, 39, 84, 187], France [82, 172], Italy [84], Mexico [153], the Netherlands [187], New Zealand [187], Norway [84], Portugal [84], Scotland [187], Slovenia [84], Switzerland [84, 187], and the USA [113]. Analyses of sport or leisure policies that contain substantial information on PA policies were available for Canada [76], Chile [81], China [146, 203], Cameroon [93], Czech Republic [139], England [76, 196], Germany [76], Malaysia [66], the Netherlands [200], New Zealand [66, 168], Norway [76, 195], Portugal [94], the UK [112, 156], and Vanuatu [135] (Additional file 3).

Only 22% of included studies mentioned SB as part of policy, and just one recent study analysed policies related to SB independently of PA policies [38]. Specifically, mentions of SB related policies/guidelines were found in research for Australia [190], Belgium [38], Canada [33], Finland [39], Hong Kong [124], Ireland [118], Iceland [127], Malta [127], New Zealand [149], Russia [127], Switzerland [127], and Sweden [38] (Additional file 3).

**Fig. 4:**
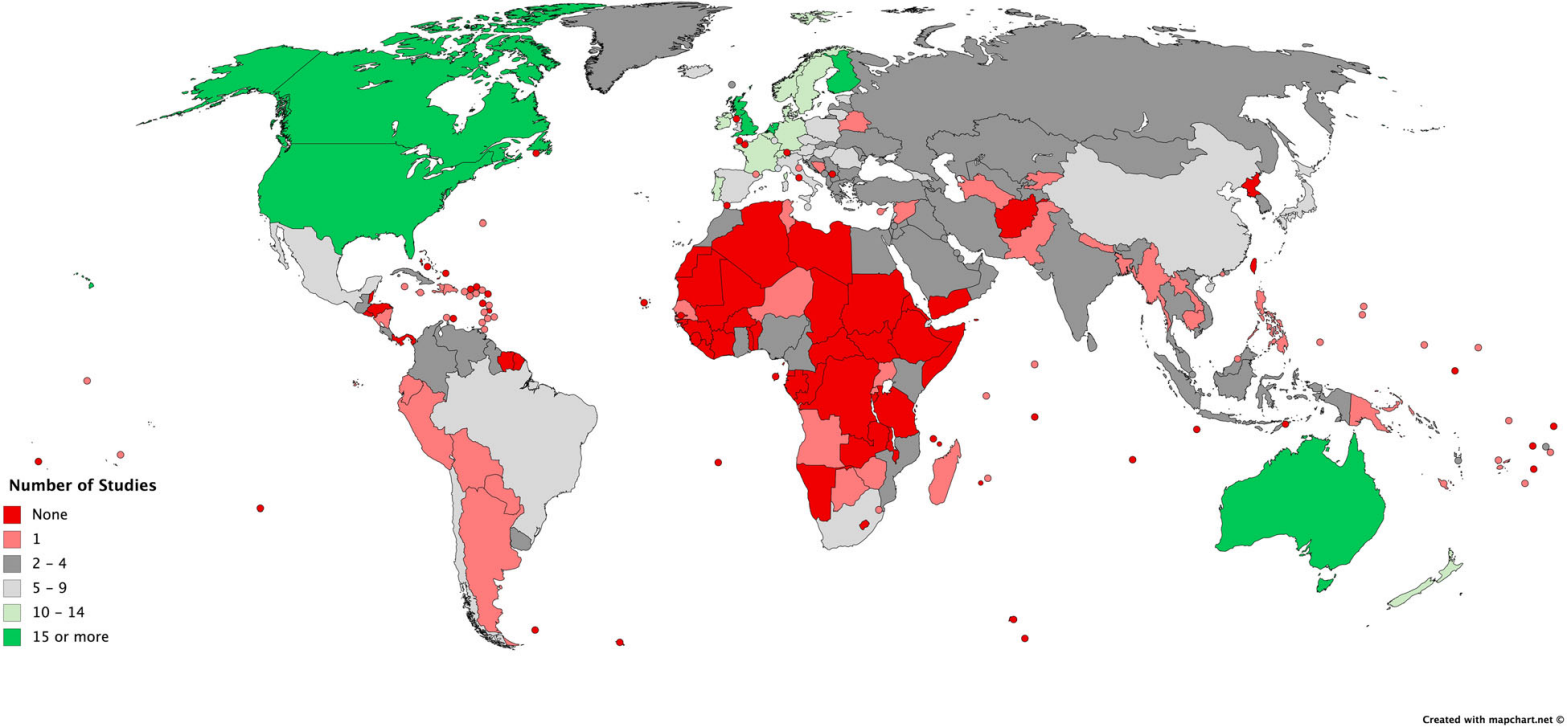
The global distribution of physical activity and sedentary behaviour policy research

The distribution of PA/SB policy research across countries is presented in Fig. 4. England, Canada, and Finland have been researched the most. Brazil and Mexico were the most represented countries from the Latin American and Caribbean region. In this region, no data were found for Belize, El Salvador, French Guiana, Honduras, Panama, and Suriname. From Sub-Saharan Africa, the most data were available for South Africa. However, Africa in general, both North and Sub-Saharan is the continent with least research found. From the Middle East, Yemen was the only country for which data were not found. The majority of research (55%) concerned European countries. For England, Finland, the Netherlands, and Scotland we found 15 or more studies. Most of the research was in regard to countries in Northern and Western Europe, with on average ten studies per country. Southern Europe had on average five studies per country and Eastern Europe four. From East Asia and Pacific region most data were found for Australia. China was the most researched Asian country.

### Conceptualisation of policy, frameworks and methods

A definition of policy, public policy, health policy, and/or PA policy was found in 13% of all included publications. A list of all definitions found in the publications is provided in Additional file 5 [6, 8, 11, 12, 16, 26, 37, 39, 40, 43, 44, 54–56, 62, 86, 92, 95, 103, 113, 129, 140, 153, 168, 171, 185, 187, 218, 228, 229, 231, 238–245]. The most commonly used definition of PA policy was originally proposed by Bull et al. [26]. The conceptualisation of policy varied across studies and often even within the same study. Only 15% of the included studies used a conceptual or theoretical framework. Kingdon’s Multiple Streams framework was used in four studies [116, 154, 169, 171]. Elite theory [203], multilevel model of PA promotion [185], figurational sociology [200], institutional change theory [108], the Theoretical Domains Framework, and the Behaviour Change Techniques Taxonomy [39] are among the other frameworks that were used. All four selected doctoral dissertations were based on conceptual/theoretical frameworks [66, 107, 168, 219].

The included studies used a variety of methods for data collection and analysis of PA/SB policy (Additional file 2). All studies relied on some form of literature review. Expert review was used in 46% of the studies. Content analysis of documents was used in 6% of the studies. Interviews (mainly semi-structured) were used in 9% of the studies. Some studies combined both content analysis of interviews and content analysis of documents [115–117, 139, 154]. Interviews were combined with focus groups in two studies [139, 185], and a focus group was combined with content analysis of documents in one study [38]. Discourse analysis was used in 2% of the studies [73, 107, 141, 168], among which half also used interviews as their research method [107, 168]. A case study design was employed in 6% of the studies. A number of studies did not clearly specify their research methods.

## Discussion

This is the first systematic scoping review of global PA/SB policy research. We found that PA policy research is much more developed than it was previously considered. However, there are few examples of policies that included SB. Three key issues were identified that should be addressed in further research: (i) there is a lack of PA/SB policy research in low- and middle-income countries, which is an important limitation of the current body of evidence; (ii) the definition of policy varied significantly across studies, and most studies did not rely on any theoretical framework, which may impede cross-study comparisons; and (iii) studies have used a variety of methods to collect data and analyse policy, which may also cause problems with comparability. Each of these future research directions are discussed further below.

Different studies largely differed in their focus and aims. Accordingly, the depth of analysis varied significantly across studies. The studies that mainly focused on monitoring policies, such as Ramirez Varela et al. [32], may be useful for providing a broad picture on PA policies globally. Studies that reviewed documents and their content in one region, such as Ceccarelli [90] and Kahlmeier et al. [127], can serve as a comparative overview of best practices and a good starting point for further research and more elaborated analyses of national policy. Detailed comparative studies on policies in a few countries, such as Bergsgard et al. [76], may be useful for understanding why some countries are more successful in PA promotion than others. Studies that critically assess PA policy in a single country, for example Milton and Bauman study for England [40] and Craig for Canada [95], may be useful for researchers and policy makers interested in the country’s policy situation and possible ways of improving it. Studies focusing on detailed assessment of one policy document, such as Pérez-Escamilla [167], may provide grounds for improving the documents and may be useful for informing the development of similar policy documents in other countries.

### Towards more research in low and middle-income countries

Most research was conducted to analyse PA/SB policies in high-income countries, whilst low- and middle-income countries are significantly underrepresented within PA/SB policy research. Most available findings for low- and middle-income countries are provided in the GoPA’s *1st Physical Activity Almanac*; hence this publication can be considered an important contribution to the development of PA policy research in these countries. For 17 high-income countries and 36 low- and middle-income countries, the only data we found were from GoPA country cards. Thus the country cards might be considered an important starting point for guiding PA policy developments in countries around the world. However, they merely include a statement about the availability (Yes/No) of the national or subnational PA plan (*n* = 47), the inclusion of PA within a broader NCD policy (*n* = 16), and the name of the available policy document (*n* = 76) as opposed to a detailed analysis of PA/SB policy status.

Another important study providing data for low- and middle-income was a review conducted by Lachat et al. [140], where PA/SB policies were analysed in the broader context of NCD prevention, together with nutrition-related policies. However, due to the fact that this study had a wider scope, only limited data were provided specifically on PA/SB policy. Policy actions and targets related to PA promotion were extracted from the respective documents, so unlike GoPA’s country cards, this study reports on some specific content of the policy documents. However, this review [140] provided no references for the reviewed policy document for Costa Rica, Madagascar, Guatemala, Solomon Islands, and Djibouti, which may limit the usability of their findings in future research on PA/SB policies in these countries. Another limitation of this review is that, while reporting on whether inactive lifestyle was discussed within policy documents, it did not distinguish between SB (nowadays defined as prolonged sitting) and inactive lifestyle (traditionally defined as lack of PA). This may cause confusion between the two concepts that the current epidemiological research clearly differentiates [25]. Clearly, more studies comprehensively reviewing PA and SB policies in low- and middle-income countries are needed.

General information on national PA/SB policies can also be found in studies from other sectors, for example NCD prevention [28, 246–249]. However, the depth of information they provide on PA/SB policies is often limited, as these policies are not in their main focus.

Furthermore, for large high-income countries, such as Canada, USA and Australia, a number of studies that analysed subnational (that is state, territorial, provincial, municipal, regional or local PA policies) were identified during the study selection process. For example, in the USA, a number of studies related to school district PA policies were found. Such studies may provide very useful information for PA/SB policy development at a local level and should, therefore, continue to be conducted in countries of both higher and lower economic standard. A separate scoping review of subnational PA/SB policy research is warranted as a systematic assessment of studies on this topic was beyond the scope of the current paper.

Taking into account that, for 53 countries around the world, no PA/SB policy studies were found, continued efforts in PA/SB policy development and research are needed. However, this might also be due to language restrictions, as this review included only studies with titles and abstracts in the English language. Further research should pay special attention to the low- and middle-income countries and those high-income countries with little or no available data.

### Towards a standardised conceptualisation of PA/SB related policies

Policy was differently conceptualised across different studies. In 2006, Schmid et al. wrote that “public health policy around PA remains poorly defined and developed” [16]. Given that only 10% of the selected studies were published before the Schmid et al. paper, the statement about the poor development of PA policy may not be true anymore. However, taking into account the issues with defining and conceptualising PA and SB policy across the studies included in this review, the Schmid et al. statement about the generally poor definition of PA policy remains valid. Schmid et al. conceptualised policy, reflecting political and social commitment, at three levels: (i) formal written codes, regulations or decisions holding legal authority; (ii) written standards that guide choices; and (iii) unwritten social norms that impact behaviours [16]. Among the currently reviewed studies that provided an operational definition of policy, the vast majority conceptualised it within the Schmid et al.’s first level. Many studies relied on the definition of policy provided by Daugbjerg et al. that conceptualises ‘policy’ as a ‘policy document’, that is a “written document that contains strategies and priorities, defines goals and objectives, and is issued by a part of the administration” [11]. This definition was later used as the working definition in the WHO and the European Commission in their joint reports of the National Information Focal Points meetings [228]. Rütten et al. for example stated that their approach is grounded on a broader definition of policy than the one proposed by the WHO, which also includes informal institutional procedures, arrangements and rationales for action on health- related issues [185]. The most often used definition of PA policy was proposed by Bull et al., which defines it as a “formal statement that defines physical activity as a priority area, states specific population targets and provides a specific plan or framework for action” [26]. In most cases, studies focused only on public sector policies, that is, “governmental statements”, whilst somewhat less often they also included written statements of NGOs, international organisations, and professional bodies. Some studies, such as Christiansen et al. and Daugbjerg et al., clearly distinguished between policy and other documents such as strategies, action plans, and guidelines [11, 44]. These two studies as well as Al-Bahlani and Mabry [62] made a distinction between policies and legislation. Unlike, for example Coenen et al. [38] who under the category “policy documents” included guidelines, legislation, directives, and codes of practices. Seppälä et al. [39] under “policy papers” also included guidelines, good practice guides, strategies, and action plans. A number of studies did not clearly differentiate between interventions, policies, and policy actions. Some studies, such as Milton and Bauman [40] conceptualised PA policy more comprehensively and considered national recommendations on PA levels, national targets and goals related to PA, public education on PA, and PA surveillance and monitoring as key aspects of national PA policy, whilst others, such as Pate et al. [37] defined it more narrowly as formal written documents providing guidelines on public PA promotion.

Various understandings and conceptualisations of PA policy within and between studies may create confusion within the field and negatively affect comparability of findings, but may also be part of an evolutionary process of reaching a consensus on what PA policy is. However, political scientists have agreed there is likely never to be a universal definition of policy. Policy is a flexible concept used differently in different contexts and on different occasions. It is a “continuing process of social action and interaction” and there are a lot of different ways in which people perceive or perform policy [35]. Using the term “policy” in different ways across different contexts is not necessarily a problem [35]. However, we believe defining it within every specific academic discourse can be beneficial and would significantly contribute to the reduction of analytical weaknesses present in some PA/SB policy studies that, by not providing a clear operational definition of policy, often fail to properly define their object of policy analysis.

The conceptualisation of PA/SB policy depends also on the definition of PA and SB. Even though scientific consensus seems to have been achieved [25], PA is still often confused with sport, physical fitness, and exercise. The inconsistency regarding the definition of SB is even larger, probably because SB research is a much younger field than PA epidemiology. The interchangeable use of the terms ‘physical inactivity’, ‘sedentary lifestyle’, ‘screen-time’, and ‘sedentary behaviour’ is still very common among scholars [21]. The Sedentary Behaviour Research Network (SBRN) initiated the *Terminology Consensus Project* and suggested definitions of several terms related to SB [25]; yet definitions of some common terms, such as “sedentary lifestyle”, have still not been clarified [25].

Finally, only 15% of the PA/SB policy studies relied on theoretical or conceptual frameworks to support their analyses. It is evident that PA/SB policy research should be more grounded in existing frameworks. For example, in 2006, Schmid et al. developed the *Framework for PA Policy Research*. Although this framework was mentioned in several studies [8, 11, 44, 103, 171, 185, 218, 219], only two studies based its content analysis grid on this framework [11, 44]. Using some of many available theories, frameworks, methods, and concepts available from political science and other established disciplines could positively contribute to the further improvement and standardisation of PA/SB policy research. It should be noted, however, that the diversity of approaches and definitions may sometimes be considered desirable, especially in young fields of research. Advancing to standardisation too soon might hinder the development and exploration of some potentially useful approaches.

### Towards a standardised policy analysis

The so-called “policy science” and its main component, policy analysis, have been developing since the 1950s when Harold Lasswell’s seminal book *The Policy Sciences – Recent Developments in Scope and Method* was published [250]. However, due to the lack of a universally accepted definition of policy, there is also no universally accepted method to perform policy analysis. This review revealed that the methods used for PA/SB policy research are far from being standardised and that the form of research outputs in this area largely depended on individual approaches. The sage words of the authors of *The Australian Policy Handbook*: “Policy analysis is a balance between art and science.” [251] can, therefore, also be applied to research analysing PA/SB policies. While some claim there is no difference between policy analysis, policy assessment, and policy evaluation, some made guidelines on how each one of these should be performed and differentiated from the others [252, 253]. Policy analysis as a *craft* “draws on intuition as much as on method” [254]. Considering that PA/SB policy research is at least 40 years younger than “policy science”, it is understandable that it still draws more on intuition than on method. This notion is grounded in the fact that most of the studies included in this review did not rely on specific, conventional policy research methods but usually on narrative literature reviews and expert reviews.

The challenges in policy analysis were clearly outlined in some reviewed studies. For example, the Active Healthy Kids Report Card’s indicator titled *Government Strategies and Investments* was assessed against three benchmarks: (i) “evidence of leadership and commitment in providing physical activity opportunities for all children and youth”; (ii) “allocated funds and resources for the implementation of physical activity promotion strategies and initiatives for all children and youth”; and (iii) “demonstrated progress through the key stages of public policy making (i.e., policy agenda, policy formation, policy implementation, policy evaluation and decisions about the future)” [34]. However, this indicator has been reported as “difficult to grade” [33]. In the first comparative ‘Global Matrix’ of grades from 2014, one-third of the countries did not grade this indicator and marked it as incomplete [33]. In the second ‘Global Matrix’ it was reported that only six out of 38 countries marked this indicator as incomplete [34]. Even though the number of countries that assigned grades was higher in the second matrix than in the first one, several Report Cards stated that this indicator is one of the hardest to grade. Some of the reported reasons were: a lack of agreed assessment criteria [147, 149] or specific international recommendations [215]; no well-founded and clear criteria or benchmarks to outline which amount of investments is acceptable or which policy is effective [87]; and the perception that the Report Cards are not fit for policy evaluation purposes [121]. In the results from Qatar’s Report Card, it was stated that the grade was assigned “as in most countries” based on the “presence” of national investments and strategies related to children and youth’s health and PA [63]. This may not be considered the most informative approach to PA/SB policy analysis.

To support standardised analysis of national policy approaches to PA, the HEPA Europe expert group developed a comprehensive instrument entitled *Health Enhancing Physical Activity Policy Audit Tool* (HEPA PAT) [30, 85], structured around 17 key elements for a successful national approach to PA promotion. Prior to its development, there was no “standardised instrument to capture the relevant policy information in a standardised way or to collate more in-depth data” [30]. HEPA PAT is one of the rare tools that, in addition to PA, also informs on SB policies. The protocol recommends that PAT is completed using a collaborative process and involving multiple sectors. It suggests that responses from all relevant sectors are collected and reviewed collectively and that the process of completion itself can support and strengthen policy development. However, the early experiences of countries developing the HEPA PAT found that between three and 6 months are needed to complete the whole process [42] which is probably the main reason why since its development it has only been used in three other studies [172, 232, 255]. There are, however, promising ongoing initiatives that will likely ensure the implementation of HEPA PAT in more countries internationally. It is also important to mention that the primary purpose of HEPA PAT is limited to policy audit and therefore, it cannot be used for the policy assessment (or grading as in the case of the Active Healthy Kids Report Cards). There seems to be a need for the development of a tool which would allow for rating or assessment of the success and progress of national policies related to PA/SB [30]. More coordinated work on a standardised approach to international analysis of PA/SB policies would significantly contribute to the further development of this research area.

### Strengths and limitations of the review

The key strengths of the current review include: (i) the search was conducted through a range of bibliographic databases, reference lists of included articles, and relevant websites, which reduced the likelihood of missing relevant publications; (ii) we used an inclusive search syntax and broad eligibility criteria that allowed us to identify and include relevant studies on a wide range of PA/SB policy topics; (iii) the assessment of eligibility of studies was done in duplicate, which reduced the likelihood of bias in study selection; (iv) we clearly stated the definitions of policy and policy analysis used for the purpose of this review; and (v) full-texts of 12 publications were translated from their original languages into English to allow for data extraction.

This review is subject to some limitations. Firstly, although the literature search was done with no language restrictions, we were able to include only publications with titles and abstracts in English. This may have resulted in the omission of some relevant publications. It should be noted, however, that we included 12 publications with full-texts in languages other than English. Secondly, we did not conduct a formal assessment of study and evidence quality. This was not possible to be done in a systematic fashion, because the included studies were conducted using a wide variety of study designs and methods. Nevertheless, based on the extracted data, we provided a general assessment of the overall completeness of evidence. Finally, we did not conduct an in-depth analysis of PA/SB policies for each specific country. Although such an analysis would be of great value for future research and policy initiatives, it was beyond the scope of this review. Nevertheless, we summarised findings of the included studies for a total of 168 countries.

## Conclusion

The results of this systematic scoping review show that PA policy research is much more developed than it was considered several years ago. Research around SB policies is still in its infancy, but it seems to have experienced some positive progress in the last few years. There are still a large number of countries with no or very little research on PA/SB policy, particularly among those with low or middle income. Increased efforts should be made to include such countries into academic discussion on PA/SB policy. Future PA/SB policy studies should also aim towards a clearer conceptualisation of policy, greater reliance on existing theoretical frameworks, and the use and further development of standardised methods for PA/SB policy analysis.

## Additional files

Additional file 1: Full search syntaxes used for each database. (PDF 90 kb)

Additional file 2: Description of studies analysing indicators, development, and content of national-level physical activity and sedentary behaviour policies. (PDF 434 kb)

Additional file 3: Summary results of studies analysing indicators, development, and content of national-level physical activity and sedentary behaviour policies: country-specific findings. (PDF 836 kb)

Additional file 4: Summary results of studies analysing indicators, development, and content of national-level physical activity and sedentary behaviour policies: international findings. (PDF 138 kb)

Additional file 5: Definitions of policy in general, public policy, physical activity policy, health policy, and policy document included in studies analysing indicators, development, and content of national-level physical activity and sedentary behaviour policies. (PDF 114 kb)

## Abbreviations

CDC: Centers for Disease Control and Prevention
EU: European Union
GoPA: Global Observatory for Physical Activity
HEPA PAT: Health-enhancing physical activity policy audit tool
HEPA: Health-enhancing physical activity
NCD: Noncommunicable disease
NGO: Nongovernmental organisation
PA: Physical activity
SB: Sedentary behaviour
UK: United Kingdom
UN: United Nations
USA: United States of America
WHO: World Health Organization
