# Supplementary material for: A global systematic scoping review of studies analysing indicators, development, and content of national-level physical activity and sedentary behaviour policies
Source: Int J Behav Nutr Phys Act. 2018 Nov 28;15:123. doi: 10.1186/s12966-018-0742-9 (PMC6263060; doi:10.1186/s12966-018-0742-9)
Supplement: Supplementary file 1 — Full search syntaxes used for each database. (PDF 90 kb) [file 12966_2018_742_MOESM1_ESM.pdf]

Additional file 1 – Full search syntax used for each database

Scopus:

title-abs-key("physical activity" or "physical inactivity" or sedentar* or sitting) and title-abs-key(policy or policies)

PubMed/MEDLINE:

("physical activity"[tw] OR "physical inactivity"[tw] OR sedentar*[tw] OR sitting[tw]) AND (policy[tw] OR policies[tw])

Web of Science, SportDiscus (through EBSCOhost) Open Access Theses and Dissertations (OATD), Networked Digital Library of Theses and Dissertations (NDLTD):

("physical activity" or "physical inactivity" or sedentar* or sitting) and (policy or policies)
