# Supplementary material for: A global systematic scoping review of studies analysing indicators, development, and content of national-level physical activity and sedentary behaviour policies
Source: Int J Behav Nutr Phys Act. 2018 Nov 28;15:123. doi: 10.1186/s12966-018-0742-9 (PMC6263060; doi:10.1186/s12966-018-0742-9)
Supplement: Supplementary file 2 — Description of studies analysing indicators, development, and content of national-level physical activity and sedentary behaviour policies. (PDF 434 kb) [file 12966_2018_742_MOESM2_ESM.pdf]

Additional file 2 – Description of studies analysing indicators, development, and content of national-level physical activity and sedentary behaviour policies

Study	Scope	Focus	Period	Short description and methods
Adeniyi et al., 2016 [59]	National (1 country)	A wide range of PA indicators for children and youth in Nigeria	2013 – 2016	This article reported on the results from a *RC on PA for Children and Youth*. The RC was created by a research work group by reviewing academic and grey literature (e.g. government documents and dissertations). It includes an assessment of *Government, Nongovernmental Organizations, and Private Sector (Strategies and Investments)/Policy* as one of ten PA indicators.
Aguilar-Farias et al., 2016 [60]	National (1 country)	A wide range of PA indicators for children and youth in Chile	n/a	This article reported on the results from a *RC on PA for Children and Youth*. The RC was created by a research work group that reviewed available evidence from government documents, publications, surveys, and data sets. It includes an assessment of *Government Strategies and Investments* as one of 11 PA indicators.
AHK Canada, 2016 [58]<br>Barnes et al., 2016 [71]	National (1 country)	A wide range of PA indicators for children and youth in Canada	best available current evidence	This *RC on PA for Children and Youth* and the associated journal article were created by the Healthy Active Living and Obesity research group and a RC research committee. Assessed sources included peer-reviewed literature, national surveys, and grey literature (e.g. online content and government and nongovernment reports). The RC includes an assessment of PA *Strategies and Investments (Government)* as one of 12 PA indicators.
AHK Canada, 2015 [57]	National (1 country)	A wide range of PA indicators for children and youth in Canada	best available current evidence	This *RC on PA for Children and Youth* was created by the Children's Hospital of Eastern Ontario research institute and a RC research committee. They synthesised data from the research literature and multiple data sources, mainly surveys. The RC includes an assessment of PA *Strategies & Investments (Government)* as one of 11 PA indicators.
AHK Canada, 2014 [56]<br>Gray et al., 2014 [111]	National (1 country)	A wide range of PA indicators for children and youth in Canada	best available current evidence	This *RC on PA for Children and Youth* and the associated journal article were created by the Healthy Active Living and Obesity research group and a RC research committee. RC development process includes an expert consensus and synthesis of the best available research, policy, surveillance, and practice findings. It includes an assessment of PA *Government Strategies and Investments* as one of ten PA indicators.
AHK Canada, 2013 [55]	National (1 country)	A wide range of PA indicators for children and youth in Canada	best available current evidence	This *RC on PA for Children and Youth* was created by the Children's Hospital of Eastern Ontario research institute and a research work group. RC synthesises data from the research literature and multiple data sources, mainly surveys. It includes an assessment of PA *Strategies and Investments / Policy - Federal Government Strategies and Investments* as one of 17 PA indicators.
AHK Canada, 2012 [54]	National (1 country)	A wide range of PA indicators for children and	best available current evidence	This *RC on PA for Children and Youth* and the associated journal article were created by the Healthy Active Living and Obesity research group and a research work group consisting of 11 experts. Healthy Active Living and Obesity research group conducted

Barnes et al., 2013 [72]		youth in Canada		a review of non-academic and academic literature. The research work group reviewed content and assigned grades for 24 PA indicators. The RC includes an assessment of *Federal Government Strategies* and *Federal Government Investments* as two indicators that belong to the *Policy* category.
AHK Canada, 2011 [53]	National (1 country)	A wide range of PA indicators for children and youth in Canada	best available current evidence	This *RC on PA for Children and Youth* was created by the Children's Hospital of Eastern Ontario research institute and a research work group that synthesised and reviewed data from the research literature and multiple data sources. It includes an assessment of *Federal Government Strategies* and *Federal Government Investments* as two out of 23 PA indicators that belong to the *Policy* category.
AHK Canada, 2010 [52]	National (1 country)	A wide range of PA indicators for children and youth in Canada	best available current evidence	This *RC on PA for Children and Youth* was created by a research group that synthesised and reviewed data from the various data sources such as studies, surveys, literature and policy reviews. It includes an assessment of *Federal Government Strategies* and *Federal Government Investments* as two out of 17 PA indicators that belong to the *Policy* category. Historical review of policies and summary of eight criteria required for effective policy implementation and development informed the assessment of these indicators.
AHK Canada, 2009 [51]	National (1 country)	A wide range of PA indicators for children and youth in Canada	best available current evidence	This *RC on PA for Children and Youth* was created by a research group that assessed several information sources including national data sets, industry reports, peer-reviewed research publications, and various media sources. It includes an assessment of *Federal Government Strategies* and *Investments* as one of 19 PA indicators.
AHK Canada, 2008 [50]	National (1 country)	A wide range of PA indicators for children and youth in Canada	best available current evidence	This *RC on PA for Children and Youth* was created by the AHK Canada staff and a research work group that identified, ranked and graded 23 PA indicators, based on available research, data and key issue areas. Consultations with key stakeholders who provided feedback also informed the development of the RC. It includes an assessment of *Progress and Government Strategies and Investments* as one of the indicators that belongs to the *Policy* category.
AHK Canada, 2007 [49]	National (1 country)	A wide range of PA indicators for children and youth in Canada	best available current evidence	This *RC on PA for Children and Youth* was created by a research work group that identified, ranked and graded 13 PA indicators, based on available research, data and key issue areas. Consultations with key stakeholders who provided feedback also informed the development of the RC. The RC includes an assessment of *Progress on Government Strategies and Investments* as one of the indicators that belongs to the *Policy* category.
AHK Canada, 2006 [48]	National (1 country)	A wide range of PA indicators for children and youth in Canada	best available current evidence	This *RC on PA for Children and Youth* was created by a research work group that reviewed the results from the RC published in 2005. It served as starting point for data collection, analysis and establishment of 14 PA indicators. It includes an assessment of *Federal Strategies and Investments* as one of the indicators. The indicator belongs to the *Policy* category that assessed government policy support for PA.

AHK Canada, 2005 [47]	National (1 country)	A wide range of PA indicators for children and youth in Canada	best available current evidence	Canadian national PA Symposium engaged leading experts in PA research and was a starting point for creation of this *RC on PA for Children and Youth*. RC is based upon analyses of information obtained from multiple cycles of the several surveys, and various research studies and data grouped in the six category areas. It includes an assessment of *Federal Strategies and Investments* as one of 14 PA indicators. The indicator belongs to the *Policy* category.
Akinoroye et al., 2014 [61]	National (1 country)	A wide range of PA indicators for children and youth in Nigeria	n/a	This *RC on PA for Children and Youth* was created by a technical report committee that identified and reviewed relevant published and unpublished literature. It includes an assessment of *Government Strategies and Investments* as one of 14 PA indicators.
Al-Bahlani & Marby, 2014 [62]	National (1 country)	Legislation for NCD prevention in Oman	n/a	The study provides an overview of documents related to NCD prevention. A search of documents in Arabic and English was conducted using Internet search engines and through various websites of relevant institutions (e.g. Gulf Cooperation Council, ministries of legal affairs, ministries of health etc.). The review also included the strategies and reports provided by the Ministry of Health and public health professionals.
Al-Kuwari et al., 2016 [63, 64]	National (1 country)	A wide range of PA indicators for children and youth in Qatar	2004 – 2014	This *RC on PA for Children and Youth* and the associated journal article were created by a research work group that identified and reviewed relevant published and unpublished literature. The work group discussed the obtained information with a stakeholder group that provided additional data. The RC includes an assessment of *National Policy*, *Strategy, and Investment* as one of nine PA indicators.
Alderman et al., 2007 [65]	National (1 country)	Laws to combat obesity in the USA	n/a	The study explores the history of public health legislation and how law interacts with public health aspects of children's healthy nutrition and PA in the USA. Study outlines the complex interaction of policy makers, advocates, judiciary and legal doctrines, and the food industry with regard to obesity issues.
Aman, 2005 [66]	International (2 countries)	Development of leisure policies in New Zealand and Malaysia	1970 - 2020	This doctoral dissertation is a comparative analysis of leisure policies in New Zealand and Malaysia. Data was obtained through archival, library research of publicly available documents and through semi-structured interviews with 21 "key players" from each country.
Amornsriwatanakul et al., 2016 [67, 68]	National (1 country)	A wide range of PA indicators for children and youth in Thailand	Survey data collection: June 2015 – January 2016	This *RC on PA for Children and Youth* and the associated journal article were created by a national committee comprising experts from key stakeholders. It includes *Government Strategies, Policies, and Investments* as one of nine PA indicators. Government published and unpublished reports were reviewed to inform this indicator. Committee members from government authorities provided additional input for this indicator. Other indicators were mainly informed by a survey that was conducted as a part of this RC.
Ballesteros Arribas et al., 2007 [70]	National (1 country)	A nutrition, PA and obesity	n/a	The study provides a description of the content and development of a policy document; the *Spanish strategy for nutrition, physical activity and the prevention of obesity* (NAOS

(in Spanish language)		strategy in Spain		Strategy), issued in 2005.
Bell-Altenstad & Vail, 1995 [73]	National (1 country)	Sport policy in Canada	n/a	The study is a policy discourse analysis of Federal Government of Canada's sport policy development in regard to women.
Bellew in Bull et al., 2004 [74]	National (1 country)	Documents to inform National Strategy for PA in Australia	Documents from 1999 till 2003	The study is a review of 22 framework/strategy documents related to PA, conducted to inform the development of the National Strategy for PA. It includes both federal- and state/territory-level government and non-government documents.
Bellew et al., 2011 [8]	International	Policy actions for PA promotion	2009 – 2010	The article describes national and local public policy actions and programmes for PA promotion in Australia, Brazil, Canada, Europe, Great Britain, Nordic countries, Switzerland, and the USA.
Bellew et al., 2008 [6]	National (1 country)	Australian PA policy	1996 – 2006	The study is a comparison of Australian policy with international PA policies in seven countries assessed using the proposed HARDWIRED policy definition criteria. Literature and policy reviews were combined with questionnaires sent to purposively sampled experts. The historical development including key PA policy events in Australia is also presented in the paper.
Bercovitz, 1998 [75]	National (1 country)	Active living policy in Canada	n/a	The study includes a critical analysis of Canada's Active Living policy and an overview of its evolution.
Bergsgard et al., 2007 [76]	International (4 countries)	Sport policy (including "Sport for All")	n/a	The book includes a comparative study of sport policies in four countries – Canada, England, Germany, and Norway. Document analysis, qualitative content analysis, and interviews with senior policy actors and academic observers in the area of sport policy were conducted. Historical and political contexts are also presented.
Bornstein et al., 2014 [78]	National (1 country)	National PA plan in the USA	n/a	The study comprehensively describes the full development process of the document titled *National Physical Activity Plan for the United States*, to inform further development of community and state-based PA plans within the USA and national PA plans in other countries.
Bornstein & Pate, 2014 [77]	National (1 country)	National PA plan in the USA	n/a	The study describes the development process and the content of the document titled *U.S. National Physical Activity Plan*. It also reflects on the current status of and future perspectives for the national plan.
Bornstein et al., 2009 [79]	International (6 countries)	National PA plans	n/a	The study is a review of six comprehensive PA national plans published in English from Australia, Northern Ireland, United Kingdom, Sweden, Scotland and Norway. The documents were found in electronic databases. Their content, characteristics, and development processes were analysed to inform the development of the USA PA plan.
Branca et al., 2007 [80]	International (19 countries)	Obesity prevention in general	n/a	This book is about the obesity challenge in Europe. One of its chapters is related to *National policies in the European Region*. Twenty-six national policy documents on PA, obesity and nutrition in English from 19 countries were analysed. Policies from 17

				additional countries were briefly discussed at the end of the chapter, but were not thoroughly analysed.
Bravo & Silva, 2014 [81]	National (1 country)	Sport policy in Chile	n/a	The study includes an overview of the historical development and the current status of Chile's sport policy. It examines key legislative documents and their influence on sport.
Bréchat et al., 2009 [82] (in French language)	National (1 country)	National programmes for PA and sport in France	2001 - 2006	The study is a review of 14 national programmes/actions on PA and sports, conducted to assess the rationale used to develop a public health project in France. Data was collected using semi-structured interviews with 15 PA/sport experts who had experience in developing at least one PA/sport-related action plan or document that were obtained during interviews or through Internet search. The design, implementation, and evaluation of programmes were analysed.
Brown et al., 2011 [43]	International (2 countries)	Evidence on PA for older people and PA guidelines for older people in New Zealand, Australia and international findings	Documents published since 2004 (chapter 9)	The study is an extensive literature review of evidence on PA and of the current PA guidelines for older people. In chapter nine titled *International guidelines, policies and principles*, various databases (e.g. the WHO database) and Internet sources (e.g. websites of ministries and governments) have been searched for documents published in English language. Two systematic reviews of policies, 22 national policy or strategy documents, and seven position stands/scientific statements from professional bodies were included in the review. The documents have been assessed using the Appraisal of Guidelines for Research and Evaluation quality grading.
Bull et al., 2014 [83-85]	International (7 countries)	HEPA policies	2009-2012 (data representativ e up to May 2011)	The study compared the development processes, content, and implementation of HEPA policies in seven EU countries (Finland, Italy, the Netherlands, Norway, Portugal, Slovenia, Switzerland). The data was collected using the HEPA PAT. For each country, a leading academic, a representative of relevant institute or (sub)national government official, was in charge for completion of the HEPA PAT. Directed content analysis was used to analyse collected data.
Bull et al., 2004 [26] Schöppe et al., 2004 [187] Bull et al. in Bull et al., 2004 [86]	International (7 countries + Finland)[1]	National PA policy	Policy documents from 1966 - onwards	The study is an analysis of the development and the content of PA policy in Australia, Brazil, Canada, the Netherlands, New Zealand, Switzerland, and Scotland assessed against 11 specific criteria for successful PA policy and action plans. The data on policy were extracted from articles, national policy documents, and grey literature obtained through an electronic literature search.
Burghard et al., 2016 [87, 88]	National (1 country)	A wide range of PA indicators for children and youth in the Netherlands	2010 – 2014	This *RC on PA for Children and Youth* and the associated journal article were created by a research work group that synthetised the best available research, surveillance, policy and practice findings. The RC includes an assessment of *Government Strategies and Investments* as one of nine PA indicators. Multiple government documents informed this indicator.

[1] Findings for Finland were presented only in Schöppe et al., 2004.

Cavill et al., 2006 [89]	International (3 countries)	PA promotion and policy	n/a	The study provides guidelines for a systematic evidence-based approach to PA promotion. The approaches to policy development and promotion of PA in England, Finland, and Switzerland were explored and described.
Ceccarelli et al., 2011 [90]	International (34 countries)	Policy statements related to obesity, improvement of diet, and PA	documents adopted until 2008	The study is a review of quantitative and qualitative documents published in English, French, Italian, Spanish, and Portuguese. Information about policies and interventions was mostly gathered from sources such as national public health institutions, health ministries, and the WHO Regional Office for Europe nutrition policy database.
Chen in Simonopoulos (ed.), 1997 [91]	National (1 country)	Policies on nutrition, fitness and "Sport for All"	n/a	This conference paper presents an unsystematic overview related to of nutrition, physical fitness, and "Sport for All" in China. One part of the paper is dedicated to the *Sports for All* plan.
Chimeddamba et al., 2015 [92]	National (1 country)	Policy documents for NCD prevention in Mongolia	Documents from 2000 – 2013	The study analysed 45 policy documents related to NCD prevention issued by the Mongolian Government. Documents were obtained through an Internet-based search including the websites of the Government, United Nations agencies, NGOs, research institutes, and by using Medline and Google Scholar. The literature review was complemented by expert consultations.
Christiansen et al., 2014 [44]<br>World Health Organization, 2011 [231]	International (15 EU member states)	Policies related to sport and HEPA	2000 - 2009	The study used policy content analysis of EU member states' national (and subnational if no national available) policy documents related to HEPA and sport. Documents were obtained by: search of International inventory of documents on PA promotion; Internet search of websites of relevant ministries; Google search by key words; country templates completed by National Information Focal Points. A content analysis of policies was performed on 25 documents.<br>Study is a part of "Promoting networking, exchange and greater synergy between sport and health-enhancing physical activity sectors" (NET-SPORT-HEALTH) project.
Clarke & Ojo, 2017 [93]	National (1 country)	Sport policy in Cameroon	n/a	The study presents an overview of the historical development and current status of the sport policy in Cameroon. It examines, for example, the recent political past, organisation of sport, physical education, importance of football, and "non-state-led" sport.
Coenen et al., 2017 [38]	International (9 countries)	SB policies	1989 – 2015	The study reviewed health and occupational safety policies relevant to occupational SB. A qualitative synthesis of 119 policy documents written in English or Dutch was performed.<br>The study included documents from: Australia, Canada, Denmark, Finland, the Netherlands, New Zealand, Sweden, the United Kingdom, the USA; international organisations; and pan-European occupational health and safety or related agencies. Triangulation of data was provided by key informant participants of focus groups.

Costa Januario et al., 2012 [94] (in Portuguese language)	National (1 country)	PA and sport in government programmes in Portugal	1987 - 2011	The study is a descriptive analysis. It is a legal interpretation of PA and sport promotion and support mentioned in the Portuguese Governments' documents (*Programa do Governo Consitucional*). It provided assessment of 14 programmes/agendas published in each government term.
Craig, 2011 [95]	National (1 country)	PA policy in Canada	1981 – 2011	The study analyses the evolution of PA policy in Canada. Historical and current policy documents at federal, territorial and provincial were found through literature review, a search of non-government and government websites, and by contacting government officials responsible for PA. Content, barriers, results, and success factors of the policy were analysed.
da Silva, 2007 [96] (in Portuguese language)	National (1 country)	PA and sport policies throughout history in Brazil	End of 19th century until 1950s	The study analyses the role that PA/sport and PA/sport policy played in strengthening the Brazilian national identity and, consequently, in legitimising some of the Government actions. It is an in-depth historical narrative analysis of Government policy and policy documents related to PA and sport.
Daugbjerg et al, 2009 [11]	International (14 countries)	Policy documents related to PA promotion	Only the most recent documents included	The study is a content analysis of 27 policy documents from 14 countries in the European Region. It included national PA policy documents published in English. Documents were obtained from websites of the relevant ministries and health promotion agencies, subnational initiatives, and other relevant PA promotion projects and through a Google search. Results of the search were complemented by the relevant materials and findings from questionnaire data previously collected by the WHO. An overview of 49 policy documents on PA promotion from 24 countries is also presented, but only 27 documents were included in the content analysis.
de Villiers et al., 2010 [97]	National (1 country)	A wide range of PA indicators for children and youth in South Africa	2004 – 2010	This *RC on PA, Nutrition and Tobacco use for Children and Youth* was created by a scientific advisory panel that assessed: peer-reviewed published manuscripts; dissertations and theses; government-funded reports; and monographs. It includes an assessment of *Policies, programmes and interventions to promote physical activity* as one of PA indicators.
Dentro et al., 2014 [98, 99]	National (1 country)	A wide range of PA indicators for children and youth in USA	2003 – 2014 (data sources published)	This *RC on PA for Children and Youth* and the associated journal article were created by a research advisory committee that identified key publications and the best available data sources. It includes an assessment of *Government Strategies and Investments* as one of ten PA indicators.
Draper et al., 2014 [100, 101]	National (1 country)	A wide range of PA indicators for children and youth in South Africa	2010 – 2014	This *RC on PA and Nutrition for Children and Youth* and the associated journal article were created by a research work group that conducted a systematic review of dissertations, peer-reviewed literature, and grey literature. It includes an assessment of *Influence of Government,* that is, *Government – Strategies, Policies, Investments* as one of ten PA indicators.
Egger et al., 2001 [102]	National (1 country)	National PA Guidelines in Australia	n/a	The article describes the development process of the *National Physical Activity Guidelines for Australians*.

Eyler, 2011 [103]	National (1 country)	PA policy in the USA	n/a	The article described PA promotion through policy. It outlined PA policy research framework, theories related to PA policies, and promising PA policies in the USA.
Fullagar, 2003 [105]	National (1 country)	Health policy *(Active Australia* campaign)	n/a	The study developed a feminist analysis of the implications of the growing emphasis on active leisure promotion among women as a “sedentary” population. It assessed universal healthy lifestyle norms and health-promotion rationalities behind the *Active Australia*’s intention to mobilise women to become more active.
Fullagar, 2002 [104]	National (1 country)	Leisure and lifestyle in Australian health policy	n/a	The study provides a cultural analysis of government campaigns *Life be it*! and *Active Australia*. It offers an assessment of the ways discourses of healthy lifestyle and leisure have been produced through Australian health policy objectives and promotion.
Galaviz et al., 2016 [106]	National (1 country)	A wide range of PA indicators for children and youth in Mexico	2013 - 2015 (most data from that period)	This *RC on PA for Children and Youth* was created by an expert work group that conducted a literature search to identify: peer-reviewed literature; national surveys; an online PA related content; and grey literature, including non-government and government reports. It includes an assessment of *Strategies and Investments (Government and Non-Government)* as one of nine PA indicators.
Gillon, 2010 [107]	International (2 countries)	Active recreation policy and human rights-based approach	n/a	This master thesis explores a human rights-based approach applied on active recreation policy in New Zealand. Literature review, document analysis, and interviews with four key informants were undertaken. Document analysis was conducted on key policy documents in the United Kingdom and New Zealand using a human rights approach and a discourse analysis.
Gomez, 2015 [108]	International (2 countries)	Obesity policy	n/a	This case study compared responses to obesity by Brazilian and USA federal institutions. Qualitative data used in this study were: government documents; peer reviewed journal articles; and reports published by think tanks. The article addressed PA policies as an important part of government approaches and responses to obesity.
González et al., 2016 [109]	National (1 country)	A wide range of PA indicators for children and youth in Colombia	2005 – 2015	This article reported on the results from a *RC on PA for Children and Youth*. The RC was created by a research work group by conducting two systematic reviews to gather best published and nationally representative evidence. Policy documents, national surveys, and reports from government institutions were reviewed. The RC includes an assessment of *Government* as one of 14 PA indicators.
González et al., 2014 [110]	National (1 country)	A wide range of PA indicators for children and youth in Colombia	2005 – 2013	This article reported on the results from a *RC on PA for Children and Youth*. The RC was created by a research work group by reviewing current policies, national surveys, and other unpublished and published data. It includes an assessment of *Policy* as one of 12 PA indicators.
Green, 2009 [112]	National (1 country)	Sport policy priorities	n/a	The study analyses priorities in sport policy in the United Kingdom (mainly England). The conceptual framework of the study was based on the literature on governance and “new public management”.

Guo & Pan, 2016 [113] (in Chinese language)	National (1 country)	PA policies in the USA	1960 – 2014	The study used a text analysis to assess 40 USA PA policy documents. The authors searched through several databases and included formal and written government/non-government documents.
Halliday et al., 2013 [114]	National (1 country)	National PA Strategy in Scotland	n/a	This article reports on a review of the Scottish national PA strategy - *Let's Make Scotland More Active,* five years after its implementation.
Hämäläinen et al., 2016 [115]	International (6 countries)	HEPA policy-making	2012 – 2013 (interviews) 2001 – 2013 (documents)	The study explores cross-sector cooperation in HEPA policymaking in Denmark, England, Finland, Italy, the Netherlands, and Romania. Qualitative content analyses of 21 policies and of semi-structured interviews with 86 key policymakers were conducted. The study was a part of the project "REsearch into POlicy to enhance Physical Activity" (REPOPA 2011-2016).
Hämäläinen et al., 2016 [117]	International (4 countries)	Equality and equity in HEPA policies	2012 – 2013 (interviews) 2001 – 2013 (documents)	The study explores equality and equity in national and subnational PA promotion policies in Denmark[2], England, Finland, and Romania. Sixty-one interviews and 14 policies were analysed using a qualitative content analysis. The study was a part of the project "REsearch into POlicy to enhance Physical Activity" (REPOPA 2011-2016).
Hämäläinen et al., 2015 [116] Aro et al., 2016 [69]	International (6 countries)	Use of evidence in HEPA policies	2012 – 2013 (interviews) 2001 – 2013 (documents)	The study explored the use of evidence in national, regional, and local HEPA policies. Qualitative content analyses of 21 policies and of semi-structured interviews with 86 key policymakers were conducted. The study was a part of the project "REsearch into POlicy to enhance Physical Activity" REPOPA (2011-2016).
Harrington et al., 2016 [120, 121]	International (2 countries)	A wide range of PA indicators for children and youth in Northern Ireland and Republic of Ireland	2011 – 2015	This *RC on PA for Children and Youth* and the associated journal article were created by a research work group by evaluating academic articles and policy documents identified by online and database searches. It includes an assessment of *Government* as one of ten PA indicators.
Harrington et al., 2014 [118, 119]	International (2 countries)	A wide range of PA indicators for children and youth in Northern Ireland and Republic of Ireland	2003 – 2010	This *RC on PA for Children and Youth* and the associated journal article were created by a research work group by reviewing key data sources identified by online and database searches. It includes an assessment of *Government* as one of ten PA indicators.
Herrera-Cuenca et al. 2016 [122, 123]	National (1 country)	A wide range of PA indicators	n/a	This *RC on PA for Children and Youth* and the associated journal article were created by a research work group by reviewing literature that included: peer reviewed articles;

[2] For Denmark, only subnational (local and regional) policies were analysed. Therefore, findings for Denmark are not presented in Additional file 3.

		for children and youth in Venezuela		published national data reports; grey literature; national/local reports on PA; and public policy actions. It includes an assessment of *National Level Policies* as one of 13 PA indicators.
Huang et al., 2016 [124, 125]	National (1 country)	A wide range of PA indicators for children and youth in Hong Kong	n/a	This *RC on PA for Children and Youth* and the associated journal article were created by a research work group by searching three types of data sources: government reports; peer reviewed journal articles; and relevant national journals, and by collecting additional information through a manual search and personal contacts. It includes an assessment of *Government - Strategies, Policies, Investments* as one of nine PA indicators.
Jebb et al., 2013 [126]	National (1 country)	Obesity, actions and policy in England	Policies/actions 2003 – 2012	The article describes the actions taken and the most relevant information about the key strategy to tackle obesity in England and the impact of different obesity related policies. Summary and evaluation of the 28 national policy actions area presented.
Kahlmeier et al., 2015 [127]	International (37 countries)	PA recommendations	Until summer 2012 (only the most recent documents taken into account)	The study is an analysis and a systematic overview of national PA recommendations in European countries. It included documents in English, German, and French. Main information about the documents was obtained through a template completed by the WHO national information focal points. This was complemented by an online search. The study analysed whether national recommendations were in line with the WHO 2010 PA recommendations.
Kalman & Hamrik, 2013 [128] (in Czech language)	International (6 countries)	PA as public policy issue	Policies available in database from March to May 2010	The study is a content analysis of 25 national policy documents related to PA promotion in Finland, Great Britain, Ireland, the Netherlands, Norway, and Sweden. Only documents in English found through WHO's International Inventory of Documents on Physical Activity Promotion were included. Four hundred eleven text segments were coded. The study concluded that low PA levels are a public policy issue.
Kalman et al., 2008 [129]	National (1 country)	PA promotion in Czech Republic	n/a	This is a conference paper about PA promotion. It reviewed the community recreation management system and interventions to increase PA. Twelve documents on national, subnational and supra-national level were included in the analysis.
Katapally et al., [130, 131]	National (1 country)	A wide range of PA indicators for children and youth in India	2004 – 2016	This *RC on PA for Children and Youth* and the associated journal article were created by a research work group by reviewing: peer reviewed data sources; grey literature; and other nationally representative published and unpublished data. It includes an assessment of *Government - Strategies, Policies, and Investments* as one of ten indicators.
Katikireddi et al., 2011 [132]	National (1 country)	Analysis of the *Healthy Lives, Healthy People* white paper	n/a	The article evaluates Government's white paper *Healthy Lives, Healthy People*. It analyses whether English public health policy is evidence-based.
Katzmarzyk et al., 2016 [133, 134]	National (1 country)	A wide range of PA indicators	n/a	This *RC on PA for Children and Youth* and the associated journal article were created by a RC research advisory committee by reviewing key nationally-representative data

		for children and youth in USA		sources, mainly surveys. It includes an assessment of *Government Strategies and Investments* as one of ten PA indicators.
Kobayashi et al, 2017 [135]	National (1 country)	Sport policy in Vanuatu	n/a	This article outlines the development of national sport policy in Vanuatu. It analyses government objectives and policies for sport and the extent and nature of sport programmes supported by the government.
Koh, 2010 [136] (in Korean language)	International (5 countries)	PA recommendations and guidelines	Mainly from 1990s	The study reviewed PA guidelines/recommendations for adults from Australia, Canada, England, Japan, and the USA to inform the development of Korean national PA guidelines. PA guidelines/recommendations were identified in publications that were previously known to the author and by conducting a literature search through PubMed, Medline, and Google Scholar.
Kranzler et al., 2013 [137]	National (1 country)	National Programme to Promote Active, Healthy Lifestyle	n/a	The study analysed the Israel's *National Programme to Promote Active, Healthy Lifestyle* and its compliance with the *Health in All Policies* strategy for health governance.
Kruusamäe et al., 2016 [138]	National (1 country)	A wide range of PA indicators for children and youth in Estonia	2008 – 2016	This article reported on the results from a *RC on PA for Children and Youth*. The RC was created by a research work group by reviewing "original publications", key surveillance data, surveys, and database information. It includes an assessment of *Government - strategies, policies, and investments* as one of nine indicators.
Kudláček et al., 2012 [139] (in Czech language)	National (1 country)	National policies on infrastructure for leisure-time PA	n/a	The study assessed national, regional, and local level policies related to leisure-time PA. Thirteen structured qualitative interviews with policy makers and experts were conducted as well as focus groups with ten representatives of end users. Content analysis was performed on interviews, focus groups, and documents (legislations and regulations). The study was a part of project "Improving Leisure-time Physical Activity in the Local Arena" (IMPALA).
Lachat et al., 2013 [140]	International (83 countries)	Diet and PA policies – NCD prevention	1st January 2004 – 1st January 2013	This article is a systematic policy review. Structured content analysis of national health, NCDs, and nutrition policies for low- and middle-income countries was performed. Internet search of national ministries' websites and the WHO database was conducted. Data about PA policies were available for 35 countries.
Lagos et al., 2016 [141]	National (1 country)	*Choose to live healthy* programme	2010 – 2013 president's declaration	The study assessed *Choose to live healthy* programme in Chile using a discourse analysis. The analysis of 11 items included: national documents; legislation related to programme; four president's annual declarations to the Government; speech/presentation of the First Lady; national study *Healthy Chile*; and a leaflet published within the *Choose to live healthy* programme.
Larsen et al., 2016 [142] Larsen et al., 2017 [143]	National (1 country)	A wide range of PA indicators for children and	2007 – 2016	This *RC on PA for Children and Youth* and the associated journal article were created by a RC research committee by reviewing the best available research and policy strategies. It includes an assessment of *Government Strategies and Investments* as one of nine PA indicators.

		youth in Denmark		
Liu et al., 2016 [144]	National (1 country)	A wide range of PA indicators for children and youth in Shanghai and China	Literature search in September 2014	This article reported on the results from a *RC on PA for Children and Youth*. The RC was created by a research work group by reviewing literature in Chinese and English. It includes an assessment of *Government* as one of nine PA indicators. Surveys and Delphi method were used to inform some indicators.
Liukkonen et al., 2014 [145]<br>Gråstén et al., 2014 [226]	National (1 country)	A wide range of PA indicators for children and youth in Finland	n/a	This *RC on PA for Children and Youth* and the associated journal article were created by a research work group involving key experts and stakeholders. It includes an assessment of *Government – Strategies, Policies, Investments* as one of nine PA indicators. Five main data sources informed the indicator assessment process.
Lu & Henry, 2011 [146]	National (1 country)	Sport policy in China	1949 - 2008	The study is a historical review of sport policy development in China. The study is focused on rural areas.
Maddison et al., 2016 [149]<br>Maddison et al., 2015 [148]	National (1 country)	A wide range of PA indicators for children and youth in New Zealand	2014 – 2016	This *RC on PA for Children and Youth* and the associated journal article were created by an expert panel that updated the 2014 RC. The panel identified and reviewed available evidence and key data sources. Consultations with stakeholders were also undertaken. It includes an assessment of *Government Initiatives* as one of nine PA indicators.
Maddison et al., 2014 [147]	National (1 country)	A wide range of PA indicators for children and youth in New Zealand	2005 – 2013	This article reported on the results from a *RC on PA for Children and Youth*. The RC was created by an advisory group by mainly reviewing nationally-representative survey data. It includes an assessment of *Government Initiatives* as one of nine PA indicators.
Manyanga et al., 2016 [150, 151]	National (1 country)	A wide range of PA indicators for children and youth in Zimbabwe	2003 – 2015 (years of publication of included studies)	This *RC on PA for Children and Youth* and the associated journal article were created by a<br>RC work group by reviewing the best available published or unpublished literature that includes, for example: policy documents; graduate student theses; and syllabi. The RC includes an assessment of *Government Strategies and Investments* as one of ten PA indicators.
Matalas in Simonopoulos (ed.), 1997 [152]	National (1 country)	Programmes/policies on nutrition, fitness and “Sport for All”	n/a	This is a conference paper that presents an overview of sports, nutrition, fitness, education, and “Sport for All” policies and programmes in Greece from the Classical period until mid-1990s.
Méndez, 2015 [153] (in Spanish language)	National (1 country)	PA policy in Mexico	n/a	The study analyses PA policy through several government documents, mainly focusing on *General Law on Physical Culture and Sports* and its related programme. Design, entry into agenda and force, implementation, and evaluation of the PA policy are

				discussed.
Milton & Bauman, 2015 [40]	National (1 country)	PA policy in England	n/a	The study is a critical analysis of national PA policy cycles in England. Literature search was performed to identify relevant past and present PA documents. Scientific literature search through the PubMed database and web-based search through websites of the Department for Culture Media and Sport and the Department of Health were undertaken. Documents were analysed to identify content relevant to four key elements of PA policy.
Milton & Grix, 2015 [154]	National (1 country)	Public health policy and walking in England	Analysed year: 2008 (Interviews in 2012)	This case study analysed *policy window* related to walking promotion using the *Multiple Streams* framework. Semi-structured interviews with experts from relevant organisations in walking sector were conducted. Literature and electronic search were performed to obtain relevant policy documents. Data from literature and interviews were analysed using the inductive content analysis.
Mota et al., 2016 [155]	National (1 country)	A wide range of PA indicators for children and youth in Portugal	2010 – 2016	This article reported on the results from a *RC on PA for Children and Youth*. The RC was created by a committee that searched through available databases, official reports, and websites. It includes an assessment of *Government* as one of nine PA indicators.
Murphy & Waddington, 1998 [156]	National (1 country)	"Sport for All" policy in Great Britain	n/a	The study examines the objective to improve population's health outlined in "Sport for All" programmes and focuses on key social differences between PA and sport. Two case studies from Great Britain are presented.
Musingarimi, 2009 [158]<br>Musingarimi, 2008 [157]	National (4 constituting countries)	Obesity policies	1992 -2008 (documents from that period)	The study reviewed and comparatively analysed PA and nutrition policies related to obesity in four constituting countries of the United Kingdom. Twenty-two policy documents were identified through an Internet-based search and a literature review. The search was complemented by 15 semi-structured interviews with key informants such as policy makers, public health specialists, academics, and advocates from trade/voluntary organisations.
Nardo et al., 2016 [159]	National (1 country)	A wide range of PA indicators for children and youth in Brazil	n/a	This article reported on the results from a *RC on PA for Children and Youth*. The RC was created by a research work group of experts and stakeholders by reviewing several sources of national data and published peer-reviewed publications. It includes and assessment of *Government Strategies and Investments* as one of nine PA indicators.
Nishtar et al., 2006 [160]	National (1 country)	NCD Prevention Action Plan in Pakistan	n/a	The article describes the development process, components, and content of the *National Action Plan on Noncommunicable Disease Prevention, Control, and Health Promotion* in Pakistan.
Nyström et al., 2016 [161, 162]	National (1 country)	A wide range of PA indicators for children and	2005 – 2015	This *RC on PA for Children and Youth* and the associated journal article were created by a research work group by assessing relevant research studies and policy documents in Swedish and English. It includes and assessment of *Government Strategies and Investments* as one of nine PA indicators. Two additional indicators

		youth in Sweden		related to diet and obesity were included in the RC.
Ocansey et al., 2016 [164]	National (1 country)	A wide range of PA indicators for children and youth in Ghana	1975 – 2015 (only for peer reviewed literature, not other documents)	This article reported on the results from a *RC on PA for Children and Youth*. The RC was created by experts from ministries, NGOs, and higher education institutions. They reviewed data related to PA in children and youth from the following sources: peer-reviewed literature; published and unpublished theses/dissertations; and school physical education/sports syllabi and documents. The RC includes an assessment of *Government – Strategies, Policies, Investments* as one of ten PA indicators.
Ocansey et al., 2014 [163]	National (1 country)	A wide range of PA indicators for children and youth in Ghana	1975 – 2013	This article reported on the results from a *RC on PA for Children and Youth*. The RC was created by a team of researchers by reviewing available documents and research. It includes an assessment of *Government – Strategies, Policies, Investments* as one of nine PA indicators.
Oja & Titze, 2011 [234]	Global overview	PA recommendations	n/a	The article provides an overview of PA recommendations around the World. Content of the WHO recommendations, American College of Sports Medicine and the American Heart Association recommendations, and the US national recommendations are presented.
Onywera et al. 2016 [165, 166]	National (1 country)	A wide range of PA indicators for children and youth in Kenya	n/a	This article reported on the results from a *RC on PA for Children and Youth*. The RC was created by a work group by reviewing information from the following data sources: peer-reviewed literature; unpublished graduate student theses; presentations at peer-attended fora; and data from relevant organisations and agencies. It includes an assessment of *Government and Nongovernment* (*Strategies, Policies, Investments)* as one of nine PA indicators.
Pate et al., 2011 [37]	International (10 countries)	PA policies in children and youth	n/a	In the study, a review of government and non-government policy documents in Chinese and English that promote PA in children and youth was conducted. Documents were searched through databases and websites of, for example, national public health institutions, the WHO, and health ministries.
Pérez-Escamilla, 2016 [167]	National (1 country)	National dietary and PA guidelines in Mexico	n/a	The article describes the development process and the content of the document the *Mexican Dietary and Physical Activity Guidelines.*
Piggin & Heart, 2017 [169]	National (1 country)	PA advocacy and policy in the United Kingdom	2012 – 2014 (media reports analysis)	The study analyses how PA in the United Kingdom has been framed as key policy issue using the *Multiple Stream*s framework. *Meso*, *micro,* and *macro* parts of the agenda setting were assessed. The analysis included: government statements; media reports; the policy change initiative *Tackling Physical Inactivity: A Co-ordinated Approach*; publicity material from policy communities and interest groups; and social media interactions. This was combined with participant observation.
Piggin, 2008 [168]	National (1 country)	Sport and recreation	from January 2002 to	This doctoral dissertation discusses “dissemination and challenge” of policies written by *Sport and Recreation New Zealand.* Data were gathered through several sources

		policy in New Zealand	December 2007	including: policy documents; media articulations of policy; public debate over policy; and two semi-structured interviews with key informants. Data collection techniques included: observations; interviews; media and policy analysis; and reflexive journal. Thesis was guided by a critical discourse analysis.
Pilar Rodriguez et al., 2012 [170]	National (1 country)	A wide range of PA indicators for children and youth in Mexico	2009 – 2011	This *RC on PA for Children and Youth* was created by a panel of experts by reviewing: government reports; peer-reviewed literature; websites with relevant data or information; and state and federal programmes and laws. It includes an assessment of *Policy and programmes* as one of six PA indicators.
Pratt et al., 2016 [171]	International (4 countries)	PA research and policy	n/a	The study reported on the interplay between PA policy and research using four case studies from Australia, Brazil, Mexico, and the United Kingdom.
Prévot-Ledrich et al., 2016 [172] (in French language)	National (1 country)	HEPA policies	November 2014 – January 2016	The study reviewed HEPA public policies in France by using the second version of HEPA PAT. Data were collected through a document search and by conducting 15 interviews with experts. Data were validated and discussed at a one-day workshop.
Prista et al., 2016 [173]	National (1 country)	A wide range of PA indicators for children and youth in Mozambique	2014 – 2016	This article reported on results from a *RC on PA for Children and Youth*. The RC was created by the *Research Group for Physical Activity and Health* by reviewing relevant websites, reports, and databases. The research group used questionnaire and conducted interviews to obtain additional data. The RC includes an assessment of *Government* as one of nine PA indicators.
Prista et al., 2014 [174]	National (1 country)	A wide range of PA indicators for children and Youth in Mozambique	n/a	This article reported on results from a *RC on PA for Children and Youth*. The RC was created by the *Research Group for Physical Activity and Health* by reviewing reports, databases, and websites. Due to a lack of information, the research group distributed questionnaire among group members and established direct contacts with NGOs and government institutions. The RC includes an assessment of *Policy* as one of nine PA indicators.
Ramadan et al., 2010 [175]	National (1 country)	National PA plan in Kuwait	n/a	This article describes principles and background behind the development of the *National Physical Activity Plan* for Kuwait.
Ramirez Varela et al., 2017 [176] Ramirez Varela et al., 2016 [32]	International (217 countries)	PA surveillance, policy, and research	up to 2013	This almanac and the associated journal article contain RCs on PA for 217 countries. Data were obtained through Internet search. Data for 139 countries were reviewed and approved by country contacts. The RCs reported on the availability of national or sub-national PA plans, which was one of six PA indicators.
Reddy et al., 2007 [177]	National (1 country)	A wide range of PA indicators for children and youth in South Africa	1999 – 2007	This *RC on PA, Nutrition and Tobacco use for Children and Youth* was created by a scientific advisory panel by reviewing available data sources that were peer-reviewed and published or already presented in a peer-reviewed forum. Some data were drawn from unpublished studies which had peer-reviewed study designs. It includes an assessment of *Legislation: Sport and Education* as one of the indicators related to PA promotion.

Reilly et al., 2016 [180, 181]	National (1 country)	A wide range of PA indicators for children and youth in Scotland	2013 – 2016	This *RC on PA for Children and Youth* and the associated journal article were created by a research work group by reviewing available evidence. Consultations with a stakeholder group were undertaken to provide additional information and feedback. The RC includes an assessment of *National Policies, Strategies and Investment* as one of ten PA indicators.
Reilly et al., 2014 [179]<br>Reilly et al., 2013 [178]	National (1 country)	A wide range of PA indicators for children and youth in Scotland	n/a	This *RC on PA for Children and Youth* and the associated journal article were created by a research work group by reviewing available evidence. Formal online consultations with stakeholders were undertaken to provide additional information and feedback. The RC includes an assessment of *National Policies, Strategies and Investment* as one of ten PA indicators.
Rodriguez Martinez et al., 2014 [182, 183]	National (1 country)	A wide range of PA indicators for children and youth in Mexico	2010 – 2013	This *RC on PA for Children and Youth* and the associated journal article were created by a Mexican RC work group by reviewing literature in Spanish and English. The RC includes an assessment of *Government* as one of nine PA indicators. Grades for indicators were assigned by consensus during a meeting with members of AHK Canada RC team.
Roman-Viñas et al., 2016 [184]	National (1 country)	A wide range of PA indicators for children and youth in Spain	2006 – 2015	This article reported on the results from a *RC on PA for Children and Youth*. The RC was created by a research work group. The group's Chair conducted a narrative review and gathered key information. The RC includes an assessment of *Government* as one of nine PA indicators.
Rütten et al., 2013 [185]	International (8 countries)	Supportive environments for leisure-time PA	n/a	The study analysed supportive environments for PA, community action and policies in eight EU member states. It provides a comparative analysis of the following countries: Czech Republic, Denmark, Finland, France, Germany, Lithuania, Portugal, and Spain. The study was based on a secondary analysis of Eurobarometer data and the data collected as part of the EU funded research project entitled *Improving Leisure-time Physical Activity in the Local Arena* (IMPALA). In the IMPALA project data were collected by semi-structured interviews with key informants, focus groups, and document analysis.
Salinas & Fio, 2003 [186]<br>(in Spanish language)	National (1 country)	Health and PA policies in Chile	n/a	The study analyses PA as part of Chilean health promotion policy. It describes and analyses several key strategies related to PA promotion.
Schranz et al., 2016 [190, 191]	National (1 country)	A wide range of PA indicators for children and youth in Australia	2014 – 2016	This *RC on PA for Children and Youth* and the associated journal article were created by a research work group by reviewing the best available national- and state-based PA data. It includes an assessment of *Government Strategies and Investments* as one of 12 PA indicators.

Schranz et al., 2014 [188, 189]	National (1 country)	A wide range of PA indicators for children and youth in Australia	2008 – 2014	This *RC on PA for Children and Youth* and the associated journal article were created by a research work group by reviewing the best available national- and state-based PA data. One state-based survey and a number of national surveys were used as data sources. The RC includes an assessment of *Government – Strategies, Policies, Investments* as one of 12 PA indicators.
Sember et al., 2016 [192]	National (1 country)	A wide range of PA indicators for children and youth in Slovenia	2005 – 2015	This article reported on the results from a *RC on PA for Children and Youth*. The RC was created by a research work group by reviewing: data compiled from national databases; research findings published in peer-reviewed journals; and government initiatives. The RC includes an assessment of *Government – Strategies, Policies, Investments* as one of nine PA indicators.
Seppälä et al., 2017 [39]	National (1 country)	Policy recommendations related to PA, nutrition and SB in Finland	Most recent document, search in September 2016	The study is a "behaviour change wheel" guided content analysis of six key national policy documents related to worksites. Policy recommendations targeting employees' PA, SB, and nutrition were coded. A systematic search of the websites of ministries and research institutes operating under ministries was conducted.
Sharif et al., 2016 [193, 194]	National (1 country)	A wide range of PA indicators for children and youth in Malaysia	2009 onward	This *RC on PA for Children and Youth* and the associated journal article were created by a research work group by reviewing key data sources. An external expert validated the proposed grades for PA indicators and supporting evidence. The RC includes an assessment of *Government Strategies and Investments* as one of 11 PA indicators. The *Annual Report of Ministry of Health* (2012) was the key data source that informed the assessment of this indicator.
Skille & Sobakken, 2011 [195]	National (1 country)	Sport (policy) and its relation to health in Norway	n/a	The study analyses how health is treated within sport policy in Norway. Historical books were examined to retrieve information about sport policy before 1973. Six contemporary policy documents were also analysed. On the general level, the analysis is hermeneutic.
Smith et al., 2016 [196]	National (1 country)	Sport, PA, public mental health policies in England	1995 – 2016	This article is a critical overview of PA, community sport, and public mental health policy. Eighteen key policy documents published from 1995 to May 2016 in England were analysed.
Standage et al., 2014 [197]	National (1 country)	A wide range of PA indicators for children and youth in England	n/a	This article reported on the results from a *RC on PA for Children and Youth*. The RC was created by a research work group, a Scientific Officer, and a Chief Scientific Officer. The Scientific Officer identified key articles and gathered available evidence. The RC includes an assessment of *Government Strategies and Investments* as one of nine PA indicators.
Stratton et al., 2014 [199]	National (1 country)	A wide range of PA indicators	2007 – 2014	This *RC on PA for Children and Youth* was created by an expert group by reviewing relevant data. The group discussed development of the RC at five meetings. Delphi approach was used to achieve grading consensus for eight PA indicators. The RC

		for children and youth in Wales		includes an assessment of *National Policy, Strategy, and Investment* as one of the indicators. The assessment of the indicator was informed by seven key policy documents.
Stuji & Stokvis, 2015 [200]	National (1 country)	Sport and PA policy in the Netherlands	Since 1950s (documents)	This case study analyses Dutch PA/sport-related policy documents published by national sport organisations and the government using a historical sociological perspective.
Tammelin et al., 2016 [201, 202]	National (1 country)	A wide range of PA indicators for children and youth in Finland	"most current evidence"	This *RC on PA for Children and Youth* and the associated journal article were created by a work group by reviewing eight key data sources. The RC includes an assessment of *Government Strategies, Policies, and Investments* as one nine PA indicators.
Tan, 2015 [203]	National (1 country)	National fitness policy in China	Until 2015	The study analyses changes in China's national fitness policy using five policy change indicators. It employs the analytical framework of elite theory. Twenty-five semi structured interviews with officials from national governing sports bodies and six interviews with sport academics and journalists were conducted. Media publications and government policy documents were analysed.
Tanaka et al., 2016 [204, 205]	National (1 country)	A wide range of PA indicators for children and youth in Japan	n/a	This *RC on PA for Children and Youth* and the associated journal article were created by a research work group by reviewing relevant data. The RC includes an assessment of *Government Strategies and Investments* as one of 11 PA indicators. National laws, strategies, policies, and ordinances were reviewed to inform the assignment of the grade to this indicator.
Tremblay et al., 2016 [34]	International (38 countries)	Global matrix of grades related to PA in children and youth	RCs from 2015 and 2016	This publication contains consolidated findings of RCs on PA in children and youth. RCs include an assessment of a wide range of PA indicators for children and youth, including the indicator *Government Strategies and Investments*. Nine common indicators from 38 countries are presented. Data collection methods, quality and quantity of data, and available evidence varied across the countries. Each indicator was graded by the group of experts in charge of their country's RC.
Tremblay et al., 2014 [33]	International (15 countries)	Global matrix of grades related to PA in children and youth	RCs from 2013 and 2014	This publication contains consolidated findings of RCs on PA in children and youth. The RCs include an assessment of wide range of PA indicators for children and youth, including the indicator Government Strategies and Investments. Nine common indicators from 15 countries are presented. Data collection method, quality and quantity of data, and available evidence varied across the countries. Each indicator was graded by the group of experts in charge of their country's RC.
Tremblay et al., 2011 [206]	National (1 country)	PA guidelines in Canada	n/a	The study describes the development process and the content of the Canadian PA guidelines for children, youth, adults, and older adults.
Tyler et al., 2016 [207]<br>Stratton et al, 2016 [198]	National (1 country)	A wide range of PA indicators for children and youth in Wales	2013 – 2015 ("the most recent at the time")	This *RC on PA for Children and Youth* and the associated journal article were created by a research work group by reviewing relevant data sources. The RC includes an assessment of *National Government Policy, Strategies and Investments* as one of ten

				PA indicators. The grade for this indicator was informed by: strategy documents; policy documents; and other publically available guidance.
Uys et al., 2016 [208]	National (1 country)	A wide range of PA indicators for children and youth in South Africa	2011 - 2016	This article reported on the results from a *RC on PA and Nutrition for Children and Youth.* The RC was created by a work group by reviewing dissertations, peer-reviewed manuscripts, grey literature, government reports, and websites. The RC includes –PA- and nutrition-related indicators. It includes an assessment of *Government—Strategies, Policies*, *Investments* as one of ten PA indicators.
Vallgårda, 2015 [209]	International (4 countries)	Obesity policies	2008 - 2011	The study analyses national plans for reducing the prevalence of obesity using the "concept of problematisation". Policies from England, France, Germany, and Scotland were compared.
Van Mechelen in Simonopoulos (ed.), 1997 [210]	International (12 countries)	National policies for promotion of PA, physical fitness and nutrition	n/a	This conference paper and presents an overview of the availability of national PA/physical fitness and nutrition policies in Europe. The article also reports on nationwide PA initiatives. Data were gathered by a network of formal and informal experts.
Vuori et al., 2004 [10]	National (1 country)	PA policy development in Finland	1974 – 2004	The article presents an overview of policies related to PA and sport in Finland. The full policy development over 30 years and policy and programme evaluation are described.
Vuori et al., 1998 [211]	National (1 country)	Influence od national PA programmes on local level in Finland	n/a	The study describes the PA policy development in 1990s and two national PA promotion programmes in Finland – *the Finland on the Move* and the *Fit for Life*. It reviews their purpose and implementation, their evaluation, and the types of projects within these two programmes.
Wachira et al., 2014 [212, 213]	National (1 country)	A wide range of PA indicators for children and youth in Kenya	n/a	This *RC on PA for Children and Youth* and the associated journal article were created by a stakeholder and reviewer group. The RC was informed by two systematic literature reviews and the following sources: data from government organisations and practitioner communities; graduate student theses; and the *International Study of Childhood Obesity, Lifestyle and Environment*. It includes an assessment of *Government and Nongovernment* (*Strategies, Policies, Investments)* as one of ten PA indicators.
Wijtzes et al., 2016 [214, 215]	National (1 country)	A wide range of PA indicators for children and youth in Belgium	n/a	This *RC on PA for Children and Youth* and the associated journal article were created by a research work group by reviewing relevant data. The RC includes an assessment of *Government Strategies and Investments* as one of 9 PA indicators. Data sources to inform this indicator included policy documents and websites on federal and local regulations/rules. Two additionally assessed indicators were related to dietary behaviours and weight status.

Wilkie et al., 2016 [216, 217]	National (1 country)	A wide range of PA indicators for children and youth in England	2013 – 2016	This *RC on PA for Children and Youth* and the associated journal article were created by an expert panel by reviewing: several national surveys; government reports; reports from the Office for Standards in Education, Children's Services and Skills; and regional reports and datasets from other organisations. The RC includes an assessment of *Government Strategies and Investments* as one of nine PA indicators.
Woods & Mutrie, 2012 [218]	National (2 constituting countries)	PA policy	n/a	The article outlined a rationale for PA promotion and the decrease of physical inactivity from a public health perspective. It presents two case studies in relation to PA policy from Ireland and Scotland.
World Health Organization, 2015 [233]	International (28 countries)	A wide range of HEPA indicators	n/a	This publication contains country factsheets related to HEPA promotion in the EU. It is an overview of information related to monitoring, surveillance, and policy response. The WHO/EU PA focal points provided and validated PA-related data for their countries. For each member state a "country profile" was created from the data collected using a questionnaire on 23 HEPA indicators.
World Health Organization, 2014 [232]	International (22 countries)	PA promotion in Eastern Mediterranean region	September – December 2013	This publication reports on PA promotion in 22 countries of the Eastern Mediterranean region. Information about policies was collected through HEPA PAT completed by each country's focal point. The focal points were selected individuals from: relevant ministries; the WHO country office; or academic institutions. Twelve countries submitted their responses.
World Health Organization, 2010 [228]	International (27 countries)	Prevention of obesity, unhealthy nutrition, and insufficient PA	Data were collected during first six months of 2009	This is a report of a meeting of the network of the National Information Focal Points. The report is a part of a joint WHO/European Commission monitoring project on obesity. One of the project's seven work packages dealt with *National policies and actions*. The WHO national focal points collected data on public policies and policy documents on diet, nutrition, and physical activity, and also data on public-private partnerships and voluntary actions by economic actors.
World Health Organization, 2010 [229]	International (WHO European Region and EU)	Prevention of obesity, unhealthy nutrition and insufficient PA	n/a	This publication is a report from a meeting of representatives of the National Information Focal Points from the EU member states and the representatives of the WHO Nutrition Counterparts from the WHO European Region member states. The representatives reviewed and discussed the progress made in obesity prevention, improvement of PA and nutrition, and implementing policy action. Detailed reports are given for six countries; namely Germany, Hungary, Macedonia FYR, Poland, Switzerland, and the United Kingdom.
World Health Organization, 2010 [230]	International (10 countries)	Health inequity in children and adolescents	n/a	This publication is a summary of results from the WHO/Health Behaviour in School-aged Children Forum. It contains case studies from ten countries related to socio-environmentally determined health inequities. Six case studies include information about national PA policies for Armenia, England, Germany, Ireland, Norway, and Poland.

World Health Organization, 2010 [12]	International (27 EU member states)	PA promotion policy development and legislation	1993 – 2010 (only the most recent documents taken into account)	This publication is a review/report on PA policy development and legislation in EU member states. One hundred thirty-nine included documents were identified by searching for relevant key words and on the websites of: health promotion agencies; national ministries; subnational and local initiatives; and other activities and projects aiming at PA promotion. The search was complemented by a questionnaire sent to the WHO Nutrition Counterparts and by country-specific templates completed by National Information Focal Points.
World Health Organization, 2007 [227]	International (48 - WHO European region)	Policy development related to nutrition, PA, and obesity prevention	n/a	This publication is a report on policy developments related to nutrition, PA, and obesity. It is a product of the review of the information obtained from: national policy documents in English; publications; reports of the WHO meetings; websites of national public health institutions; databases; and websites of ministries of environment, health, and transport.
Wu, 2014 [219]	National (1 country)	Network governance of *Active Canada 20/20*	n/a (oldest document analysed – 1987)	This master thesis explores the movement of *Active Canada 20/20: A Physical Activity Strategy and Change Agenda for Canada.* This case study used "network governance" as its theoretical framework. The policy and document analyses were complemented with 12 semi-structured interviews and one direct observation.
Xu et al., 2014 [220] (in Chinese language)	International (2 countries)	PA in *Healthy People*[3] programme	1979 - 2010	The study assessed the content, development, objectives, and implementation of the USA *Healthy People* programme. The last section of the paper provided a comparison of the Programme to *Health China 2020* policy and recommendations for China.
Yoonkyung et al., 2016 [221, 222]	National (1 country)	A wide range of PA indicators for children and youth in South Korea	2011 – 2015	This *RC on PA for Children and Youth* and the associated journal article were created by a research work group by conducting a systematic literature review of peer reviewed articles and government reports in English and Korean. National surveys were preferred data sources. The RC includes an assessment of *Government Strategies and Investment* as one of ten PA indicators.
Zaabi et al., 2016 [223, 224]	National (1 country)	A wide range of PA indicators for children and youth in United Arab Emirates	1998 – 2014 (dates of published data sources used)	This *RC on PA for Children and Youth* and the associated journal article were created by a research work group, a Chief Scientific Officer, and a Scientific Officer. The officers searched and reviewed the available evidence. The RC includes an assessment of *Government Strategies and Investments* as one of nine PA indicators.
Zembura et al., 2016 [225]	National (1 country)	A wide range of PA indicators for children and youth in Poland	2013 – 2015 (data collection)	This article reported on the results from a *RC on PA for Children and Youth*. The RC was created by a research work group by reviewing key data sources. It includes an assessment of *Government Strategies and Investments* as one of nine PA indicators.

[3] A wrong translation of the programme name (*Healthy Citizen* instead of *Healthy People*) was used in the paper.

AHK = Active Healthy Kids; EU = European Union; HALO = Healthy Active Living and Obesity Research Group; HEPA = health-enhancing physical activity; HEPA PAT = Health-enhancing physical activity policy audit tool; NCD = Noncommunicable disease; NGO = Nongovernmental organisation; PA = physical activity; RC = report card; SB = sedentary behaviour; USA = United States of America; WHO = World Health Organization

Full text of the articles available in English, if not noted otherwise.
