# Supplementary material for: A global systematic scoping review of studies analysing indicators, development, and content of national-level physical activity and sedentary behaviour policies
Source: Int J Behav Nutr Phys Act. 2018 Nov 28;15:123. doi: 10.1186/s12966-018-0742-9 (PMC6263060; doi:10.1186/s12966-018-0742-9)
Supplement: Supplementary file 3 — Summary results of studies analysing indicators, development, and content of national-level physical activity and sedentary behaviour policies: country-specific findings. (PDF 836 kb) [file 12966_2018_742_MOESM3_ESM.pdf]

Additional file 3 – Summary results of studies analysing indicators, development, and content of national-level physical activity and sedentary behaviour policies: country-specific findings

Country / Study	Summary national-level findings
Albania	
Kahlmeier et al., 2015 [127]	- National PA recommendations have not yet been developed.
World Health Organization, 2010 [229]	- PA is integrated in an umbrella public health strategy: *Albanian health reform project. Towards a healthy country with healthy people – public health and health promotion strategy 2002-2010*.
American Samoa	
Ramirez Varela et al., 2016 [32]	- NCD plan includes PA (details are not specified).
Andorra	
Kahlmeier et al., 2015 [127]	- National PA recommendations have not yet been developed.
Angola	
Ramirez Varela et al., 2016 [32]	- No national/subnational PA plan.
Antigua and Barbuda	
Ramirez Varela et al., 2016 [32]	- PA is mentioned in the document entitled *Strategic Plan for the Prevention and Control of Non-communicable diseases for countries of the Caribbean Community 2011-2015*.
Argentina	
Ramirez Varela et al., 2016 [32]	- A national or subnational PA plan is available (details are not specified).
Armenia	
World Health Organization, 2010 [229]	- Policy documents in the PA area are not yet available.
World Health Organization, 2010 [230]	- Obligatory requirements related to PE classes and standard of sport facilities are mentioned in the law on sport for children and adolescents.<br>- The *National strategy on child and adolescent health 2009 – 2015* was approved in 2009.<br>- Promotion of sport among young people was established by the National Olympic Committee and the President of Armenia.<br>- NGO-led initiative received funds from the Government to raise public awareness related to healthy lifestyles.
Aruba	
Ramirez Varela et al., 2016 [32]	- PA is mentioned in the document entitled *National Plan Aruba 2009-2018 for fight against overweight and obesity related health issues*.
Australia	
Coenen et al., 2017 [38]	- *Australia's physical activity and sedentary behaviour guidelines* (2011) recommend concrete measures for reduction of SB, such as breaking up long periods of sitting or walking to a colleague instead of calling or emailing them.<br>- *Officewise - A Guide to Health and Safety in the Office* (Government, 2008) states that various tasks should involve a change in "involve a change in posture and muscles used to perform the work".<br>- More detailed suggestions how to reduce SB have been proposed in the factsheets published by the Government – *Benefits for movement – Be upstanding*, *Strategies to help you stand up, sit less move more* (2014), *The business case for reducing sedentary work* (2015).
Pratt et al., 2016 [171]	- National Heart Foundation of Australia is considered the central promoter of policy reform related to PA.<br>- Department of Infrastructure and Transport issued an urban policy that focuses on promotion of active transport (2011).<br>- Multisector coalitions have had impact on Australian active living policy.
Ramirez Varela et al., 2016 [32]	- No national/subnational PA plan.

Schranz et al., 2016 [190, 191]	- The assigned grade for the indicator *Government – Strategies, Policies, Investments* from *RC on PA for Children and Youth* is D.<br>- Major concern is that the Australian Government has not established an overarching strategy or a national PA plan.<br>- Recommendations on the minimum amount of PA and the maximum duration of uninterrupted SB are included in the *Australian PA and SB Guidelines.*<br>- Government withdrew main positive initiatives, since the RC from 2014 was released
Schranz et al., 2014 [188] [189]	- The assigned grade for the indicator *Government Strategies and Investments* from *RC on PA for Children and Youth* is C+.<br>- Government introduced major initiatives such as *National Partnership Agreement on Preventative Health*, *Healthy Children*, and *Active After-School Communities.*<br>- Establishment and implementation of coherent national PA policy and increased awareness of the PA guidelines is needed.
Bellew et al., 2011 [8]	- Examples of evidence based policy actions for PA promotion (2009-2010) related to social marketing and mass media setting: the *Measure up* campaign the *National PA and healthy eating campaign,* and inter sectoral *Premiers PA Task Force Strategic Plan* (2007-11). Several other campaigns such as *Find thirty everyday,* mass media campaign (2nd phase 2009-11).
Brown et al., 2011 [43]	- National policy document: *Be Active Australia draft National Physical Activity for Health Action Plan* was published by the National Public Health Partnership in 2004.
Ceccarelli et al., 2011 [90]	- The identified goals in policies that deal with nutrition, PA, and obesity (details not specified) are general and not quantifiable and measurable.<br>- Australia adopted the *National Chronic Disease Strategy*.
Pate et al., 2011 [37]	- The data were extracted from the documents by the National Public Health Partnership - Be *Active Australia: Draft National Physical Activity for Health Action Plan* (2004), *Be Active Australia: A Framework for Health Sector Action for Physical Activity, 2005–2010* (2005).<br>- PA policy measures were found in the following areas: a) health education (e.g. enable training for healthcare professionals in schools in motivational interviewing techniques related to PA); b) community environmental support (e.g. create parks, playgrounds, and open spaces that are interesting and challenging for youth and children); c) school environmental support (e.g. encourage schools to conduct fitness tests of their students annually and store the collected data); d) active transport/urban design (e.g. organise a designated car drop off zone half a kilometre from schools and support it by organised walking to school from the zone); e) mass media/advertising campaigns (e.g. use celebrities with positive image to become spokespersons for PA promotion).
Koh, 2010 [136]<br>(in Korean language)	- In 1999, the Department of Health and Ageing of the Australian Government issued the *National Physical Activity Guidelines for Australians*. They contained 4 recommendations: movement should be considered as opportunity, not inconvenience; every day be active in as many ways as possible; put together a minimum of 30 minutes of moderate PA on most, preferably all days a week; engage in regular vigorous PA for extra fitness and health benefits.
Bornstein et al., 2009 [79]	- Target groups specified in the comprehensive national PA plan *Be Active Australia: A Framework for Health Sector Action for Physical Activity 2005-2010* are: aging Australians; populations with special needs; children; Aboriginal Australians; and disadvantaged adults.
Bellew et al., 2008 [6]	- Australian PA policy was reviewed against nine (HARDWIRED) criteria. The “Active through multi-strategic, multi-level partnerships”, “highly consultative in development” and “widely communicated” indicators are partially achieved. Partial progress is seen in “developed in stand-alone and synergistic policy modes”, “evidence-informed and evidence-generating”, and “role-clarified and performance-delineated” indicators. The only substantially achieved criteria is “defined national guidelines for HEPA”. There is no progress regarding “independent evaluation” and little progress regarding whether policy is “resourced adequately”.
Bellew in Bull et al., 2004 [74]	- *Developing an Active Australia: a framework for action for physical activity and health* (launched by the Federal Health Minister in 1998) – first response of the health sector on *Active Australia.*<br>- Active Australia Alliance issued a draft of a national plan (2000-2003), but it has never been ratified. The Alliance was supposed to ensure a coordinated approach at

	the national level, but it foundered.
Bull et al., 2004 [26]<br>Schöppe et al., 2004 [187]<br>Bull et al. in Bull et al., 2004 [86]	- *Active Australia* (launched in 1996) emphasizes PA as a priority area at all governmental levels. It was developed after consultations of experts and state/territory agencies and it uses multiple strategies related to environments, education, evidence, and infrastructure. It operates through a cooperation between all governmental levels and uses intersectoral approach formalised by the Active Australia Alliance. In order to provide coordinated national action, Active Australia is linked to other national strategies and receives federal financial support (total amount not documented). Its identity is recognised through media campaigns and a logo. One of its results was the formation of the Strategic Inter-Government forum on PA and Health (SIGPAH).
Fullagar, 2003 [105]	- First phase of the *Active Australia* was extended from 1997 until 2001. It aimed to build<br>partnerships between State and Federal government departments in recreation and health. The main goal was to promote benefits of PA among the entire population. Within campaign's discourse women are considered a sedentary population. It acknowledges the importance of ensuring infrastructure to support active living, but that seems to be of a lesser priority than modifying individual leisure behaviour.
Fullagar, 2002 [104]	- Before the Commonwealth Department of Tourism and Recreation had been established in 1972, discourse related to health and leisure was organised predominately around PE and fitness that had "distinct masculine- and military-related origins".<br>- *Life be it!* became nation-wide campaign in late 1970s. It emphasised improved life quality through active leisure. It produced an image of body that experiences leisure as a psychotherapeutic enterprise.<br>- *The Active Australia* emerged in late 1990s with national and state focus. Within it there is "a moral prescription" that PA is good and that inactivity is a result of an ineffective management of time.
Egger et al., 2001 [102]	- *National PA Guidelines for Australians* (1999) were developed in three major steps: establishing set of science-based guidelines acceptable for major stakeholders; assessing the guidelines in the general population to ensure they have potential for motivating inactive individuals to become more active and ensure they are easy to understand; providing the final assessment by Scientific Advisory Board to assure that their scientific validity was not compromised by changes made due to consumer feedback.
Austria	
Ramirez Varela et al., 2016 [32]	- The PA plan entitled *National Action Plan for Physical Activity (Nationaler Aktionsplan Bewegung)* is available.
Kahlmeier et al., 2015 [127]	- The national PA recommendations were published in the document entitled *Austrian recommendations for health-promoting physical activity*, issued in 2010.<br>- Specific recommendations are provided for children/young people, adults, and older adults.<br>- The PA recommendations for adults are fully aligned with the WHO recommendations.<br>- The document includes recommendations on muscle-strengthening activities for adults and older adults and recommendations on SB for children/young people.
Ceccarelli et al., 2011 [90]	- The identified goals in policies that deal with nutrition, PA, and obesity (details not specified) are general and not quantifiable and measurable. Perspective to reach objective is ten years.
World Health Organization, 2010 [12]	- The following national documents related to PA were identified: *Fit for Austria Charter* (2007); *Cycling master plan: strategy for the promotion of cycling traffic in Austria* (2006); *Austrian strategy for sustainable development* (2002); and *Principle of health education* (1997).<br>- Since 2009, there has been a coordinating mechanism in the area of PA promotion, and the leasing institution has been the Ministry of Sport. The participating stakeholders are government departments on sport, health, and education.
World Health Organization, 2007 [227]	- The Fund for Healthy Austria and Austrian Organisation for Sport initiated projects related to nutrition and PA (in 2006) under umbrella concept *Fit for Austria*.
Van Mechelen in Simonopoulos (ed.), 1997 [210]	- There are no national policies for the promotion of PA or physical fitness. National walking programme is identified as one of the nationwide activities for the promotion of physically active lifestyle.

Azerbaijan	
Kahlmeier et al., 2015 [127]	- National PA recommendations have not yet been developed.
World Health Organization, 2010 [229]	- Policy documents in the PA area are not available.
Bahrain	
Ramirez Varela et al., 2016 [32]	- The PA plan entitled *National Strategy for Nutrition and Physical Activity 2014* is available (MOH, Undersecretary for Primary Care).
World Health Organization, 2014 [232]	- There are national NCD strategic plans or policies that include goals focused on PA (details not specified).<br>- There are NCD committees that address and coordinate PA.<br>- The WHO global recommendations on PA are formally adopted.<br>- Besides the general population, the population groups covered in national policy documents are: early years children; children and young people; older adults; workforce/employees; women; people with disabilities; people with chronic disease; sedentary/the most inactive; and families.<br>- Settings covered by national policy documents: kindergarten; primary schools; high schools; colleges/universities; primary health care; clinical health care; workplace; senior/older adult services; sport and leisure; and urban design/planning.
Bangladesh	
Ramirez Varela et al., 2016 [32]	- A national or subnational PA plan is available (details are not specified).
Barbados	
Ramirez Varela et al., 2016 [32]	- PA is mentioned in the document entitled *Barbados strategic plan for the prevention and control of Non-Communicable Diseases 2015-2019*.
Belarus	
World Health Organization, 2007 [227]	- *Health of the People*, a national-level programme implemented between 1999 and 2005,covered areas such as health and lifestyle, health and environment, labour and health.<br>- In 2003, the Council of Ministers approved the *Concept of the development of public health for 2003–2007*.
Belgium	
Coenen et al., 2017 [38]	- The *Flemish consensus statement regarding balanced diet and physical activity* (type of document classified as guidelines), published by the Ministry of Welfare, Public Health and Family (2012), mentions reducing SB.
Ramirez Varela et al., 2016 [32]	- The PA plan entitled *The Flemish Draft Action Plan on Nutrition and Physical Activity* is available.
Wijtzes et al., 2016 [214] [215]	- The assigned grade for the indicator *Government Strategies and Investments* from *RC on PA for Children and Youth* is C+.<br>- The *Flemish Action plan for nutrition and physical activity 2009-2015* (2008) has the goal to enhance healthy nutrition and PA among the general population and includes many strategies, priorities, and actions. One of the strategies is to provide good quality and accurate information to health care professionals related to PA, nutrition, and SB.<br>- Obligatory requirement of at least two hours of PE per week for children and youth has been a part of Flemish national legislation.
Kahlmeier et al., 2015 [127]	- The national PA recommendations were published in the document entitled the *National Food and Health Plan, Physical activity in Belgium: scientific consensus paper,* issued in 2007.<br>- Specific recommendations are provided for children/young people and adults.<br>- The PA recommendations are not fully aligned with the WHO recommendations.<br>- The document includes recommendations on muscle-strengthening activities for adults.<br>- The document does not include recommendations on SB.
Christiansen et al., 2014 [44]<br>World Health Organization, 2011 [231]	- There is a document related to sport promotion (only a subnational document available) entitled *Policy brief: 2009–2014 sport: through teamwork we score – towards a healthy, sustainable, results-oriented sports policy* [Beliedsnota: 2009–2014 Sport: Door samenspel scoren – Naar en gezond, duurzaam, resultaatgericht sportbelied] (2009).

World Health Organization, 2015 [233] [1]	- Target groups and policies vary across Flemish-, French-, and German-speaking regions.<br>- Belgium has not implemented a scheme for active travel to work.
Brown et al., 2011 [43]	- The national policy document: *Draft action plan on diet and physical activity 2008–2015* was published by the Ministry of Welfare Public Health and Family in 2008.
Ceccarelli et al., 2011 [90]	- The analysed policy document (details not specified) explicitly refers to the *WHO Global Strategy on Diet, Physical activity and Health* or to some other document assessing the problem of obesity.
World Health Organization, 2010 [228] [2]	- Belgium has decentralised health policy.<br>- Policy documents relevant for the promotion of PA in Belgium are mainly subnational.
World Health Organization, 2010 [12]	- *National plan on nutrition and health 2005–2010* was issued by the Federal Public Health Service for Health, Food Chain Safety and Environment in 2006 and falls within the public health sector.<br>- Since 2005, there has been a national coordinating mechanism for the promotion of PA, and the leading institution is the Federal Public Service of Public Health, Food, Safety and Environment. The participating stakeholders are government departments on sport, health, research, and education.<br>- Five subnational documents in public health, sport, and transport sectors are mentioned in this publication.
Van Mechelen in Simonopoulos (ed.), 1997 [210]	- There are no national policies for the promotion of PA or physical fitness. Youth sport campaign is identified as one of the nationwide activities for the promotion of physically active lifestyle.
Bermuda	
Ramirez Varela et al., 2016 [32]	- PA is mentioned in the document entitled *Strategic Plan of Action for the Prevention and Control of Non-communicable diseases for countries of the Caribbean Community 2011-2015.*
Bhutan	
Ramirez Varela et al., 2016 [32]	- A national or subnational PA plan is available (details are not specified).
Lachat et al., 2013 [140]	- In 2009, the Bhutan Ministry of Health issued the *National policy and strategic framework on prevention and control of non communicable diseases*.<br>- National policy includes the following targets and actions for PA promotion: national standards for PA and an Act that regulates built environment to uphold active living need to be established, put focus on sedentary and urban population, and support regular physical fitness and walking; level of PA among general population should be increased by improving the understanding about the link between PA and health; it should be advocated for increasing PA among the general population through the worksites; supportive environments should be created and education materials should be designed for supporting PA in children; Dratsang and the Ministry of Health will cooperate to incorporate training sessions and information on PA.<br>- Policy contained strategies for PA promotion focused on workplaces and specific strategies to address sedentary lifestyles.
Bolivia	
Ramirez Varela et al., 2016 [32]	- A national or subnational PA plan is available (details are not specified).
Bosnia and Herzegovina	
Kahlmeier et al., 2015 [127]	- National PA recommendations have not yet been developed.
Botswana	
Ramirez Varela et al., 2016 [32]	- No national/subnational PA plan.
Brazil	
Nardo et al., 2016 [159]	- The assigned grade for the indicator *Government Strategies and Investments* from *RC on PA for Children and Youth* is D.<br>- *National Plan of Body Practices and Physical Activity* aims to introduce public policies at the local level.<br>- Actions within *Growth Acceleration Program of Health 2007–2010* have been implemented. Main goals include: promote PA and the implementation and creation of

[1] Other findings from this study are not country-specific and are presented in the Additional file 4.
[2] Other findings from this study are not country-specific and are presented in the Additional file 4.

	projects in public recreational areas and schools; increase access to information related to healthier lifestyles; support investments in healthier urban spaces.<br>- Government strategies and programmes exist but their reach is limited (e.g. a key federal programme involved only 0.27% of Brazilian adolescents and children).
Pratt et al., 2016 [171]	- *New National Health Promotion Policy* (2006) listed PA among seven priority areas.<br>- The Ministry of Health initiated cooperation with research centres and universities from the USA and Brazil to better evaluate community PA programmes and provided financial support to local health departments for implementation of such programmes.
Ramirez Varela et al., 2016 [32]	- PA mentioned in the documents *Politica Nacional de promocao de Saude*, and *Plano de Enfrentamento de Doencas Cronicas Nao Transmissiveis.*
Gomez, 2015 [108]	- Since 1999, *National Policy of Nutrition* (*Política Nacional de Alimentação*), besides focusing on nutrition, also proposes increase in PA.<br>- In 2010, *Plano de Ações Estratégicas para o Enfrentamento das Doenças Crônicas Não Transmissíveis no Brasil*, was passed by the Congress and it established guidelines for anti-obesity initiatives for the next ten years. The plan includes a funding initiative entitled *National Policy and Health Promotion, Physical Activity, & Nutrition.*
Lachat et al., 2013 [140]	- In 2011, the Ministry of Health issued the *Strategic action plan to tackle noncommunicable diseases in Brazil: 2011–2022*.<br>- National policy included the following targets and actions for PA promotion: support PA in children on everyday basis; active aging should be promoted and elderly population. encouraged to participate in regular PA; support creation of healthy urban spaces; promote a healthy lifestyle and leisure PA for children and adolescents; promote PA at the population level; enhance PA levels during leisure time; establish education and communication campaigns for promotion of health through PA; create agreements with productive sectors to implement PA programmes.<br>- National policy targets for PA are proposed in the policy.<br>- The need to promote PA among elderly is also indicated in the policy.
Da Silva, 2007 [96]<br>(in Portuguese language)	- The end of 19th century was marked by understanding PA as a mean to maintain people fit and prepare them for a possible international conflict.<br>- In 1920, the War minister issued a regulation for obligatory PE for boys and girls older than 6 years in all private and public schools.<br>- From 1930s, soccer was linked with the national identity. This was especially strengthened in the 1950s, when the Government brought the World Cup to Brazil. The Government controlled popular music and media to portray sport and PA as good moral values. Radio broadcasts and periodicals praised the political regime and emphasised "sport should be at the service of the country".<br>- Federal Government exploited PA and sport to legitimise their actions especially during the Estado Novo regime.
Bull et al., 2004 [26]<br>Schöppe et al., 2004 [187]<br>Bull et al. in Bull et al., 2004 [86]	- *National Program for Physical Activity Promotion Agita Brazil* was established in 2000 and is being implemented by the Ministry of Health and funded by the Government. Its main goal is to promote health by increasing population's PA levels. It puts a special focus on people with NCDs. The programme is associated with the *Plan for Reogranisation of Blood Hypertension and Diabetes Mellitus Care* by the Federal Health Department.<br>- *National Food and Nutrition Policy* (Ministry of Health 1999) also mentions increased PA as one of goals.
Brunei Darussalam	
Ramirez Varela et al., 2016 [32]	- The PA plan entitled *National Physical Activity Guidelines 2011 BruMAP – NCD 2013-2014* is available.
Bulgaria	
Ramirez Varela et al., 2016 [32]	- PA is mentioned in the documents entitled *National for prevention of chronic non-communicable diseases 2014-2020* and *National Health Strategy 2014-2020.*
Christiansen et al., 2014 [44] World Health Organization, 2011 [231]	- *National strategy for the development of physical education and sports in the Republic of Bulgaria 2010–2020* (published in 2009) outlined that key objectives of the sport and PE system are to improve physical fitness and health of the population and increase the sporting image of Bulgaria, through creating conditions for systematic participation in sport and PE by all people.
World Health Organization, 2010 [12]	- The following national documents related to PA were identified: *National strategy for development of physical education and sport in Bulgaria 2010–2020* (2010); *National programme of sport for all* (2009); *National programme of sport for children in their free*

	time (2009); *National strategy and national programme for development of physical education and sports in Republic of Bulgaria, 2009–2013 (*2009); *National Strategy for Children 2008–2018* (2008); *National Health Strategy 2008– 2013* (2008); *National Programme for Environmental Health 2008–2013* (2008); *National programme for the development of school education and preschool training and preparation 2006–2015* (2008); *National Programme for Child Protection* (2006); *National Food and Nutrition Action Plan 2005–2010* (2005); *National strategy and national programme for development of physical education and sports in Republic of Bulgaria 2005–2008* (2005); *The Health Law* (initially published in 2004 and updated in 2008); and *Law for Physical Education and Sports* (initially published in 1996 and updated in 2008).<br>- Since 2003, there has been a coordinating mechanism in the area of PA promotion. The leading institutions have been the Science and State Agency of Youth and Sports, the Ministry of Health, and the Ministry of Education. The participating stakeholders are: government departments on education and research, sport, and health; NGOs; media; academia; private sector; and communities.
World Health Organization, 2007 [227]	- Several national programmes are available for PA promotion in children and youth such as *Education through Sport* (2004) and *Sport at School* (2006).
Cambodia	
Lachat et al., 2013 [140]	- In 2006, the Ministry of Health—Department of Preventive Medicine issued *National strategy for the prevention and control of noncommunicable disease: Cambodia, 2007–2010.*<br>- National policy includes the following targets and actions for PA promotion: modify school programme for promotion of women in sport and PA; increase public awareness of healthy lifestyles and the deficiency of PA as a risk factor, especially among women; create presentation materials on PA; engage media and sports personalities in PA promotion; create local strategies for PA promotion; develop suitable sport facilities for university students and school children; gather data on available public parks and bicycle ways.<br>- The policy includes concrete actions for the involvement of private sector in PA promotion.<br>- The policy targeted community at large.
Cameroon	
Clarke & Ojo, 2017 [93]	- The Ministry of Youth and Sports (1992) created the Ministry of Sports and Physical Education (2009) which has 5 departments: PE; high performance sport development; standards and sports organisations monitoring; studies, planning and cooperation division; general affairs.<br>- Sport is mainly led by the Government and it is an important aspect of the policy, understood as moral and physical imperative.<br>- PE is a part of core curriculum in schools and six key national standards are leading it: regular participation in PA; value PA for enjoyment, challenge, health, social interaction, and self-expression; maintain and achieve a health-enhancing fitness level; show responsible social and personal behaviour that respects others and yourself; demonstrate an understanding of movement principles, concepts, tactics, and strategies, as they apply to PA performance and learning.
Ramirez Varela et al., 2016 [32]	- No national/subnational PA plan.
Canada	
Coenen et al., 2017 [38]	- *Protect your back!* (2007) guideline by the Government, states that low back pain, upper back pain and neck pain can be the consequences of prolonged continuous daily sitting in front of computer screen or desk.<br>- *General Guide for Identifying Ergonomics-Related Hazards* (Government, 2011) states that remaining in the sitting posture for more than 6 hours/day is considered risky. This Guide also recommended avoiding prolonged standing, particularly on a hard surface.
Ramirez Varela et al., 2016 [32]	- PA plans entitled *Active Canada 20/20: A Physical Activity Strategy and Change Agenda for Canada; Canadian Sport Policy* and *Framework for Recreation in Canada* are available.
Active Healthy Kids (AHK) Canada, 2016 [58]<br>Barnes et al., 2016 [71]	- The assigned grade for the indicator *Strategies and Investments (Government)* from *RC on PA for Children and Youth* is B-.<br>- For the first time, *sleep* is included as an indicator in RC – Canada issued a *24 hour*

	Movement Guidelines for Children and Youth that emphasize relationship between PA, SB, and sleep.<br>- Sport Canada maintained annual contributions of around $16M towards enhancing sport opportunities for children and youth (in 2015-16).
AHK Canada, 2015 [57]	- The assigned grade for the indicator *Strategies & Investments (Government)* from *RC on PA for Children and Youth* is B-.<br>- The Federal Government increased PA funding compared to the last year.<br>- The Public Health Agency of Canada is collaborating with various private and non-government organisations to address physical inactivity within a collaborative project *Mobilizing Knowledge for Active Transportation.*
AHK Canada, 2014 [56]<br>Gray et al., 2014 [111]	- The assigned grade for the indicator *Government Strategies and Investments* from *RC on PA for Children and Youth* is C.<br>- *Canadian Sport Policy* was renewed.<br>- Less than one per cent of total health care budget is spent on PA, PE, sport, and health promotion.<br>- The Public Health Agency of Canada released *Preventing Chronic Disease Strategic Plan 2013-2016*
Wu, 2014 [219]	- *Active Canada 20/20: A Physical Activity Strategy and Change Agenda for Canada* (2012) – the strategy to promote mass PA was mainly influenced by the neoliberal ideology.<br>- Other important documents related to PA are: *Canadian Sport Policy* (2002, 2012); *Non Communicable Disease Prevention: Investments that Work for Physical Activity* (2011);<br>*Creating A Healthier Canada: Making Prevention A Priority* (2010); *Curbing Childhood Obesity: A Federal, Provincial and Territorial Framework for Action to Promote Healthy Weight* (2009); *The Integrated Pan-Canadian Healthy Living Strategy* (2005); *Pan-Canadian Physical Activity Strategy* (2004); *Physical Activity and Sport Act* (2003); and *National Recreation Framework* (1987).
AHK Canada, 2013 [55]	- The assigned grade for the indicator *Policy – Federal Government Strategies and Investments* from *RC on PA for Children and Youth* is C-.<br>- Federal government did not issue its own national PA plan.<br>- Gas tax fund is a good source of financial support for infrastructure related to active transport.<br>- $205,933,000 is the planned spending for *Sport Canada* (federal level, year 2012-13).
AHK Canada, 2012 [54]<br>Barnes et al., 2013 [72]	- Grades and findings for indicators from *RC on PA for Children and Youth under category Policy:*<br>1) *Federal Government Strategies:* assigned grade is D.<br>- *Active Transportation in Canada: A Resource and Planning guide* (released in 2011).<br>- No national strategy focused only on PA promotion<br>2) *Federal Government Investments:* assigned grade is F.<br>- Children Fitness Tax Credit – between $90M and $115M in annual federal tax revenue.
AHK Canada, 2011 [53]	- Grades and findings for indicators from *RC on PA for Children and Youth* under the category Policy.<br>1) *Federal Government Strategies*: assigned grade is C.<br>2*) Federal Government Investments:* assigned grade is F.<br>- Funding for PA promotion organisations (AHK Canada, Canadian Society for Exercise Physiology etc.) was reduced.<br>- Public Health Agency of Canada supported *Canadian Sedentary Behaviour Guidelines for School Aged Children and Youth* (2011) issued by the Canadian Society for Exercise Physiology, Healthy Active Living and Obesity research Group – the 1st evidence-based and systematic recommendations of the kind in the World.
Bellew et al., 2011 [8]	- *ParticipACTION* was established in 1971 and relaunched in 2007. It is a “national voice” for sport and PA participation.
Brown et al., 2011 [43]	- There is a national policy document entitled *The Toronto Charter for physical activity: A global call for action* (2010) created by the Global Advocacy Council for Physical Activity.
Ceccarelli et al., 2011 [90]	- In a policy document (details not specified), indirect and direct costs of chronic diseases related to weight and the cost related to physical inactivity are presented.<br>- The following specific goal for PA is stated in the policy document: by 2015, increase

	the proportion of people who participate in regular, 30 minutes per day of MVPA, by 20%.
Craig, 2011 [95]	- Canada has a multisectoral, multilevel approach in PA policy.<br>- The document *Physical inactivity: a framework for action; towards healthy, active living for Canadians* is a common framework for all the levels of the government that guides the development of strategies for PA promotion. It was developed by the Federal-Provincial/Territorial Advisory Committee on Fitness and Recreation in 1997.
Tremblay et al., 2011 [206]	- New Canadian PA Guidelines were developed in 2011 and provide specific recommendations for four age groups: children (5-11 y.o.), youth (12-17 y.o.), adults (18-64), older adults (65 years and over). They were developed by the Canadian Society for Exercise Physiology, *ParticipACTION*, and other stakeholders. The Government's Public Health Agency of Canada provided support.
AHK Canada, 2010 [52]	- Grades and findings for indicators from *RC on PA for Children and Youth* under the category *Policy:*<br>1) *Federal Government Strategies:* assigned grade is C+.<br>- PA component of the Healthy Living Strategy should be supported with an implementation plan.<br>*2) Federal Government Investments:* assigned grade is F.
Koh, 2010 [136]<br>(in Korean language)	*- Canada's physical activity guide to healthy active living* was issued in 1998 by the Public Health Agency of Canada. It was developed through the process of scientific review by various experts and in cooperation with relevant agencies and organisations.
AHK Canada, 2009 [51]	- The assigned grade for the indicator *Federal Government Strategies and Investments* from *RC on PA for Children and Youth* is C.<br>- Federal budget for 2009 included additional $500M to build and renew recreation and sport facilities in the next two years.<br>- Children's Fitness Tax Credit has been transformed to meet the need of families with lower income.<br>- Bilateral provincial and federal funding announcements were made, especially directed at the facilitation of *Sport Canada's Policy on Sport for Persons with a Disability* and at Aboriginal population initiatives, but implementation process of the initiatives is unclear.<br>- It is recommended to establish national PA Policy coordinated by the Government.
AHK Canada, 2008 [50]	- The assigned grade for the indicator under category *Policy - Progress and Government Strategies and Investments* from *RC on PA for Children and Youth* is C+.<br>- Federal budget announced $24M investment in supporting sport.
AHK Canada, 2007 [49]	- The assigned grade for the indicator under category *Policy - Progress and Government Strategies and Investments* from *RC on PA for Children and Youth* is C.<br>- *Sports Canada's Policy on Sport for Persons with a Disability* – encouragement of involvement in sport for people with disabilities with financial support of $12.5M.<br>- *Canada's Physical Activity Guides for Children and Youth* – inactive youth and children (6-14 y.o.) should increase level of PA by minimum 30 min/day and decrease screen time by at least 30 min/day.
Bergsgard et al., 2007 [76]	- *Fitness and Amateur Sport Act* (1961) emphasised importance of mass sport participation. However, around 1970, focus of the federal Government shifted towards elite sport neglecting support of general PA. It was mostly limited to the active lifestyle and promotion of individual fitness. An indication of change is visible in *The Canadian Sport Policy* (2002) through the treatment of "Sport for All", where the main objective was to increase the quality and level of active participation for all people.
AHK Canada, 2006 [48]	- The assigned grade for the indicator under category *Policy - Progress and Government Strategies and Investments* from *RC on PA for Children and Youth* is C -.<br>- Children Fitness tax credit announcement – non-refundable tax credit (max $500) for an enrolling child (under 16 y.o.) in PA programme (to begin in 2007).<br>- The Government announced commitment to spend at least one per cent of federal health funding per year on PA and amateur sport programmes for school children.
AHK Canada, 2005 [47]	- The assigned grade for the indicator under category *Policy - Federal Strategies and Investments* from *RC on PA for Children and Youth* is C-.<br>- *Physical Activity and Sport Act* was established.<br>- *Public Health Agency of Canada* was established.<br>- *Canada Physical Activity Guidelines* (1989) recommend working towards 90 additional minutes of MVPA for children and youth.

Bull et al., 2004 [26]<br>Schöppe et al., 2004 [187]<br>Bull et al. in Bull et al., 2004 [86]	- Sport Canada and Health Canada (Fitness and Active Living Program Unit) are the key institutions responsible for national PA policy, which is mentioned in the *Fitness and Amateur Sport Act* (1961).<br>- *ParticipACTION* (1971) is a fitness initiative, established by the Health Canada to promote PA for a healthy lifestyle.<br>- *Physical Inactivity: A Framework for Action – Towards Healthy, Active Living for Canadians* (1997) provides a base for (sub)national governments to determine priorities related to physical inactivity.
Bercovitz, 1998 [75]	- The Minister's Task Force on Federal Sport Policy published a report *Sport: The Way Ahead* in 1992. It provided a comprehensive analysis of sport in Canada and provided over hundred recommendations to enhance sport at all levels. The report outlined the need for shift within the sport system to incorporate a "Sport for All" focus. Four key themes dominated: create community-based and athlete-centred sport system; promote ethics, fair play, and values in sport; ensure accessible and equitable sport; and establish innovative and new strategic alliances and partnerships, particularly between Active Living and sport.
Bell-Altenstad & Vail, 1995 [73]	- When *Fitness and Amateur Sport Act* passed in 1961, federal government formally started to get involved with amateur sport.<br>- In 1986, Sport Canada published *Women in Sport* policy that addressed gender inequities in sport and outlined five key barriers affecting women's sport participation.
Cayman Islands	
Ramirez Varela et al., 2016 [32]	- PA is mentioned in the document entitled *Strategic Plan of Action for the Prevention and Control of Non-communicable diseases for countries of the Caribbean Community 2011-2015.*
Chile	
Aguilar-Farias et al., 2016 [60]	- The assigned grade for the indicator *Government – Strategies, Policies, Investments* from *RC on PA for Children and Youth* is C.<br>- No national PA recommendations or guidelines.<br>- *National Policy for Sports and Physical Activity* coordinated by the Ministry of Education, the Ministry of Sports, and the Ministry of Health to be launched in 2017.<br>- The Ministry of Sports was founded in 2013 and received an annual budget of 0.02% of the GDP. In 2015, it invested 0.16% of its annual budget in sport and PA for children and youth<br>- There is no indicator of the effectiveness of PA promotion programmes and policies. Written policies from different institutions are often overlapping or are in discrepancy.
Lagos et al., 2016 [141]	- The public programme *Choose to live healthy* (CHP) was transformed in 2013 into a Republic law (Ministry of Social Development) - *Ley 20.670*. It states that healthy diet, PA, family life, and outdoor activities can contribute to the prevention or reduction of the risk factors associated with NCDs. It also mentions that regular PA practices reduce the risk of NCDs such as cardiovascular disease, depression, breast or colon cancer, and diabetes type II, and that sport allows people to better control their weight and achieve energy balance.<br>- Due to high rates of obesity and sedentarism, the programme tries to solve those public issues by recommending PA and sport that are interpreted as "good movement" as opposed sedentarism which is "bad movement". Within the programme, sedentraism is presented as a danger that can make people become seriously ill.
Ramirez Varela et al., 2016 [32]	- PA is mentioned in the document entitled *Elige vivir sano, Programa dependiente del Ministerio de Desarrollo Social Estrategia Nacional de Salud para el cumplimiento de los Objectivos Sanitarios de la Decada 2011-2020.*
Bravo & Silva, 2014 [81]	- From 1920s to 1940s, sport was perceived as a vehicle for readiness for war. This link with military ended in 2001 with the *Law 19712*. The General Directorate for Sport and Recreation stopped being accountable to the Ministry of National Defence (sub-secretariat of War) and moved to the Ministry of General Secretariat of Government.<br>- One key provision of the Constitution has been responsible for order in the Chilean sport system – people have the right to freely organise themselves by also recognising the autonomy of the organisation.<br>- *Law 17276*, known as the law for the *Promotion of Sport* (1970) mentions promotion of professional and amateur sport activities, implementation and promotion of recreation programmes, administration of sporting facilities, and implementation and

	promotion of coaching education programmes.<br>- The division of Physical Activity and Sport is involved in the implementation of programmes and plans (as stated in *Sports Law*, that is *Ley del Deporte* 2001) and has four departments – high performance, mass sports, research and development, and sport organisations. The recreational unit of the Department of Mass Sport promotes active sport participation as a vehicle for social integration and focuses on target groups such as children, adults, and elderly.
Lachat et al., 2013 [140]	- In 2002, the Ministry of Health issued *Objetivos sanitarios para la decada 2000–2010*.<br>- National policy includes the following targets and actions for PA promotion: the promotion of PA at worksites; dissemination of the PA guidelines among the school and preschool children and in the general population; and the development of population guidelines.<br>- The policy included detailed actions and specified implementation plan for stakeholders.
Salinas & Fio, 2003 [186]<br>(in Spanish language)	- *National Plan for Health Promotion* was issued in 1999 by the Ministry of Health.<br>- PA is one of five key priority themes in this general health promotion policy. Policy is supervised by the *Vida Chile* Council. National targets related to PA and SB highlight the need to reduce the prevalence of SB (in the population aged 15 years or more) by seven per cent by 2010 and to rebuild public spaces to become suitable for healthy lifestyles.<br>- The Ministry of Health, the Ministry of Education, the National Sports Institute, and universities adapted the Canadian programme *Challenge for Active Living*, and pilot tested it to be applied in schools and in the community.
China	
Liu et al., 2016 [144]	- The assigned grade for the indicator *Government* from *RC on PA for Children and Youth* is D<br>- In 2007, the State Council of the P.R. China issued the document *Promoting physical activity and enhancing physical fitness of children and adolescents* (No. 7 Central Document).<br>- Low awareness of the policy (only 26.7% of parents were aware of it) was the key reason for the low grade.<br>- Since 2007, the Ministry of Education and the State Council have released funds and issued several policies to support PA promotion among children and youth.
Ramirez Varela et al., 2016 [32]	- The PA plan entitled *National regulations on fitness* is available.
Tan, 2015 [203]	- After the Bejing Olympic Games, a major shift happened in Chines sport (for all) policy and the idea to co-develop *sports for all* and elite sport occurred.<br>- State Council issued the *Regulation on National Fitness* (2009) that all governmental agencies under the State Council need to follow. It is outlined that, according to the law, people have the right to engage in *“Sport for All”* activities and local governments are obliged to have special budget for mass sport participation.<br>- The Government published the *White Paper – National Fitness Program Implementation* in 2011. General Administration of Sport outlined five key problems in the Programme: insufficient number of sport instructors who can often engage in mass sport promotion; insufficient number of fitness facilities as a key barrier for mass sport promotion; low proportion of people who regularly participate in sport, particularly among young children; and private and nongovernmental organisations playing limited role in sport promotion due to constraints by China’s administrative structure.
Xu et al., 2014 [220]<br>(in Chinese language)	- Unlike the US *Healthy People*[3] initiative, *Healthy China 2020* lacks an effective communication with public and external stakeholders. Its objectives are not specific enough to become feasible. It also lacks emphasis on PA and PA’s role in disease prevention. Furthermore, it lacks an effective channel for the on-line promotion and communication with public (e.g. it has no website).<br>- *Healthy China 2020* is the first version, so it should be continued and improved.
Lachat et al., 2013 [140]	- In 2012, the China Ministry of Health issued *China national plan for NCD prevention and treatment, 2012–2015*.<br>- National policy includes the following targets and actions for PA promotion: promote the working model for social sport and healthy lifestyle instructors by the communities;

[3] A wrong translation of the programme name (*Healthy Citizen* instead of *Healthy People*) was used in the paper.

	develop fitness and sports environment; enable secondary and primary students to engage in at least one hour of exercise per school day; increase the ratio of the people who exercise regularly to 32%; create healthy environment and promote regular exercise; enhance scientific guidance for mass sport; gradually enhance public sport facilities' utilisation and accessibility; and increase quality evaluation and monitoring for the environment.
Lu & Henry, 2011 [146]	- The period from 1949 until 1977 was characterised by Government's intention to improve people's fitness for labour and national defence. *The New Sports* ideology presented Government's guidelines and intention to develop sport in rural areas.<br>- In 1981, the directive entitled *Instruction on Caring about People's Cultural Life*, published by the Communist Party, focused on people's life quality and aimed to guide people's recreational activities to promote healthy culture.<br>- In 2004, policy *Year of Rural Sports* was published by the General Administration of Sports. It aimed to provide sport facilities, knowledge, and instructions in rural areas.
Pate et al., 2011 [37]	- Policy related to PE in schools states that: children should engage in at least two hours of PE a week; PE should be taught by highly qualified and certified teachers; and the PE curriculum should be included in the school review process.<br>- Policy related to community environmental support states that: public sports facilities and venues should be free or offered at discounts to students; parks, playgrounds, and open spaces that are interesting and challenging for youth and children should be created; community organisations and local authorities should be supported, funded, and encouraged to develop PA promotion programmes for families to get them active and use the existing infrastructure; and cooperation between colleges, youth clubs, and schools with community groups, local authorities, and health professionals to design programmes for maximising involvement in PA should be encouraged.<br>- Policy related to school environmental support states that: access to a broad range of activities such as dance, sports, active travel, play, exercise, and being active in daily tasks around and in school should be provided; schools should be encouraged to conduct fitness tests of their students annually and store the collected data; parks around schools and school playgrounds should be renovated and designed to inspire sport, movement, play, and outdoor education; award should be given to schools for promoting holistic health in the school setting, including the promotion of PA among students, parents, and staff.<br>- The data were extracted from the following documents: *Views of Strengthening Youth Sports and Enhancing Their Physical Fitness* (Central Committee of the Communist Party of China and State Council of China, 2007); *Sports Law of the People's Republic of China* (1995); and from the documents issued by the Ministry of Education of China and General Administration of Sports China - *Views of Further Strengthening School Sports and Enhancing Student's Physical Fitness* (2006); *Decision of Sunny Sports* (2006), *National Standards for Students' Fitness and Health* (2002, 2007); and *Notice of Popularizing the First National Primary and Secondary School Group Dance* (2007).
Chen in Simonopoulos (ed.), 1997 [91]	- "Sports for All" plan was in place since late 1995 and was integrated in the second chapter of the *Sports Law of the People's Republic of China* in order to develop mass sports activities. Key targets of the plan by the 2010 include: achieving a well-coordinated development of national economy, sports, and social affairs; completing a basic "Sports for All" system provided with distinctive Chinese features; improving the Chinese national health level and physique.<br>- Great importance will be given to the exercise for minorities, women, elderly, and people with disabilities, whilst the priority is put on youth and children.
Colombia	

González et al., 2016 [109]	- The assigned grade for the indicator *Government* from *RC on PA for Children and Youth* is B.<br>- National investment in sport and PA was 50% lower in 2016 than in 2014.<br>- PA is supported from a multisectoral perspective including sports, transport, education, social development, and health sector.<br>- *National Development Plan* (2014-2018) is new policy for social development and sports. PA programmes are seen as means that can help, in the post-conflict era, to rebuild social cohesion. It also outlines that the Ministries of Transport, Education, and Housing will create plans for promoting active transport to school.<br>- Through the strategy *Al colegio en bici*, bicycle loans to students are provided along with basic bicycle skills training and designing safe routes to school. *Muevete Escolar* promotes PA in schools through communication strategies, interventions, and education.<br>- Coldeportes (national body) runs the national programme *Healthy Habits and Lifestyle*, which offers regular PA sessions led by trained instructors in public spaces. Sessions are available for all ages. During 2015, it was implemented in six capital cities and 20 out of the 32 departments in Colombia.
Ramirez Varela et al., 2016 [32]	- The PA plan entitled *Programa Nacional de Actividad Fisica Colombia Activa y Saludable* is available.
González et al., 2014 [110]	- The assigned grade for the indicator *Policy* from *RC on PA for Children and Youth* is "4".<br>- There is a substantial number of policies for PA promotion (e.g. *Plan nacional de desarrollo 2010-2014*).<br>- There are PA promotion programmes with national coverage such as *Healthy Habits and Lifestyle Programme of Coldeportes* and the network of *Ciclovias-Recreativas.*<br>- In 2014, national investment in sport and PA was 174M USD.<br>- Current policies lack the evaluation of their effectiveness. There is still a large gap between concrete actions and written policies.
Costa Rica	
Ramirez Varela et al., 2016 [32]	- The PA plan entitled *National Plan for Physical Activity and Health 2011-2021* is available.
Lachat et al., 2013 [140]	- National policy (details not specified) includes the following targets and actions for PA promotion: the promotion of PA, recreation, and healthy lifestyles.
Croatia	
Ramirez Varela et al., 2016 [32]	- The PA plan entitled *The Republic of Croatia National Action Plan for Health Enhancing Physical Activity* is available.
Kahlmeier et al., 2015 [127]	- National PA recommendations are currently in the development process (the documents published until summer 2012 were reviewed).
World Health Organization, 2007 [227]	- *Food and nutrition action plan 2006–2010* has obesity prevention as an overall goal and focuses not only on nutrition but also on PA.<br>- *Croatian food and nutrition policy* developed in 1999 by the Ministry of Health and Social Welfare and the Croatian National Institute of Public Health contains objectives for the promotion of PA, healthy nutrition, and healthy lifestyles.
Cuba	
Ramirez Varela et al., 2016 [32]	- The PA plan entitled *Plan nacional de actividad fisica del Instituto Nacional de deportes, Educacion Fisica y Recreacion* is available.
Lachat et al., 2013 [140]	- In 2006, the Ministry of Public Health issued *Proyecciones de la salud pública en Cuba para el 2015*.<br>- National policy includes the following targets and actions for PA promotion: decrease SB prevalence to 32% in the population group older than 15 years; increase the percentage of adults engaged in PA to 40%; at worksite and at the population level, promote intersectoral participation in "systemic" PA.<br>- The policy contained specific strategies to address sedentary lifestyles.
Cyprus	
World Health Organization, 2010 [12]	- *National Nutrition Action Plan* issued in 2007 by the Ministry of Health mentions PA.<br>- Since 2005, there has been a coordinating mechanism in the area of PA promotion, and the leading institution has been the Ministry of Health. The participating stakeholders are: government departments on education and research, health, sport and food; academia; NGOs; media; and the private sector.

Czech Republic	
Ramirez Varela et al., 2016 [32]	- PA is mentioned in the document entitled *Health for all in 21st Century.*
Christiansen et al., 2014 [44] World Health Organization, 2011 [231]	- A document related to sport promotion is entitled *National programme for the development of sport for all* (2000). It mentioned the link between non-organised sport and natural environment (e.g. walking paths, bicycle lanes and cross-country skiing tracks) and emphasised sport facilities should be accessible for all people. It has no time frame and has a strong focus on supporting PA and sport for a broad population.<br>- Four ministries are responsible for the implementation of the strategy.<br>- Budget in the strategy for PA, sport, and healthy lifestyle is four million koruny (cca €164 629) over two years, but there is no overall budget or budget for other activities.
Rütten et al., 2013 [185]	- Public policies related to LTPA: *National Programme of sport development for all* (2000) and *National Cycling Strategy* (2004).<br>- Local, regional, and national budgets and money from lottery funds are granted for the promotion of voluntary sports organisations.<br>- Supportive environments for LTPA (indoor/outdoor sport facilities, infrastructure for LTPA) are broadly available. Urban and green spaces, usable for LTPA, are partially available.
Kudláček et al., 2012 [139]	- There is no comprehensive policy at the national level focused on LTPA infrastructure.<br>- *Sport Promotion Act* (*Zakon o podpore sportu* 115/2001, 219/2005) defines the position of sport in the society and different tasks for regional and local authorities. It mentions their responsibility for: creating conditions enabling sport participation; “Sport for All” development; and construction, maintenance, and reconstruction of sport facilities.<br>- *Government Resolution No. 17/2000 - the National Programme for the Development of Sport for All* (*Usnesení Vlády ČR č. 17/2000 k Národnímu programu rozvoje sportu pro všechny*) aims at transforming social and infrastructural conditions for lifelong healthy lifestyle promotion. It emphasised the need to provide opportunity for as many citizens as possible to engage in safe PA that does not harm the environment. This is the key document related to LTPA infrastructure and it is based on PA surveillance data for Czech Republic.<br>- *Government Resolution No. 673/2003 – Guidelines on the State Policy in Sport for the years 2004-2006* (*Usnesení Vlády ČR č. 673/2003 ke Směrům státní politiky ve sportu na léta 2004–2006*) mentioned the Ministry of Education, Youth and Sports as the responsible authority for sport (e.g. elite sport, sports events, school sport, “Sport for All”, sport infrastructure, science and research, athletes with disabilities, anti-doping programmes).<br>- *Government Resolution No. 678/2004 - National Strategy for the Development of Cycling (Usnesení Vlády ČR č. 678/2004 k Národní strategii rozvoje cyklistické dopravy České republic)* has four priorities focused on developing infrastructure and conditions to enhance cycling in Czech Republic.<br>- *State support for sport* (*Státní podpora sportu*) is a document published annually by the Sports Department of the Ministry of Education, Youth and Sports. It outlines financial investments in sport at the national level. Some programmes within the document are focused on the “Sport for All” development, school sport, development of sport for people with disabilities, and the support to the PA and sport infrastructure.
World Health Organization, 2010 [12]	- The following national documents related to PA were identified: *National cycling development strategy of the Czech Republic* (2005), *National Programme of Sport Development for All* (2000), and *Programme for Health Promotion* (2000).<br>- There is no coordinating mechanism in the area of PA promotion.
Kalman et al., 2008 [129]	- There are no national-level interventions for PA promotion.<br>- Government does not encourage people to be more active and does not recognise physical inactivity as a serious risk factor for NCDs.<br>- Creation of a strategy for PA promotion is proposed.
World Health Organization, 2007 [227]	- *National action plan against obesity* is to be developed by the National Council for Obesity and should be based on the *WHO global strategy on diet, physical activity and health.*<br>- In 2005, the Ministry of Transport issued a *National cycling development strategy.*

**Denmark**	
Larsen et al., 2017 [143]<br>Larsen et al., 2016 [142]	- The assigned grade for the indicator *Government Strategies and Investments* from *RC on PA for Children and Youth* is A-.<br>- The Danish Foundation for Culture and Sports Facilities, established in 1994, continuously supports leisure- and PA-related projects.<br>- *The Day-care Act* outlined "movement and body" as one of six key learning themes. *The Education Act* requires from schools to ensure that all children get at least 45 min of PA per day while at school.<br>- The need to establish more specific recommendations on reducing screen time and time spent in SB and raise awareness about the importance of that reduction was emphasised.
Hämäläinen et al., 2016 [115]	- HEPA policies cooperation and coordination processes and structures include: government/regional/local committees or working groups with cross-sector representatives; national/regional/local politically elected councils; intersectoral committees or working groups for HEPA; steering committees; administrative working groups including only public sector officers; formal consultation on HEPA policy for stakeholders; private sector involvement in policymaking; public hearings for citizens.<br>- There are no: field visits to make a policy; contacts between public sector officers responsible for HEPA between levels; scientific advisory groups/institutes/individuals; and established systems of policymaking.
Ramirez Varela et al., 2016 [32]	- PA is mentioned in the document entitled *Healthier life for all.*
Kahlmeier et al., 2015 [127]	- The national PA recommendations were published in the document entitled the *Physical activity guidelines,* issued by the Danish Health and Medicines Authority.<br>- Specific recommendations are provided for children/young people, adults, and older adults.<br>- The PA recommendations are not fully aligned with the WHO recommendations.<br>- The document includes recommendations on muscle-strengthening activities for adults and older adults.<br>- The document does not include recommendations on SB.
Rütten et al., 2013 [185]	- Public policies related to LTPA: *Healthy throughout Life 2002-2010*, *National Action Plan against obesity* (2003), *Danish Act of General Education* (1991), and *Act of General Education* (1991) that replaced *Leisure-time Act* (1969) which stated that municipalities are responsible for providing adequate infrastructure for youth/children premises and for covering expenses for organised PA and leisure time instructions for adults.<br>- Supportive environments for LTPA (indoor/outdoor sport facilities, infrastructure for LTPA, and urban/green space for LTPA) are broadly available.
Brown et al., 2011 [43]	- National policy document: *Healthy throughout Life – the targets and strategies for public health policy of the Government of Denmark, 2002–2010* was published by the Ministry of the Interior and Health in 2003.
Ceccarelli et al., 2011 [90]	- The analysed policy document (details not specified) explicitly refers to the *WHO Global Strategy on Diet, Physical activity and Health* or to some other document assessing the problem of obesity.<br>- The document includes a thorough analysis of PA and eating habits of the population.<br>- Stakeholders related to PA and sport mentioned in the policy document are sports goods manufacturers and advertising and recreation businesses.<br>- *Health, food and physical activity: Nordic Plan of Action on better health and quality of life through diet and physical activity* was issued by the Nordic Council of Ministers in 2006. The document established a number of common goals and the policy agenda for all Nordic countries, although individual countries have adopted, "partly different sets of actions within the designated areas of common priority".
World Health Organization, 2010 [12]	- The following national documents related to PA were identified: *Nordic Plan of Action on better health and quality of life through diet and physical activity* (2006), *National action plan against obesity – recommendations and perspectives* (2003), *Better health for children and young people* (2003), and *Healthy throughout life – the targets and strategies for public health policy of the Government of Denmark, 2002–2010* (2002).<br>- There is no coordinating mechanism in the area of PA promotion.
Daugbjerg et al., 2009 [11]	- Policy document *Healthy throughout Life 2002-2010* (2003) does not contain quantifiable PA goals and budget for policy implementation, but it does contain a

	requirement/intention for evaluation.
Branca et al., 2007 [80]	- Policy document *National action plan against obesity: recommendations and perspectives* (2003) proposed to develop a monitoring system for nutrition conditions, PA, body weight, and environmental determinants.<br>- Denmark's National Board of Health highlighted the need for transport policy that enhances PA opportunities in everyday life (e.g. car free cycling and pedestrian areas, providing bicycle parking connected with public transport and worksites).
World Health Organization, 2007 [227]	- *Health throughout Life 2002-2010*, a public health strategy, focuses on the major risk factors such as obesity, physical inactivity and unhealthy nutrition.<br>- 2007 was proclaimed by the Minister of Health and Internal Affairs as the year of *Denmark on the move* emphasising the importance of PA for all age groups.
Van Mechelen in Simonopoulos (ed.), 1997 [210]	- There are no national policies for the promotion of PA or physical fitness.<br>- The prevention of the ischemic heart disease is highlighted as one of the nationwide activities for the promotion of a physically active lifestyle.
Djibouti	
Lachat et al., 2013 [140]	- National policy (details not specified) includes the following targets and actions for PA promotion: build playgrounds; and promote PA.
Dominica	
Ramirez Varela et al., 2016 [32]	- PA is mentioned in the document entitled *Strategic Plan of Action for the Prevention and Control of Non-communicable diseases for countries of the Caribbean Community 2011-2015.*
Dominican Republic	
Ramirez Varela et al., 2016 [32]	- The PA plan entitled *Programa Nacional de Cultura Fisica y Deporte* (2014-2018) is available.
Ecuador	
Ramirez Varela et al., 2016 [32]	- PA is mentioned in the documents entitled *Plan Nacional "Buen Vivir" 2013-2017 / Ecuador Ejercicate.*
Egypt, Arab. Rep.	
Ramirez Varela et al., 2016 [32]	- No national/subnational PA plan.
World Health Organization, 2014 [232]	- Ministries of sport, youth and education have a leadership role regarding PA.<br>- Presence of legislation that mentions the requirements for the PE curriculum across different school grades was identified.
England	
Coenen et al., 2017 [38]	- In the publication *Start active, stay active - A report on physical activity for health from the four home countries' chief medical officers*, issued by the Government's Department of Health in 2011, it is stated that "all adults should minimise the amount of time spent being sedentary (sitting) for extended periods".
Piggin & Heart, 2017 [169]	- *Moving More, Living More: The Physical Activity Olympic and Paralympic Legacy for the Nation* (2014) has a goal to promote PA among the population and increase PA by engaging partners across all levels and sectors to work together.<br>- The main idea of the *Everybody Active, Every Day: An Evidence-based Approach to Physical Activity* (2014) is: "A plan to engage with many different sectors and employers to make the case for much more physical activity, every day".
Hämäläinen et al., 2016 [117]	- The document *Places People Play – delivering a mass participation sporting legacy from the 2012 Olympic and Paralympics Games 2011–2015* states goals such as: investment in regionally important multi-sport facilities; deploy and train 40,000 Sport Makers; create disability programme to inspire people with disabilities to participate in sport; and improve and protect playing fields across the country. The programme did not mention any equity issues except in some target groups (in respect to inequity between females and males).
Hämäläinen et al., 2016 [115]	- HEPA policies cooperation and coordination processes and structures include: government/regional/local committees or working groups with cross-sector representatives; scientific advisory groups/institutes/individuals; formal consultation on HEPA policy for stakeholders.<br>- There are no: national/regional/local politically elected councils; contacts between public sector officers responsible for HEPA between levels; established systems of policymaking; intersectoral committees or working groups for HEPA; steering

	committees; administrative working groups including only public-sector officers; private sector involvement in policymaking; field visits to make a policy; and public hearings for citizens.
Pratt et al., 2016 [171]	- Government's Department of Health refers to an independent organisation - National Institute for Health Care Excellence (NICE) - regarding certain area to receive guidance on prevention and health promotion or treatment of ill health. PA was one of the first areas to be addressed by NICE in 2006. The best available scientific evidence was applied by NICE to inform the development of the following guidance documents: *Four commonly used methods to increase physical activity* (2006) and *Physical activity and the environment* (2008).
Ramirez Varela et al., 2016 [32]	- The PA plan entitled *Everybody Active, Every Day 2014* is available.
Smith et al., 2016 [196]	- Only some national policy documents related to community sport and PA had some references on the importance of PA, and sport and exercise for public mental health), but were often very vague. The most explicit discussion related to sport/PA and benefits for public mental health was found in the documents *Sporting Future: a new strategy for an active nation* (2015) and *At Least Five a Week: Evidence on the Impact of Physical Activity and its Relationship to Health* (2004). The latter has a whole chapter dedicated to PA, mental illness, and psychological well-being in adults.<br>- None of the researched community sport/PA policy documents contained measurable, quantifiable goals and targets related to public mental health.
Wilkie et al., 2016 [216] [217]	- The indicator *Government Strategies and Investment* from *RC on PA for Children and Youth* did not receive a grade, it was marked as incomplete, because an independent evaluation of policies and strategies is lacking.<br>- No specific guidelines to address SB are available.
Kahlmeier et al., 2015 [127]	- The national PA recommendations were published in the document entitled *Physical Activity, Health Improvement and Protection. Start Active. Stay Active: A report on physical activity from the four home countries',* issued in 2011.<br>- Specific recommendations are provided for children/young people, adults, and older adults.<br>- The PA recommendations for children/young people and adults are fully aligned with the WHO recommendations.<br>- The document includes recommendations on muscle-strengthening activities for adults and older adults and recommendations on SB for children/young people, adults, and older adults.
Milton & Bauman, 2015 [40]	- The Department of Health and the Department for Culture Media and Sport are responsible for the promotion of sport and PA.<br>- *Game Plan* (2002) was the first England's policy document that mentioned quantifiable targets for PA prevalence. The document contained many references to PA and emphasised the importance of the promotion of mass participation in both PA and competitive sports.<br>- The *Strategy Statement on Physical Activity* (1996) by the Department of Health was informed by the Health Education Authority's symposium (1994) where over forty international and national experts reviewed the evidence to reach an agreement on what kind of messages should be promoted. The PA guidelines included in the document were endorsed in 2004 in the document entitled *At Least Five a Week* developed by the Chief Medical Officer.<br>- In case of PA recommendations, England showed a robust scientific, evidence based approach.
Milton & Grix, 2015 [154]	- *Olympic and Paralympic Games*, in London (2012) were the key driver that made government engage more in promotion of walking. In 2008, £7M were invested into walking promotion.<br>- Some of the national documents related to the promotion of walking: *Saving Lives: Our Healthier Nation* (1999); *Tackling Obesity in England* (2001); *Game Plan* (2002); *On the Move: By Foot* (2003); *At Least Five a Week* (2004); *Choosing Health—Making Healthy Choices Easier* (2004); *Walking and Cycling: An Action Plan* (2004); *The Future of Transport* (2004); *Choosing Activity—A Physical Activity Action Plan* (2005); *Making the Case: Improving Health through Transport* (2005); *Tackling Obesities—Future Choices* (2007); *Towards a Sustainable Transport System* (2007); *Delivering a Sustainable Transport System* (2008); *Before, During and After: Making the Most of the*

	London 2012 Games (2008); *Be Active, Be Healthy* (2009); *Active Travel Strategy* (2010); *Healthy Lives, Healthy People—Our Strategy for Public Health in England* (2010); *Start Active, Stay Active: A Report on Physical Activity from the Four Home Countries'* (2011); and *The Public Health Responsibility Deal* (2011).
Vallgårda, 2015 [209]	- In 2011, the document *Healthy Lives, Healthy People: A call to Action on Obesity in England* was published by the Department of Health. In the plan, individual's free choice is emphasised and a contradiction between Government's goal to ensure health and its goal to ensure freedom is present.
Christiansen et al., 2014 [44]<br>World Health Organization, 2011 [231]	- The following documents related to sport promotion were identified: *Play to win: a new era for sport* (2008) and the action plan *Sport England strategy 2008–2011* (2008). In this study, the two policy documents were referred to as a single strategy<br>- The strategy sets specific targets such as: to have two million more active people by 2012; to put coaching and competition in the focus of school sports system; and to offer five hours of PE/week to 5-16 y.o. children and adolescents.
Standage et al., 2014 [197]	- The indicator *Government Strategies and Investment* from *RC on PA for Children and Youth* did not receive a grade, it was marked incomplete, because of the unclear impact of policies on increased participation in PA.<br>- Number of policies relevant to adolescents' and children's health are in place, but only few are related to PA (e.g. *Giving all children a healthy start in life*, *Reducing obesity and improving diet*).
Jebb et al., 2013 [126]	- *Healthy weight, healthy lives: a cross-government strategy for England* was published by the Department of Health, Department of Children, Schools and Families, and the Cross-Government Obesity Unit in 2008. It was organised around the following five themes: healthy weight and growth for children; promotion of healthier diet; building PA into everyday lives – getting people to move as a regular part of their daily life; creating incentives for better health; personalised support and advice.<br>- The *Physical Activity Children's Play Strategy* was in place from 2008 until 2010 and its main aim was an investment of £235M to develop 30 new adventure playgrounds and 3,500 play spaces.<br>- *Start4Life* (2010) was a sub-brand of *Change4Life* strategy that targeted early years and pregnancy. Among other goals, it aimed to promote PA for infants.
Bellew et al., 2011 [8]	- Through the *Be Active Be Healthy* policy an initiative *Walk4life Miles* was developed by the Department of Health and Walk England.<br>- *Well@work* is a pilot programme for worksite health promotion.
Brown et al., 2011 [43]	- The following national policy documents were identified: *Healthier Communities: Improving health and reducing health inequalities through Sport* (Sport England, 2008), and *At least five a week- evidence on the impact of physical activity and its relationship to health* (Department of Health Physical Activity Health Improvement and Prevention, 2004).
Ceccarelli et al., 2011 [90]	- In the policy document *Tackling Obesities: Future Choices – Qualitative Modelling of Policy options* (2007), indirect and direct cost of chronic diseases related to weight and the cost related to physical inactivity are mentioned.<br>- In the policy document specific targets related to PA are set, such as: increase the level of participation in sport and PA by 2020, so 70% of population undertakes 30 minutes of PA five days a week; increase the number of school children who spend at least two hours per week doing school sport and high-quality PE, within and beyond the curriculum, to 75% by 2006 and to 85% by 2008 (compared to 25% in 2002); increase the number of people older than sixteen who participate in sport at least twelve times a year by 3%.
Katikireddi et al., 2011 [132]	- Many interventions in the white paper *Healthy Lives, Healthy People* are likely to be ineffective or there is a lack evidence showing their effectiveness.<br>- Eleven statements in the document relate to increasing PA among adults and children. Evidence was found for school based interventions related to walking promotion, for volunteer led walks (e.g. *Walk Once Week, Walking for Health*), and for interventions related to structural changes (e.g. *Healthy Towns*, *Cycle Demonstration Towns*).
Gillon, 2010 [107]	- *Sport England Strategy 2008-2011* sets specific targets such as: one million more people engaged in sport by 2012-13; improved system of the talent development in minimum 25 sports; 25% reduced post-16 decline in sports participation in at least five sports by 2012-13 %.

Koh, 2010 [136]<br>(in Korean language)	- The Department of Health's report from 2004 *At least five a week: evidence on the impact of physical activity and its relationship to health* stated that all adults should engage in at least medium intensity PA for at least 30 minutes on five or more days every week.
World Health Organization, 2010 [229]	- The campaign *Change4Life* was launched in 2009. It tackled a growing obesity problem and had a mission to encourage people to move more and eat well. It originally focused on children from five to 11 y.o., but it was extended to adults aged from 45 to 65 years and also early years children. An evaluation of the programme showed 99% of brand recognition.
World Health Organization, 2010 [230]	- The Government's Department for Children, Schools and Families set the goal to ensure that England is the best country for kids to grow up in. *The children's plan: one year on* and *The play strategy* were published in 2008. *The children's plan: building brighter futures* and *Aiming high for young people: a ten-year strategy for positive activities* were published in 2007.<br>- The Department of Health issued *Healthy weight, healthy lives: a cross-government strategy for England* in 2008.
World Health Organization, 2010 [12]	- The following national documents related to PA were identified: *Active Travel Strategy* (2010); *Cycling and Health: a Strategy for 2005–2008 and Action Plan for 2005–2006* (2006); *The Framework for sport in England* (2004); *The Future of Transport, a Network for 2030, White Paper* (2004); *Walking and Cycling: an Action Plan* (2004); *Travelling to school: an action plan, England* (2003); *Game Plan, strategy for delivering the governments sport and physical activity objectives* (2002); and *Tomorrow's roads: safer for everyone. The Government's road safety strategy and casualty reduction targets for 2010* (2000).
Bornstein et al., 2009 [79]	- *Be Active, Be Healthy: A plan for getting the nation moving* was published by the Department of Health. Timeline for achieving goals was not identified. Target groups included, for example, people with CVD, diabetics, people with mental illness, ageing adults, and people with mental/physical disabilities. Some of the quantifiable targets included two million more active adults by 2012, increasing average weekly PA duration by cca 5% over the baseline.
Green, 2009 [112]	- The document *A Sporting Future for All and Sport: Raising the Game* put special focus on PE and school sport and elite sport development. It was mentioned that sport provides unique contribution regarding the problem of social exclusion.<br>- In 2007, the Secretary of State for Culture Media and Sport made a claim about separation of *sport* from *physical activity.* Therefore, the Department for Culture, Media and Sport and Department of Health were in charge of developing the PA strategy for all, while Sport England kept its focus on sport participation and sport development.
Musingarimi, 2009 [158]<br>Musingarimi, 2008 [157]	- *Healthy Weight, Healthy Lives: A Cross Government Strategy for England* - the "obesity strategy" - mentions PA as one of the key goals.<br>- *Choosing activity: a physical activity action plan* (2005) aims to promote PA for all.
Daugbjerg et al., 2009 [11]	- Policy documents *Game Plan, Strategy for delivering the Government's sport and physical*<br>*activity objectives*, published in 2002, with the time frame until 2020, and *Choosing Activity—a Physical Activity Action Plan—Working in partnership with people, their communities, local government, voluntary agencies and business* (2005) contain quantifiable PA goals, budget for policy implementation, and a requirement/intention for evaluation.<br>- The document *The Future of Transport—a network for 2030—White Paper* (2004) does not contain quantifiable PA goals and a requirement/intention for evaluation but contains the budget for policy implementation.<br>- The document *Walking and Cycling—an action plan* (2004) contains the budget and a requirement/intention for evaluation, but it does not contain quantifiable PA goals and a time frame.<br>- The document *Tomorrow's roads: safer for everyone—the Government's road safety strategy and casualty reduction targets for 2010* (2000) does not contain quantifiable PA goals, budget and a requirement/intention for evaluation.
Bergsgard et al., 2007 [76]	- Publications *A Sporting Future for All: The Government's Plan for Sport* (2000) and *Game Plan*: *A Strategy for Delivering Government's Sport and Physical Activity Objectives* (2002) raised participation in sport higher on the national political agenda.<br>- Responsibility for "Sport for All" development lies at the national level and is split

	between the Sport England ("quasi-autonomous non-government organisation") and the Department of Culture, Media and Sport.
Branca et al., 2007 [80]	- In 2005, the Department of Health issued *Choosing activity: a physical activity action plan* that sets detailed goals for schoolchildren and priority groups (e.g. minority and ethnic groups, people with mental or physical disabilities, women, and people with low socioeconomic status).<br>- *Travelling to school: an action plan* mentions measures for schools, local governments, and national governments to promote more cycling and walking to and from school.
World Health Organization, 2007 [227]	- *Choosing activity: a physical activity action plan* was published in 2005 by the Department of Health. It includes the Government's plan to coordinate and encourage the action of various organisations and departments to promote increased participation in PA. Some of the mentioned actions include: PA and sport in schools; use of green spaces and transport plans; local actions to increase activity through sport; and enhancing PA levels through the use of pedometers (as advised by the National Health Service).
Cavill et al, 2006 [89]	- PA promotion as topic came latter on the agenda. In 1990s, no official government publication existed on PA benefits. That started to change in 2004 when *Report of the Chief Medical Officer in England* was published (Department of Health).
Murphy & Waddington, 1998 [156]	- Health benefits associated with PA were mentioned in the document *The health of the nation: a strategy for health in England* (1992), issued by the Department of Health and in the document *Young people and physical activity: promoting better practice* (1997), issued by Health Education Authority.<br>- *Sport: Raising the Game* (1995) issued by the Department of National Heritage showed the lack of coordinated action between government departments.
Van Mechelen in Simonopoulos (ed.), 1997 [210]	- There is a national policy for the promotion of PA entitled *Strategy Statement on Physical Activity.* Its main aim is to increase participation in moderate PA in everyday life.
Estonia	
Kruusamäe et al., 2016 [138]	**-** The assigned grade for the indicator *Government - strategies, policies, and investments* from *RC on PA for Children and Youth* is C.<br>- *The General Principles of the Estonian Sports Policy until 2030*, approved by the Government in 2015 includes the key aims and developmental directions for lifelong PA.<br>- Cooperation between ministries and cross-sectorial network for PA promotion should be improved.
Ramirez Varela et al., 2016 [32]	- PA is mentioned in the document entitled *National Health Plan 2009-2020.*
Kahlmeier et al., 2015 [127]	- The national PA recommendations were published in the document entitled *Estonian Nutrition and Food Recommendations*. The document was issued by the National Institute for Health Development in 2006.
Christiansen et al., 2014 [44]<br>World Health Organization, 2011 [231]	- The following document related to sport promotion was identified: *Strategic development plan for sport for all 2006–2010* [Liikumisharrastuse strateegiline arengukava 2006–2010]. The document was published in 2006. It emphasised that "Sport for All" should not be understood as a by-product of professional or elite sport, but circumstances have to be shaped so that every citizen can participate in sport. It also highlighted that sport facilities need to be within a short distance and mentioned it is important to address their quality, maintenance, lighting, and safety.
Ceccarelli et al., 2011 [90]	- *The Estonian National Strategy for Prevention of Cardiovascular Diseases 2005-2020* is focused on the following five strategic areas: PA; smoking; health care; nutrition; and securing local capacity and dissemination of information.<br>- The policy document includes a thorough analysis of PA and eating habits of the population.<br>- Specific quantifiable goals reported in the policy document include: by 2010, increase the percentage of people who undertake regular exercise to 45% of the population; and in the period between 2006 and 2010 increase the number of people who are involved in "Sport for All" by 20%.
World Health Organization, 2010 [12]	- The following national policy documents related to PA were identified: *National Health Plan 2009-2020* (2008); *Transport development plan 2006-2013* (2006); *Strategic development plan Sport for All 2006 – 2010* (2006); *Sport Act* (2005); *National strategy*

	for prevention of cardiovascular diseases 2005–2020 (2005); *National traffic safety strategy 2003–2015* (2003); *Estonian Sports Charter* (2002); and *Public Health Act* (1995).<br>- Since 2006, there has been a coordinating mechanism in the area of PA promotion, and the leading institution has been the Ministry of Culture. The participating stakeholders are: government departments on development, health, sport, and education and research; academia; media; communities; NGOs; and the private sector.
World Health Organization, 2007 [227]	- National strategy for the prevention of cardiovascular diseases was introduced in 2005 and includes four priority areas: PA; non-smoking; nutrition; and community development.<br>- The Ministry of Culture prepared the *Estonian Strategic Development Plan, Sport for All 2006–2010* in 2006. It promotes the growth of the *"Sport for All"* movement among the general population, promotes PA to make it more available to everyone, and aims to increase the number of facilities for regular training as well as other opportunities for regular training.
Faroe Islands	
Ramirez Varela et al., 2016 [32]	- PA is mentioned in the document entitled *Annual campaigns by the Board of Public Health.*
Ceccarelli et al., 2011 [90]	- *Health, food and physical activity: Nordic Plan of Action on better health and quality of life through diet and physical activity* was issued by the Nordic Council of Ministers in 2006. The document established a number of common goals and the policy agenda for all Nordic countries, although individual countries have adopted "partly different sets of actions within the designated areas of common priority".
Fiji	
Ramirez Varela et al., 2016 [32]	- The PA plan entitled *Fiji Plan of Action on Physical Activity* is in the consultation phase with stakeholders.
Finland	
Seppälä et al., 2017 [39]	- National policy documents that contain PA/SB recommendations are: the *National strategy for the reduction of sedentary behaviour* (2015); *Principles of good occupational healthcare practice guide* (2014); the *National strategy for physical activity promoting health and well-being 2020* (2013); and the *Action plan of the National Obesity Programme 2012–2015* (2013).
Hämäläinen et al., 2016 [117]	- The policy document *Development of health enhancing physical activity and nutrition 2008–2011* emphasised the importance of decreasing health inequality differences between population groups.<br>- The policy document *Promotion of physical activity 2009–2012* emphasised the importance of providing equal opportunities for active living and PA.<br>- The *Strategy for Walking and Cycling 2011–2020* mentioned equity as "an economically advantageous choice for individual citizens".
Hämäläinen et al., 2016 [115]	- HEPA policies cooperation and coordination processes and structures include: government/regional/local committees or working groups with cross-sector representatives; national/regional/local politically elected councils; contacts between public sector officers responsible for HEPA between levels; scientific advisory groups/institutes/individuals; intersectoral committees or working groups for HEPA; formal consultations on HEPA policy for stakeholders; public hearings for citizens.<br>- There are no: field visits to make a policy; established systems of policymaking; steering committees; private sector involvement in policymaking; and administrative working groups including only public sector officers.
Ramirez Varela et al., 2016 [32]	- The PA plan entitled *On the Move, National Strategy for promoting health and wellbeing 2020* is available.
Tammelin et al., 2016 [201] [202]	- The assigned grade for the indicator *Government Strategies, Policies, and Investments* from *RC on PA for Children and Youth* is B.<br>- *New Sport Act* (2015) regulates the aims related to PA at the national level.<br>- The budget for improving sport and PA was 148M Euro in 2015, managed mainly by the Ministry of Education and Culture.
Kahlmeier et al., 2015 [127]	- The following national PA recommendations were identified: *Physical activity recommendations for 7 to 18 years of age* issued in 2008 by the Children and young people's physical activity expert group of the Young Finland Association; and *Early childhood education: physical activity recommendations* issued in 2005 by the Ministry

	of Social Affairs and Health, the Ministry of Education, and Young Finland Association.<br>- Specific recommendations are provided for two age groups: less than 7 y.o. and 7-18 y.o.<br>- The PA recommendations are not fully aligned with the WHO recommendations.<br>- The *Physical activity recommendations for 7 to 18 years of age* include recommendations on muscle-strengthening activities and SB.
Bull et al., 2014 [85] [83] [84]	- The *Resolution concerning the development of health enhancing physical activity and diet* was published by the Government in 2008.<br>- The national PA recommendations for children and youth were published in 2008.<br>- At the governmental level, political commitment to HEPA promotion is excellent.
Christiansen et al., 2014 [44]<br>World Health Organization, 2011 [231]	- Finland has an overall policy related to HEPA and sport promotion: Government resolution on policies promoting sport and physical activity and a its related action plan *Government resolution on policies promoting sport and physical activity*. Both documents were published in 2009 and in this study they were referred to as a single strategy<br>- The strategy referred to the national policy/legislation framework including: the *Public Health Act* (2004); the *Sports Act* (1998); and the *Local Government Act* (1995). It highlighted that everybody should have equal opportunity to pursue way of life that includes sport, and that sport is a vital element of the individual and communal well-being.<br>- The strategy mentioned that local authorities are accountable for the sports service structure.
Liukkonen et al., 2014 [145]<br>Gråstén et al., 2014 [226]	- The assigned grade for the indicator *Government - Strategies, Policies, Investments* from *RC on PA for Children and Youth* is B.<br>- *The Sport Act*, enacted in 1980, regulates PA aims at all levels.<br>- The budget for improving sport and PA was €147M in 2013, managed by the Ministry of Education and Culture, focused mainly on PA promotion in elderly and children.
Kalman & Hamrik, 2013 [128]<br>(in Czech language)	- In a policy document (name not specified) related to PA, a it is mentioned that healthy eating and the promotion of PA are aims implemented in many other documents and within a number of sectors. Ministries responsible for the sectors such as health, education, agriculture, economy, industry, transport, and communication should jointly collaborate on the promotion of PA, healthy nutrition, and general well-being.
Rütten et al., 2013 [185]	- The following public policies related to LTPA were identified: *Sport development Plan 2001-2010*; *Government resolution on policies to develop HEPA* (2002); *Sport Facility Management Direction 2011*; *Promoting pedestrian and bicycle traffic in Finland* (JALOIN, 2001-2004); *National Sport Facility Plan* (1979); and *Sports Act* (1980).<br>- Supportive environments for LTPA (indoor/outdoor sport facilities, infrastructure for LTPA, and urban/green space for LTPA) are broadly available.
Ceccarelli et al., 2011 [90]	- The identified goals in policies that deal with nutrition, PA, and obesity are general and not quantifiable and measurable.<br>- A document (details not specified) provided a thorough analysis of PA and eating habits of the population.<br>- *Health, food and physical activity: Nordic Plan of Action on better health and quality of life through diet and physical activity* was issued by the Nordic Council of Ministers in 2006. The document established a number of common goals and the policy agenda for all Nordic countries, although individual countries have adopted, "partly different sets of actions within the designated areas of common priority".
Brown et al., 2011 [43]	- The national policy document *Government resolution: On development of guidelines for health-enhancing physical activity and nutrition* was published by the Ministry of Social Affairs and Health in 2008.
World Health Organization, 2010 [12]	- The following national policy documents related to PA were identified: *Government resolution on development guidelines for health- enhancing physical activity and nutrition* (2008); *Physical activity and well-being in Finland in the 2010s* (2008); *Nordic Plan of Action on better health and quality of life through diet and physical activity* (2006); and *Promoting pedestrian and bicycle traffic in Finland, the JALOIN programme 2001–2004* (2005).<br>- Since 2008, there has been a coordinating mechanism in the area of PA promotion. The leading institution has been the Council of Physical Activity Promotion. The participating stakeholders are government departments on sport, education and research, health, and transport.

Daugbjerg et al., 2009 [11]	- The policy document *Government Resolution on policies to develop health-enhancing physical activity* (2002) does not contain quantifiable PA goals, budget for policy implementation, and a requirement/intention for evaluation. The time frame is set from 2003 onwards.
World Health Organization, 2007 [227]	- The *Government Resolution on policies to develop health-enhancing physical activity in Finland* mentioned the establishment of HEPA Committee that was set up in 2002. It is linked with the Ministry of Social Affairs and Health and includes representatives from interest groups and other relevant ministries.<br>- In 2001, the Ministry of Transport and Communication started promoting bicycle and pedestrian traffic through the *Jaloin programme*.
Cavill et al, 2006 [89]	- In 1990, National Sports Committee proposed a sports policy for 1990s . One of the main target areas was PA for fitness, recreation, and health among adults.<br>- The programme *Fit for Life* started in 1995 and was active for the two following five-year periods. It is a major HEPA promotion programme that was based on the pilot programme *Finland on the Move*.
Bull et al., 2004 [26]<br>Schöppe et al., 2004 [187]<br>Bull et al. in Bull et al., 2004 [86]	- *Sports Act* (1980, Ministry of Education), was the first relevant policy document that emphasised fitness, *sports for all*, and health and directed state subsidies to local authorities for the construction of sports areas. Its later version, published in 1999 highlighted the value of health benefits achieved by PA promotion, promotion of tolerance, equality and sustainable development.
Vuori et al., 2004 [10]	- In Finland, for more than 150 years health has been one of the major social values of sport<br>- Since 1980, the health sector has held a strong position in relation to PA. The *Report of the Ministry to the parliament on health policy* (1985) and *Health for all by the Year* 2000 (1986) emphasised the importance of HEPA in daily environments. Therefore, key recommendations were to increase availability of cycling and walking paths and recreational areas.<br>- *The Action Plan for Promoting Finnish Heart Health* (1997), published by the Ministry of Social Affairs and Health, emphasised PA as one of its key areas. It was a result of an extensive collaboration where public health institutions and NGOs showed interest in HEPA promotion.
Vuori et al., 1998 [211]	- Two national programmes, *Finland on the Move* and *Fit for Life*, have been successful in creating many new local PA promotion initiatives as well as in increasing PA levels of the population. Both programmes are based on scientific evidence.<br>- *Fit for Life* programme focuses mainly on the target group of 40 to 60 years old adults.
Van Mechelen in Simonopoulos (ed.), 1997 [210]	- There is a national policy for the promotion of PA or physical fitness.<br>- One of the nationwide initiatives aims to increase the proportion of the active population of adults 40 to 60 years old, by 10%<br>- Nationwide governmental action *Finland on the Move* aimed to organise local sport centres for PA promotion for the entire population.
France	
Prévot-Ledrich et al., 2016 [172] (in French language)	- There is no integrated national policy for HEPA promotion that would include all relevant components.<br>- The current French public policies do not explicitly provide the necessary conditions for active lifestyles.<br>- Multiple strategies have been used for PA promotion but they do not cover all sectors.<br>- The *Health at Work Plan* does not include any specific HEPA measure.
Ramirez Varela et al., 2016 [32]	- PA is mentioned in the document entitled *Programme National Nutrition Sante 2011 - 2015.*
Vallgårda, 2015 [209]	- In 2010, *French Obesity Plan 2010-2013* was published by the Ministry of Social Affairs and Health. It stresses out that an increase in PA can be achieved by integrating goals from this plan into the urban policy.
Kahlmeier et al., 2015 [127]	- The national PA recommendations were published in the document entitled the *Physical activity and health: scientific arguments, practical approaches,* issued in 2001.<br>- Specific recommendations are provided for children/young people, adults, and older adults.<br>- The PA recommendations are not fully aligned with the WHO recommendations.<br>- The document includes recommendations on muscle-strengthening activities for

	adults.<br>- The document does not include recommendations on SB.
Rütten et al., 2013 [185]	- The following public policies related to LTPA were identified: the *National plan on prevention through physical activity and sport* (2008) and the *Second National Plan Health-Environment 2009-2012,* and *National Nutritional Health Programme 2011-2015.*<br>- "Sport for All" development, especially for those from deprived areas and for young women, is supported by the National Centre for the Development of Sport.<br>- Supportive environments for LTPA (indoor/outdoor sport facilities, infrastructure for LTPA, and urban/green space usable for LTPA) are broadly available.
Ceccarelli et al., 2011 [90]	- The following quantifiable target related to PA is stated in the analysed policy document (details not specified): 25% of the population should accumulate at least 30 minutes of PA per day, five days a week.
World Health Organization, 2010 [12]	- The following national policy documents related to PA were identified: *Nutrition and physical activity in schools* (2009); *Charter to promote healthy diet and physical activity in television programmes* (2009); *Second national action plan environment and health 2009– 2013* (2009); *National plan on disease prevention through physical activity and sport* (2008); *Second national programme on nutrition and health 2006–2010* (2006); N*ational Plan on Healthy Ageing 2007–2009* (2005); *Sedentariness and physical inactivity* (2003); and *National programme for nutrition and health 2001–2005* (2001)<br>- Since 2001, there has been a coordinating mechanism in the area of PA promotion, with the leading institution being the Ministry of Health and Sports. The participating stakeholders are: government departments on food, health, agriculture, trade and economy, education and research, finance, sport, consumer affairs, and social welfare; communities; academia; civil society; the private sector; and NGOs.
Bréchat et al., 2009 [82]<br>(in French language)	- 14 national programmes based on the rationale that PA and sport are important factors for public health were drafted and implemented between 2001 and 2006.<br>- The "success conditions" of each national action were identified and they were classified according to their potential efficacy for increasing PA. The three actions with the best scores were: national plan *Ageing well*; *The inter-regional programme for consultation on seniors' physical aptitude (Pi-CAPs)*; and the *Programme for the promotion of health through sport and PA and prevention of doping behaviours* (PN-APSD).
World Health Organization, 2007 [227]	- *La santé vient en bougeant* (2004) are complementary guidelines on PA tailored to specific target groups.<br>- *J'aime manger, j'aime bouger* (2004) are separate guidelines addressing both PA and nutrition, and they target adolescents only.
Van Mechelen in Simonopoulos (ed.), 1997 [210]	- There are no national policies for the promotion of PA or physical fitness. France has some nationwide programmes for the promotion of physically active lifestyles, and they have a narrow scope.
French Polynesia	
Ramirez Varela et al., 2016 [32]	- NCD plan includes PA (details are not specified).
Georgia	
Ramirez Varela et al., 2016 [32]	- PA is mentioned in the document entitled *Decree of Government # 2 of II January 2017 regarding National strategy for NCD Prevention and Control plan for years 2017-2020.*
Kahlmeier et al., 2015 [127]	- National PA recommendations have not yet been developed.
Lachat et al., 2013 [140]	- In 2006, the Ministry of Labour, Health and Social Affairs—Public Health Department issued *Food security, healthy eating & physical activity national policy*.<br>- National policy includes the following targets and actions for PA promotion: ensure that the nationwide promotion of social, health, and economic PA benefits is evidence-based; enable public use of the facilities for school sport; design environment suitable for PA; develop policy and legislation for PA promotion at the local level; health sector should take a key role in the decision making process by creating networks with stakeholders and relevant sectors; allocate a part of sports funds to PA promotion; and policy related to urban planning should include secure cycling and walking routes, a plan for stadia, and a plan for recreational facilities.<br>- The policy included detailed actions and specified implementation plan for the

	stakeholders.
Branca et al., 2007 [80]	- A policy document entitled *Food security, healthy eating and physical activity. National Policy, Food and Nutrition Action Plan for Georgia 2006–2010* was published in 2006 by the Ministry of Labour, Health and Social Affairs.
World Health Organization, 2007 [227]	- The action plan related to healthy eating, PA and for security (2006) aims to: promote PA; create monitoring systems for nutrition, PA, and food security; and cooperate with international organisations regarding healthy eating, PA, and food safety.
Germany	
Ramirez Varela et al., 2016 [32]	- The PA plan entitled *INFORM - Germany's national initiative to promote healthy diets and physical activity* is available.
Kahlmeier et al., 2015 [127]	- National PA recommendations have not yet been developed.
Vallgårda, 2015 [209]	- *IN FORM - German National Initiative to Promote Healthy Diets and Physical Activity. The National Action Plan for the prevention of Poor Dietary Habits, Lack of Physical Activity and Related Diseases* was issued in 2008 by the Ministry of Food, Agriculture, and Consumer protection and the Ministry of Health. It aims to provide special support and attention to families with a migrant background and socially disadvantaged families.<br>- The plan aims to govern, but at the same time it allows people to decide for themselves.
Rütten et al., 2013 [185]	- The following public policies related to LTPA were identified: *National Action Plan on Nutrition and Physical Activity* (2008) and *National Cycling Strategy 2002-2012.*<br>- Federal government assigns a stronger role for PA and sport promotion to the states (*Bundesländer*) and the local administration.<br>- Supportive environments for LTPA (indoor/outdoor sport facilities, infrastructure for LTPA, and urban/green space for LTPA) are broadly available.
World Health Organization, 2011 [231]	- The document *German national initiative to promote healthy diets and physical activity* combines PA and diet and sport is addressed as a subtopic.
World Health Organization, 2010 [229]	- The *IN FORM* initiative has a goal to promote PA and healthy diet. It refers to the action plan on the lack of PA, prevention of poor dietary habits, overweight, and related chronic diseases. The action plan ensured networking and collaboration between the health-care system, policy-makers, businesses, civil society, actors in the social field, and the media.
World Health Organization, 2010 [230]	- The Federal Government outlined children's health promotion as a priority issue. The Ministry of Health issued a strategy entitled *Strategie der Bundesregierung zur Förderung der Kindergesundheit* in 2008. The strategy mentions initiatives and activities related to children's PA, mental health, injury prevention, environmental health, and well-being. A special focus is on children with a migrant background and/or socially disadvantaged children.<br>- In 2004, the Government established the platform *Nutrition and physical activity* to promote healthy nutrition and PA and encourage people to fight against obesity.
World Health Organization, 2010 [12]	- The following national policy documents related to PA were identified: *IN FORM – Germany's initiative for a healthy nutrition and more physical activity* (2008); *National Strategy for the Promotion of Child Health* (2008); *National action plan for a child-friendly Germany 2005–2010* (2005); and *National Cycling Plan 2002–2012 "Ride your bike!"* (2002).<br>- Since 2007, there has been a coordinating mechanism in the area of PA promotion. The leading institutions have been the Federal Ministry of Health and the Federal Ministry of Food, Agriculture, and Consumer Protection as leading institutions. The participating stakeholders are: government departments on agriculture, labour, transport, culture, social welfare, education and research, health, consumer affairs, sport, food, and urban planning; communities; academia; civil society; the private sector; and NGOs.
Daugbjerg et al., 2009 [11]	- The policy document *National Cycling Plan 2002-2012 "Ride your bike!"* (2002) does not contain quantifiable PA goals and a budget for policy implementation, but it contains a requirement/intention for evaluation.
Bergsgard et al., 2007 [76]	- "Sport for All", mass sport, was well established by the late 1970s but it is mainly the responsibility of local/state (*Bundesländer*) level governments and not national, that is federal level government. At the national level, the key initiatives are undertaken by the national sports federations and not by the Government.

	- The “national federal ministries” provide funding to the *German Sport Confederation* to deliver programmes related, for example, to women’s sports, family sports, sports for elderly, and the integration of minority groups in sports.
World Health Organization, 2007 [227]	- The German Platform for Diet and Physical Activity, was established in 2004 and is a good example of integrating and mobilising stakeholders from different groups such as government representatives, scientific organisation, trade unions, sports unions, parents’ organisations, food industry, health insurance associations, and food producers.<br>- The Federal Ministry of Transport, Building, and Housing issued the *National cycling plan 2002–2012 – ride your bike*! to promote cycling in Germany.
Van Mechelen in Simonopoulos (ed.), 1997 [210]	- There are no national policies for the promotion of PA or physical fitness. There is no clear picture regarding nationwide activities for the promotion of physically active lifestyle.
Ghana	
Ocansey et al., 2016 [164]	- The assigned grade for the indicator *Government – Strategies, Policies, Investments* from *RC on PA for Children and Youth* is D.<br>- There is no official policy by the Ministry of Education on school sport and PE. There is only syllabi and requirements for the participation in PE.<br>- No national recommendations/guidelines for built environment to promote PA in youth and children are available.
Ramirez Varela et al., 2016 [32]	- A national or subnational PA plan is available (details are not specified).
Ocansey et al., 2014 [163]	- The assigned grade for the indicator *Government – Strategies, Policies, Investments* from *RC on PA for Children and Youth* is D.<br>- There is no policy on after-school sport, although national school sport events and festivals are organised biannually.<br>- Policies related to PA should be developed.
Lachat et al., 2013 [140]	- In 2007, The Ministry of Health issued *National health policy: creating wealth through health*.<br>- National policy includes the following targets and actions for PA promotion: let PA education become obligatory in schools; and encourage regular exercise.
Greece	
Ramirez Varela et al., 2016 [32]	- A national or subnational PA plan is available (details are not specified).
World Health Organization, 2010 [229]	- Policy documents in the PA area are not yet available.
World Health Organization, 2010 [12]	- National policy has not yet been developed.<br>- There is no coordinating mechanism in the area of PA promotion.
Matalas in Simonopoulos (ed.), 1997 [152]	- The Government introduced PE as an obligatory subject in schools in 1899 (*Themidos Code*)<br>- The Office for the Development of Sports, Ministry of Culture, founded in 1985, had a key mission to promote *Sports for All*. Some of the sports for all programmes implemented between 1991 and 1993 include programmes for children, young people, women, men in the military service, people with special needs, addicts, and prisoners.
Greenland	
Ramirez Varela et al., 2016 [32]	- PA is mentioned in the document entitled *Inuuneritta 2013-2019.*
Ceccarelli et al., 2011 [90]	- *Health, food and physical activity: Nordic Plan of Action on better health and quality of life through diet and physical activity* was issued by the Nordic Council of Ministers in 2006. The document established a number of common goals and the policy agenda for all Nordic countries, although individual countries have adopted “partly different sets of actions within the designated areas of common priority”.
Grenada	
Ramirez Varela et al., 2016 [32]	- PA is mentioned in the document entitled *Strategic Plan of Action for the Prevention and Control of Non-communicable diseases for countries of the Caribbean Community 2011-2015.*
Guam	
Ramirez Varela et al., 2016 [32]	- PA is mentioned in the document entitled *Guam NCD Strategic Plan 2014 – 2018.*

Guatemala	
Ramirez Varela et al., 2016 [32]	- PA is mentioned in the document entitled *2008 - 2012 Action Plan for the Prevention and Integral Control of Chronic Diseases and Risk Factors.*
Lachat et al., 2013 [140]	- National policy includes the (details not specified) following targets and actions for PA promotion: apply measures and strategies that support good health and include PA, particularly in schools and at worksites; and organise multisectoral workshops for the development of national and local PA networks.
Guyana	
Ramirez Varela et al., 2016 [32]	- PA is mentioned in the document entitled *Strategic plan for the integrated prevention and control of chronic non-communicable diseases and their risk factors 2013-2020.*
Lachat et al., 2013 [140]	- In 2008, the Ministry of Health issued the *National health sector strategy*.<br>- National policy includes the following targets and actions for PA promotion: promote PA in schools and communities; by 2010 make PA an examinable subject in all schools.<br>- The policy includes strategies for PA promotion focused on workplaces.
Haiti	
Ramirez Varela et al., 2016 [32]	- PA is mentioned in the document entitled *Strategic Plan of Action for the Prevention and Control of Non-communicable diseases for countries of the Caribbean Community 2011-2015.*
Hong Kong SAR, China	
Huang et al., 2016 [124] [125]	- The indicator *Government - Strategies, Policies, Investments* from *RC on PA for Children and Youth* did not receive a grade. It was marked as incomplete.<br>- The Leisure and Cultural Services Department is responsible for sport and recreation. In the local community it has promoted “Sport for All” and organised School Sports Programmes for students to engage in sport activities during leisure time.<br>- Department of Health issued the *Physical Activity Guide for Children Aged 2 to 6* (2011) and the *Report of Advisory Group on Health Effects of Use of Internet and Electronic Screen Products* (2014). The report stated that children (2-6 y.o.) should limit screen time to maximum two hours per day and avoid sitting for more than one hour. Six to twelve years old children should limit their recreational screen time to maximum two hours per day. For youth from 12 to 18 years old, it is recommended to avoid prolonged screen time. There is limited awareness on guidelines related to SB among the relevant stakeholders.
Ramirez Varela et al., 2016 [32]	- The PA plan entitled *Action Plan to Promote Healthy Diet and Physical Activity Participation in Hong Kong* is available.
Hungary	
Ramirez Varela et al., 2016 [32]	- PA is mentioned in the documents entitled *National Sport Strategy 2007 -2020. National Old-age Policy (Strategy) (2009), Semmelweis Plan for the Rescue of Health Care (2011).*
Kahlmeier et al., 2015 [127]	- It is reported that national PA recommendations exist, but no other details are available.
Christiansen et al., 2014 [44] World Health Organization, 2011 [231]	- The document *Sport XXI. National sports strategy 2007–2020 [Sport XXI. Nemzeti sportstrategia 2007–2020],* issued in 2007, included people with disabilities as a target group. It mentions the emphasis needs to be put on leisure sport for people with disabilities.
Ceccarelli et al., 2011 [90]	- The analysed policy document (details not specified) explicitly refers to the *WHO Global Strategy on Diet, Physical activity and Health* or to some other document assessing the problem of obesity.<br>- The quantifiable targets reported in the analysed policy document (details not specified) are: increase the number of people by 15% who participate in sport as intensively and as long as it is deemed necessary from the physiological perspective, and increase at least by 25% the level of exercise of those people who are not sufficiently active.
World Health Organization, 2010 [12]	- The following national policy documents related to PA were identified: *National Youth Strategy 2009-2024* (2009); *National Sport Strategy, Parliament Resolution* (2007); *Parliamentary Resolution 47/2007 (V31) OGY on the National Strategy 2003 – 2032 to improve the situation of children* (2007); *Cycling Hungary Programme 2007 – 2013* (2007); *“Johan Bela” National Programme for the Decade of Health* (2003); and *Act on Public Education* (1993).

	- There has been a coordinating mechanism in the area of PA promotion, with the Ministry of Local Government as the leading institution. The participating stakeholders are government departments on sport, health, and transport; civil society; and NGOs.
Daugbjerg et al., 2009 [11]	- The policy document *Johan Bela'—National Programme for the Decade of Health*, issued in 2003, contains quantifiable PA goals, but it does not contain a budget for policy implementation and a requirement/intention for evaluation.<br>- The document *National Public Health Programme—Action Plan*, issued in 2004, does not contain quantifiable goals, budget, and an evaluation requirement/intention.
Branca et al., 2007 [80]	- The Ministry of Health, Social and Family Affairs issued the *"Johan Bela" National Programme for the Decade of Health* in 2003 and the *National Public Health Programme – Summary* in 2004.
Iceland	
Ramirez Varela et al., 2016 [32]	- A national or subnational PA plan is available (details are not specified).
Kahlmeier et al., 2015 [127]	- The national PA recommendations were published in the document entitled the *Recommendations for physical activity,* issued in 2008.<br>- Specific recommendations are provided for children/young people, adults, and older adults.<br>- The PA recommendations are not fully aligned with the WHO recommendations.<br>- The document includes recommendations on muscle-strengthening activities for children/young people, adults, and older adults.<br>- The document includes recommendations on inactivity for children/young people, adults, and older adults and on screen-time for children/young people.
Ceccarelli et al., 2011 [90]	- The analysed policy document (details not specified) explicitly refers to the WHO Global Strategy on Diet, Physical activity and Health or to some other document assessing the problem of obesity.<br>- The document includes a thorough analysis of PA and eating habits of the population. The time frame for achieving goals is ten years.<br>- The quantifiable goals mentioned in the policy document are the following: achieve at least 30 minutes of MVPA every day for adults; and achieve at least 60 minutes of MVPA daily for children and adolescents.<br>- *Health, food and physical activity: Nordic Plan of Action on better health and quality of life through diet and physical activity* was issued by the Nordic Council of Ministers in 2006. The document established a number of common goals and the policy agenda for all Nordic countries, although individual countries have adopted, "partly different sets of actions within the designated areas of common priority".
Daugbjerg et al., 2009 [11]	- Policy documents *The Icelandic National Health Plan to the year 2010—abridged version* (2001) and *The National Health Promotion Strategy 2000-2005* do not contain quantifiable PA goals and budget for policy implementation but contain a requirement/intention for evaluation.
World Health Organization, 2007 [227]	- *The National health plan to the year 2010*, issued in 2001, emphasised the importance of a healthy lifestyle, especially addressing PA and exercise for people of all age groups.<br>- In 2003, the Public Health Institute of Iceland was established and one of its key focus areas is health promotion through proper nutrition and PA.<br>- In 2005, the Parliament passed a resolution that calls for action to improve population's health through increased PA and healthier diet.<br>- A working group was established by the Minister of Education, Science, and Culture in 2005, with a goal to create a sports policy. They issued *The progress report, Sporty Iceland* in 2006. A formal sport policy document is under development. Its key emphasis is put on the significance of daily exercise and how society can develop a forum for a healthy lifestyle.
India	
Katapally et al., [130] [131]	- The assigned grade for the indicator *Government – Strategies, Policies, and Investments* from *RC on PA for Children and Youth* is D.<br>- Several policy documents on PA promotion including PA guidelines have been issued by the Government, but there is no clear evidence of their implementation.<br>- There is a lack of policies related to active living urban planning for enhancement of walkability.<br>- Most government's strategies are focused on competitive/elite sport.

Ramirez Varela et al., 2016 [32]	- A national or subnational PA plan is available (details are not specified).
Lachat et al., 2013 [140]	- In 2008, India Planning Commission Government issued *Eleventh five-year plan: 2007–12*.<br>- National policy includes the following targets and actions for PA promotion: build sport infrastructure at grassroots level in urban and rural parts; incorporate PE into the school system; modify sports policy, services, and action plan; and involve the corporate sector in the establishment of the sport culture.<br>- The policy included concrete actions for the involvement of the private sector in PA promotion.<br>- The policy targeted community at large.
Indonesia	
Ramirez Varela et al., 2016 [32]	- A national or subnational PA plan is available (details are not specified).
Lachat et al., 2013 [140]	- In 2005, Indonesia National Development Planning Board, issued the *National action plan for food and nutrition 2006–2010*.<br>- National policy includes the following targets and actions for PA promotion: increase PA at the population level by increasing promotion efforts; in the frame of building public awareness at all levels of society, enhance understanding of PA benefits; increase funding and the number of open space and sport facilities.<br>- The policy targeted community at large.
Iran, Islamic Rep.	
Lachat et al., 2013 [140]	- In 2010, the Iranian Ministry of Health and Medical Education—Nutrition Department issued the *Operational plan to improve community nutrition*.<br>- The policy stated that students' obesity and overweight should be controlled and prevented by increasing PA.
World Health Organization, 2014 [232]	- There is a national coordinating committee for population PA.<br>- National action plan and policy related to PA are under development (details not specified).<br>- Besides the general population, the population groups that are covered in national policy documents (under development) are: children and young people; older adults; workforce/employees; women; clinical population with chronic disease; low socioeconomic groups; and families.<br>- The settings covered by national policy documents (under development) are: primary schools; high schools; primary health care; clinical health care; workplaces; senior/older adult services; sport and leisure; transport; environment; and urban design/planning.
Iraq	
Ramirez Varela et al., 2016 [32]	- PA is mentioned in the document entitled *Non-Communicable Diseases and Mental Health Control and Prevention Programme.*
World Health Organization, 2014 [232]	- There are national NCD strategic plans or policies that include goals focused on PA (details not specified).<br>- No specific target groups are mentioned, only general population is covered in national policy documents.<br>- The settings covered by national policy documents are: primary schools; primary health care; sport and leisure; and transport.
Ireland	
Harrington et al., 2016 [120] [121]	- The indicator *Government* from *RC on PA for Children and Youth* did not receive a grade. It was marked as incomplete.<br>- Multisectoral promotion of PA in children through the education, health, sport, and transport sectors is highlighted in strategy and policy documents<br>- *Get Ireland active! National Physical Activity Plan for Ireland*, issued in 2016 by the Department of Health and the Department of Transport, is innovative, because it sets out 60 actions and identifies lead agencies and responsible partners and the timeline for its delivery.<br>- The reduction of SB and its replacement with PA is recommended in *Get Ireland Active! The National Guidelines on Physical Activity for Ireland* issued in 2009 by the Department of Health and Children.
Ramirez Varela et al., 2016 [32]	- No national/subnational PA plan.

Kahlmeier et al., 2015 [127]	- The national PA recommendations were published in the document entitled *The National Guidelines on Physical Activity for Ireland,* issued in 2009.<br>- Specific recommendations are provided for children/young people, adults, and older adults.<br>- The PA recommendations are not fully aligned with the WHO recommendations.<br>- The document includes recommendations on muscle-strengthening activities for children/young people and older adults.<br>- The document does not include recommendations on SB.
Christiansen et al., 2014 [44]<br>World Health Organization, 2011 [231]	- Two documents were published in 2008: *Building sports for life: the next phase – the Irish Sports Council's strategy 2009–2011* (an action plan) and the *Statement of Strategy 2008–2010* (Department of Arts, Sport and Tourism). In this study, the two policy documents were referred to as a single strategy<br>- The strategy mentioned that both external and internal consultations took place involving government agencies, boards, key sectors, and the public.
Harrington et al., 2014 [118] [119]	- The indicator *Government* from *RC on PA for Children and Youth* did not receive a grade. It was marked incomplete.<br>- There are no specific PA targets/goals and no national PA plan.<br>- Policy documents related to active travel are the following*: Ireland's First National Cycle Policy Framework 2009-2020*; and *Smarter Travel – A Sustainable Transport Future A New Transport Policy for Ireland 2009 – 2020.*
Kalman & Hamrik, 2013 [128]<br>(in Czech language)	- In a policy document (details not specified) related to PA it is mentioned as a problem that 42% of people are engaged in some form of PA but the percentage is reducing with increase in age. Almost 1/3 of people older than 55 are not engaged in any weekly PA.
Woods & Mutrie, 2012 [218]	- National PA policy does not exist, that is clear national vision with strategic plan, resources, clear PA goal or evaluation mechanisms.<br>- In 2010, Health Service Executive set up working group to provide draft of the national PA plan.
Brown et al., 2011 [43]	- *The National Guidelines on Physical Activity for Ireland*, published by the Department of Health and Children Health Service Executive in 2009 were not recommended according to the Appraisal of Guidelines for Research and Evaluation quality grading (AGREE tool).
Ceccarelli et al., 2011 [90]	- The analysed policy document (details not specified) explicitly refers to the *WHO Global Strategy on Diet, Physical activity and Health* or to some other document assessing the problem of obesity.<br>- The quantifiable goals in the policy document for the next 15 years and over are the following: increase the number of people who engage in accumulated 30 minutes of light physical exercise throughout most days of the week by 30%; and increased the number of people who engage in moderate physical exercise at least 20 minutes three times a week by 20%.
World Health Organization, 2010 [230]	- In 2000, the Department of Health and Children issued *The national children's strategy: our children – their lives* that, a ten-year policy framework. One of the objectives of the framework is that all children gain access to sport, play, cultural activities, and recreation, to enrich their childhood.<br>- Another important policy documents is *Ready, steady, play! A national play policy*, issued by the National Children's Office in 2004.<br>- *Teenspace: national recreation policy for young people* issued in 2007 by the Office of Minister for Children set several objectives, including giving young people a voice in the implementation, design, and monitoring of recreation facilities and policies.
World Health Organization, 2010 [12]	- The following national documents related to PA were identified: *National Men's Health Policy* (2008) and *The National Health Promotion Strategy 2000–2005* (2000).<br>- Since 2009, there has been a coordinating mechanism in the area of PA promotion, with Department of Health and Children as the leading institution. The participating stakeholders are: government departments for culture, health, transport, trade and economy, agriculture, sport, urban planning, labour, social welfare, food, education, and research; academia; NGOs; and the private sector.
Branca et al., 2007 [80]	- The following national policy documents were identified: *Obesity: the policy challenges*. *The report of the National Taskforce on Obesity* (2005) and *The National Health Promotion Strategy 2000–2005* (2000). Both documents were issued by the Department of Health and Children.

World Health Organization, 2007 [227]	- Issues around nutrition and PA promotion are mentioned in the *National health promotion strategy 2000–2005*.<br>- The Department of Arts, Sports, and Tourism, established in 2002, had the objective to promote sport especially in disadvantaged areas and create sustainable facilities.
Van Mechelen in Simonopoulos (ed.), 1997 [210]	- There are no national policies for the promotion of PA or physical fitness.
Israel	
Ramirez Varela et al., 2016 [32]	- PA is mentioned in the document entitled *Healthy Israeli 2020 Initiative.*
Kranzler et al., 2013 [137]	- Working groups of the *Healthy Israel 2020* initiative related to PA, nutrition, and obesity suggested policy guidelines and goals. That was the first stage in the development of the *National Programme to Promote Active, Healthy Lifestyle* that was launched in 2010. It represents a paradigm shift for the Israel Ministry of Health, become health promotion was previously marginalised when compared to some other areas the Ministry focused on.<br>- The Programme sets the following quantifiable targets: increase the number of those who engage in the recommended amount of PA by 20% among Jewish boys and by 35% among Jewish and Arab girls and Arab boys; decrease the number of children who watch television two or more hours per day by 20%; and increase the number of those who engage in the recommended amount of PA by 30% among Arab women, by 20% among Jewish men; and 25% among Jewish women and Arab men.<br>- Some PA promotion initiatives include: website by the Ministry of Health and Education that contains games and other activities to make children excited about the healthy and active lifestyle; training for nurses on education of parents to encourage children to be more active and eat better; a pilot programme for the promotion of health at workplaces that will be launched in 2013 by the Ministry of Health.<br>- In 2012, the Ministry of Culture and Sport conducted *The First National Survey of Physical*<br>*Activity in Israel.*
World Health Organization, 2007 [227]	- In 2005, the Health Promotion Council, Ministry of Health established the Obesity Task Force and the Physical Activity Task Force.
Italy	
Hämäläinen et al., 2016 [115]	- A national project for the promotion of PA was based on the policy *Gaining Health* issued in 2007 by the Ministry of Health.<br>- HEPA policies cooperation and coordination processes and structures include: government/regional/local committees or working groups with cross-sector representatives; national/regional/local politically elected councils; contacts between public sector officers responsible for HEPA between levels; private sector involvement in policymaking; and public hearings for citizens.<br>- There are no: field visits to make a policy; established systems of policymaking; intersectoral committees or working groups for HEPA; scientific advisory groups/institutes/individuals; steering committees; administrative working groups including only public sector officers; and formal consultations on HEPA policy for stakeholders.<br>- The overall coordination features for HEPA policies are: rather decentralised, strong individuals, network arrangements, and private sector involvement.
Ramirez Varela et al., 2016 [32]	- PA is mentioned in the document entitled *Gaining health.*
Kahlmeier et al., 2015 [127]	- National PA recommendations have not yet been developed.
Bull et al., 2014 [85] [83] [84]	- Within the *Gaining Health Programme*, launched in 2008, a three year (2007-2010) national project on PA promotion was carried out. The project was called *Promoting Physical Activity – Actions for a Healthy Life*<br>- *National Prevention Plan 2010 – 2012* includes the reduction of obesity and the promotion of a healthy style as public health priorities.<br>- Sport development and management are the responsibility of the Italian National Olympic Committee. The Ministry of Health (General Directorate of Prevention, Healthy Lifestyle Unit and Public Health and Innovation Department) is responsible for HEPA promotion.<br>- The key law related to PA and sport in schools is *Decree 18/12/1975*

	- National PA recommendations do not exist.<br>- There are no national goals for PA.
Ceccarelli et al., 2011 [90]	- The identified goals in policies (details not specified) that deal with nutrition, PA, and obesity are general and not quantifiable and measurable.<br>- The analysed policy document (details not specified) explicitly refers to the *WHO Global Strategy on Diet, Physical activity and Health* or to some other document assessing the problem of obesity.
World Health Organization, 2010 [12]	- The following national documents related to PA were identified: *Gaining health* (2007), the *National plan for prevention 2005-2007* (2005) and the *National Health plan 2003-2005* (2003)<br>- There is no coordinating mechanism in the area of PA promotion.
World Health Organization, 2007 [227]	- In 2005, the Ministry of Health issued a *National Plan of Prevention 2005–2007.* Its key priority is to fight against obesity, especially in children. Some of the initiatives include: the promotion of PA in the community and developing urban environments that support PA.
Van Mechelen in Simonopoulos (ed.), 1997 [210]	- There are no national policies for the promotion of PA or physical fitness.
Jamaica	
Lachat et al., 2013 [140]	- In 2004, the Ministry of Health issued the *National policy for the promotion of healthy lifestyles in Jamaica*.<br>- National policy includes the following targets and actions for PA promotion: provide green, secure, and clean open space for everyone to participate in PA; establish healthy communities that are suitable for community members to engage in PA; in four years, increase the number of people having moderate PA levels by 40%; establish partnership with media for PA promotion; create life-skills programme for communities, schools, and worksites that includes PA and all aspects of healthy lifestyle; create guidelines on sports and PE for target groups; improve and evaluate facilities for including PA in health service; involve PA as part of chronic disease management; establish laws, regulations, and policies that encourage PA lifestyle and supportive environment in workplaces, communities, and schools; and provide opportunities for young people and children to engage in supervised afterschool sport activities.<br>- The policy includes concrete actions for involvement of private sector in PA promotion.
Japan	
Ramirez Varela et al., 2016 [32]	- PA is mentioned in the document entitled *Health Japan 2.*
Tanaka et al., 2016 [204] [205]	- The assigned grade for the indicator *Government Strategies and Investments* from *RC on PA for Children and Youth* is B.<br>- There are no national recommendations for SB.<br>- Relevant government documents include: *The School Lunch Programme Act*; *the School Health and Safety Act*; *the Basic Act on Sport*; the *Community Health Act*; *Health Promotion Law*; *the Maternal and Child Health Act*; *the Basic Act on Food Education*; *Healthy Parents and Children 21*; the *Sport Basic Plan*; and *Health Japan 21.*<br>- The current PA guidelines are only available for elderly, adults, primary school children, and preschool children. There are no guidelines for adolescents.<br>- The *Sports Authority*, an external organ of the Ministry of Education, Culture, Sports, Science, and Technology and the Ministry of Health, Labour, and Welfare are responsible for policies related to PA, exercise, and sports.<br>- The National Budget for Sports and the Sports Promotion Lottery are responsible for the investment and implementation of the national policy.
Ceccarelli et al., 2011 [90]	- The identified goals in policies (details not specified) that deal with nutrition, PA, and obesity are general and not quantifiable and measurable.<br>- The analysed policy document (details not specified) does not specify a time frame to reach the targets.
Pate et al., 2011 [37]	- Policy related to PE in schools states that: children should engage in at least two hours of PE a week; PE should be taught by highly qualified and certified teachers; and PE curriculum should be included in the school review process.

	- PA policy within the area of health education states that: cooperation with universities should be established to provide teacher education classes for inclusion of the *PA and Health* topic into the curriculum; training in motivational interviewing techniques related to PA should be provided to healthcare professionals in schools; the promotion of PA for achieving health benefits should be incorporated in health education classes; education should be provided to parents to encourage them to regularly engage in PA and to be positive role models for their children.<br>- The data were extracted from the *Basic Plan for the Promotion of Sports 2001 – 2010* issued by the Ministry of Education, Culture, Sports, Science and Technology.
Koh, 2010 [136]<br>(in Korean language)	- The Ministry of Health, Labour, and Welfare of Japan issued the *Exercise and physical activity guide for health promotion* in 2006. The document provides PA recommendations by stages of behaviour change.<br>- The National Institute of Health and Nutrition issued *Exercise and Physical Activity Reference for Health Promotion* in 2006. The document differentiates between moderate or high-intensity exercise (e.g. jogging, swimming, tennis, brisk walking) and low-intensity exercise (e.g. stretching) and also between moderate or high-intensity non-exercise activity (e.g. cleaning floors, washing car, gardening, walking.) and low-intensity non-exercise activity (e.g. standing, doing laundry cooking, playing piano).
Jordan	
Ramirez Varela et al., 2016 [32]	- PA is mentioned in the document entitled *National Strategy And Plan Of Action Diabetes, Hypertension, Dyslipidaemia And Obesity in Jordan.*
World Health Organization, 2014 [232]	- A national coordinating committee related to PA was established, but it no longer exists.
Lachat et al., 2013 [140]	- In 2006, the Ministry of Health, issued the *Nutrition in Jordan: update and plan of action*.<br>- National policy includes the following targets and actions for PA promotion: establish multisectoral committee for PA and develop national strategy for the promotion of PA and exercise.
Kazakhstan	
Ramirez Varela et al., 2016 [32]	- A national or subnational PA plan is available (details are not specified).
World Health Organization, 2010 [229]	- Policy documents in the PA area are not yet available.
World Health Organization, 2007 [227]	- *2010 Health Promotion* is a strategic document that has eight priority areas. One of them is focused on PA and nutrition. The strategy states that the responsibility for action should be put on individuals, government, and employers.<br>- An interdepartmental document related to healthy lifestyles was developed in 1999. Implementation should take place in three phases, from which the first two are committed to setting up a network for NCD prevention at the local, regional, and national level, focusing on PA and nutrition through special training programmes.
Kenya	
Ramirez Varela et al., 2016 [32]	- PA is mentioned in the document entitled *Kenya Health Policy 2014-2030.*
Lachat et al., 2013 [140]	- In 2013, the Ministry of Public Health and Sanitation issued the *National nutrition action plan 2012–2017*.<br>- National policy (details not specified) included the following targets and actions for PA promotion: by 2016/17 the number of people that have a healthy diet and engage in PA should be 15%; educate health workers on PA; and in all counties arrange "sensitisation" meetings on PA.
Onywera et al. 2016 [165] [166]	- The assigned grade for the indicator *Government and Nongovernment (Strategies, Policies, Investments)* from *RC on PA for Children and Youth* is D.<br>- *National Plan of Action for Children*, published by the National Council for Children's Services in 2015,recognises the right of all children to plan, leisure and recreation.<br>- There are no known governmental tactics to tackle built environment and its impact on PA in children.
Wachira et al., 2014 [212] [213]	- The assigned grade for the indicator *Government and Nongovernment (Strategies, Policies, Investments)* from *RC on PA for Children and Youth* is C.<br>- There are several policies that target some of the core indicators related to PA, but

	there is a lack of their implementation.<br>- There are no PA or SB guidelines, national surveillance plan, and PA or active healthy living strategy.<br>- The Ministry of Education mentions the amount of time per week that youth and children are required to participate in PE.
Kiribati	
Ramirez Varela et al., 2016 [32]	- NCD plan includes PA (details are not specified).
Korea, Rep.	
Ramirez Varela et al., 2016 [32]	- The PA plan entitled *The physical activity Guide for Koreans* is available.
Yoonkyung et al., 2016 [221] [222]	- The assigned grade for the indicator *Government Strategies and Investment* from *RC on PA for Children and Youth* is C.<br>- Evidence-based PA and SB guidelines should be developed.<br>- The Ministry of Education promotes PA through *School Sports Activation Project* (elementary school: at least three PE classes/week, middle school: at least 4 PE classes/week, high school: ten PE classes during six semesters). It has also expanded the *School Sport Club programme*, initially launched in 2007. The goals are: to increase participation of students in school sports clubs to 17 or more hours per year; to improve facilities (particularly for girls and younger children); and to increase the proportion of qualified instructors.<br>- The *Integrated Sports Council* was founded by the Ministry of Culture, Sport, and Tourism, the Korea Council of Sports for All, the Ministry of Education, the School Union, the Korea Institute of Sports Science, and the Korean Olympic Committee. They all jointly work on PA promotion.
Ceccarelli et al., 2011 [90]	- The quantifiable targets mentioned in the analysed policy document (details not specified) are: in 2020 increase by 30% the rate of participation in MVPA for 30 minutes a day, five days a week.
Koh, 2010 [136]<br>(in Korean language)	- *Medical Law of Republic of Korea. No. 9386. Article 2* states that physicians have a responsibility to promote national health and contribute to securing people's healthy lives. They have the duty of "health guidance". Therefore, the authors suggest the development of "official" PA guidelines at the national level, with a high level of inclusion of the medical service industry in the process. The guidelines should be developed according to the Transtheoretical model of behaviour change.<br>- Article 2 of the *National Sports Promotion Act* in Korea defined sport as "activities to cultivate a healthy mind and body through PA to make good use of leisure time". "Sport for All" was defined as "sport activities conducted voluntarily in daily life for the promotion of health and fitness".
Kuwait	
World Health Organization, 2014 [232]	- There is a national coordinating committee for PA.<br>- International recommendations and guidelines on PA are used instead of national ones.<br>- The adoption of the global recommendations has been recommended in the draft of the national policy (details not specified). The adoption by the Government is pending.<br>- National policy documents cover: the general population; children and young people; older adults; workforce/employees; women; the clinical population with chronic diseases; sedentary/the most inactive; low socioeconomic groups; and families.<br>- Settings covered by national policy documents that have not yet been adopted: kindergarten; primary schools; high schools; colleges/universities; primary health care; clinical health care; workplace; senior/older adult services; sport and leisure; transport; environment; urban design/planning; desert; cooperative societies; and *dewaniya*.

Ramadan et al., 2010 [175]	- In 2007, the National Physical Activity Committee was established. It included: representatives from the Ministry of Health, Governorates, and NGOs; PA specialists; cardiologists and other physicians; Parliament members; prominent media figures; and university presidents and deans.<br>- The *National Physical Activity Plan* is in the process of development and its key objectives are to: increase the awareness of the importance of PA for improving quality of life and health; contribute to follow-up, prediction, and monitoring of health conditions related to physical inactivity; encourage and inspire community members to regularly participate in PA and use opportunities available for PA participation; build a capacity for PA promotion by conducting workshops and hosting scientific meetings; develop worksite and school policies that will result in increased PA; contribute to the reduction of behavioural and social problems (e.g. alcohol and drug use) by encouraging LTPA; develop built environment policies that will enable people to be more active; decrease the physical inactivity prevalence each year; design and disseminate an example for other countries in the Region.
Kyrgyz Republic	
World Health Organization, 2010 [229]	- Policy documents in the PA area not yet available.
Latvia	
Kahlmeier et al., 2015 [127]	- National PA recommendations have not yet been developed.
Christiansen et al., 2014 [44] World Health Organization, 2011 [231]	- The document related to sport promotion entitled *National sports development programme 2006–2012* [Nacionala sporta attistibas programma 2006–2012] was issued in 2006. It mentioned short-term, medium-term, and long-term time frames. Within the time frame of six years, four main targets were determined for each year of the programme. The key result of the programme implementation should be: an increased number of people engaged in PA; a decline in physical inactivity indicators; and an improvement in the society's health in general.
World Health Organization, 2010 [12]	- The following national documents related to PA were identified: *National sports development programme* 2006–2012 (2006); *Sport policy guidelines 2004–2009* (2004); and *Public Health Strategy* (2001).<br>- Since 2003, there has been a coordinating mechanism in the area of PA promotion, and the leading institution has been the Ministry of Education and Science. The participating stakeholders are government departments on finance, health, welfare and education; academia; and NGOs.
World Health Organization, 2007 [227]	- The documents *Public health strategy* (approved in 2001) and *the Healthy nutrition 2003–2013 – concept of the Cabinet of Ministers* mentioned the need to keep the public informed on healthy lifestyles, nutrition, PA, and food hygiene.
Lao PDR	
Ramirez Varela et al., 2016 [32]	- No national/subnational PA plan.
Lebanon	
Ramirez Varela et al., 2016 [32]	- No national/subnational PA plan.
Lithuania	
Ramirez Varela et al., 2016 [32]	- The PA plan entitled *2011 – 2020 State Sport Development Strategy* is available.
Kahlmeier et al., 2015 [127]	- National PA recommendations were reported to exist, but no other details are available.
Christiansen et al., 2014 [44] World Health Organization, 2011 [231]	- The document related to sport promotion entitled *The physical education and sports strategy 2005–2015* [Lietuvos Respublikos kuno kulturos ir sporto strategija 2005–2015 metams] was issued in 2005. It addressed sport and PE in preschool, primary, and secondary school children, and in university students. For all of the groups, specific sets of objectives were mentioned.
Rütten et al., 2013 [185]	- Public policy related to LTPA *National Sport Development Strategy, 2011-2020* was issued in 2011. This policy is criticised for lacking the principles established in the WHO recommendations and EU PA Guidelines.<br>- Supportive environments for LTPA (indoor/outdoor sport facilities and urban/green space usable for LTPA) are partially available.

World Health Organization, 2010 [12]	- The following national documents related to PA were identified: *State Physical Education and Sport Strategy* (draft) (2010) and *Law on Physical Education and Sports* (2008).<br>- Since 2002, there has been a coordinating mechanism in the area of PA promotion. The leading institutions have been the Ministry of Health, the Ministry of Interior, and the Department on Physical Education and Sports. The participating stakeholders are government departments on urban planning, food, health, sport, social welfare, and education and research; "Sport for All" Association; NGOs; academia; media; communities; and civil society.
World Health Organization, 2007 [227]	- The *State food and nutrition strategy and action plan 2003–2010* was issued in 2003. Some of the measures to reach the targets of the action plan are: the implementation of law on sports and PA; and an obesity control programme at the national level.<br>- National legislation specifies PE in school must be held at least three hours per week.
Luxemburg	
Ramirez Varela et al., 2016 [32]	- The PA plan entitled *Action plan for the promotion of healthy nutrition and physical activity* is available.
Kahlmeier et al., 2015 [127]	- The national PA recommendations were published in the document entitled *Towards a national plan for healthy nutrition and physical activity,* issued in 2007.<br>- Specific recommendations are provided for children/young people and adults.<br>- The PA recommendations are not fully aligned with the WHO recommendations.<br>- The document includes recommendations on muscle-strengthening activities for children/young people and adults.<br>- The document does not include recommendations on SB.
Ceccarelli et al., 2011 [90]	- The identified goals in policies that deal with nutrition, PA, and obesity (details not specified) are general and not quantifiable and measurable.<br>- The analysed policy document (details not specified) explicitly refers to the *WHO Global Strategy on Diet, Physical activity and Health* or to some other document assessing the problem of obesity.
World Health Organization, 2010 [12]	- The *Action plan for human-powered mobility* was issued in 2008 by the Ministry of Transport, the Ministry of Public Works, and the Ministry of Interior and Spatial Planning.<br>- The *Action plan for the promotion of healthy nutrition and physical activity* was issued by the Ministry of Education, the Ministry of Health, the Ministry of Family and Integration, and the Ministry of Sports.<br>- Since 2007, there has been a coordinating mechanism in the area of PA promotion. The leading institutions are the Ministry of Health and the Ministry of Sport. The participating stakeholders are: government departments on food, health, urban planning, education, sport, research, and social welfare; academia; communities; media; civil society; and NGOs.
World Health Organization, 2007 [227]	- A national interdisciplinary coordination body will be established to implement the action plan *Eat healthy, move more*. It will assess the aims, identify further needs, and perform regular modifications of the programme on PA and nutrition.<br>- After the first National Health Conference (held in 2005), the Ministry of Health took initiative to develop a national programme for the promotion of PA and healthy nutrition.<br>- Four ministries (education, sport, health, and family) are jointly working on developing a common policy and action plan related to PA and nutrition. Some of the key goals are to: increase the quality and quantity of PA at the population level and raise the awareness of a healthy lifestyle promoting mental, social, and physical health. Special focus will be put on children and youth.
Macao SAR, China	
Ramirez Varela et al., 2016 [32]	- No national/subnational PA plan.
Macedonia, FYR	
Ramirez Varela et al., 2016 [32]	- The PA plan entitled *Development of comprehensive plan for creating healthy municipalities through physical activity in urban environment* is available.
Kahlmeier et al., 2015 [127]	- National PA recommendations have not yet been developed.
Lachat et al., 2013 [140]	- The *Second action plan on food and nutrition in the Republic of Macedonia for 2009–2014* was issued in 2009.

	- The national policy includes the following targets and actions for PA promotion: enhance possibility of PA through its everyday life integration (e.g. workplace, school, and kindergartens); provide support to local authorities to remove barriers for active transportation and build recreational infrastructure; and ensure that "recommendations for proper nutrition are always followed by recommendations for PA".
World Health Organization, 2010 [229]	- Campaign *Health for All* was launched by the Government in 2007. It offered free counselling on PA and healthy diet and preventive health check-ups for those of 20+years of age .
Madagascar	
Lachat et al., 2013 [140]	- National policy target/action (details not specified) is to develop NCDs prevention policy that includes PA recommendations.
Malaysia	
Ramirez Varela et al., 2016 [32]	- No national/subnational PA plan.
Sharif et al., 2016 [193] [194]	- The assigned grade for the indicator *Government Strategies and Investments* from *RC on PA for Children and Youth* is B.<br>- There is no specific PA policy, but various strategies for PA promotion in children and adolescents are mentioned in the following policy documents:<br>a) The *National Sports Policy* (2009), with main objectives to develop sports culture, promote sort and PA participation through High Performance Sports, Sports for All, and Sports as an Industry. The policy mentions goals, strategies, responsibilities, and roles of all institutions involved in sport.<br>b) *One Student One Sport* (2011), targeting adolescents and school children. It is mandatory for students to participate in at least one sport systematically planned and organised at school.<br>Policy goals include, for example: sports culture development; self-esteem building; physical fitness improvement; fostering racial unity; and providing balance between physical fitness and academics.<br>- PA programmes/initiatives by the Ministry of health: *10,000 steps a day* (2009), *Want to be Healthy* (2013), and *Young Doctors* club (issued in 2006 in collaboration with the Ministry of Education).
Lachat et al., 2013 [140]	- The Malaysia Ministry of Health—National Coordinating Committee on Food and Nutrition issued the *National plan of action for nutrition of Malaysia (2006–2015).*<br>- National policy includes the following targets and actions for PA promotion: increase the ratio of persons engaged in minimum 30 minutes of PA per day, three times a week (in relation to the First Malaysian Food Consumption Survey); and promote physical fitness activities at the worksites for the whole population.
Aman, 2005 [66]	- *National Sports Council of Malaysia Act* (1971) had a goal to develop sport for the nation building purposes. The Government used a sport policy and sport funding to promote nationhood objectives, mainly the pursuit of harmonious multiculturalism.<br>- The central administrative body for the recreation and sport issues is the Ministry of Youth and Sport. Sport is considered to be just a sub-sector of youth policies, and leisure receives a secondary or tertiary focus by official administrative bodies. Some of the strategies of the Ministry to increase the proportion of people participating in healthy and active lifestyle are *Sport for All* and *Active Malaysia*.<br>- Initiatives, policies, or programmes related to leisure and sport include the following: *The National Sport Policy*, *The National Youth Policy*, and *National Fitness and Recreation Council* (issued by the Ministry of Youth and Sport); *National Park Act 1980* (issued by the Ministry of Science, Technology and Environment); *National Forestry Policy 1978* (issued by the Ministry of Forestry); *Town and Country Planning Act 1976*, *Development Plan Rules 1984* (issued by the Ministry of Housing and Local Government); and *Malaysia School Sports Council* (issued by the Ministry of Education).<br>- PE is mandatory in primary and secondary schools.<br>- Malaysian leisure policies are generally not research-based.<br>- Approach to sports policy is top-down and the government has a big, direct influence on recreation and sports organisations.
Maldives	
Ramirez Varela et al., 2016 [32]	- No national/subnational PA plan.

Malta	
Ramirez Varela et al., 2016 [32]	- PA is mentioned in the document entitled *A Strategy for the prevention of non-communicable diseases in Malta.*
Kahlmeier et al., 2015 [127]	- The national PA recommendations were published in the document entitled the *Dare to be active! Physical activity guide for youth,* issued in 2010.<br>- Specific recommendations are provided for children/young people and adults.<br>- The PA recommendations are not fully aligned with the WHO recommendations.<br>- The document includes recommendations on muscle-strengthening activities for children/young people.<br>- The document includes recommendations on SB for children/young people.
Christiansen et al., 2014 [44]<br>World Health Organization, 2011 [231]	- The document *Re-Shaping sport – towards personal development, health and success 2007–2010*, issued in 2007 by the Ministry of Education, Youth and Employment, mentioned that PA levels should be increased and that children should engage in at least one hour of PA daily.
World Health Organization, 2010 [12]	- The following national documents related to PA were identified: *A strategy for the prevention of non-communicable diseases in Malta* (2010); *Together for a sustainable future. Chapter 9. Investing in our children's education* (2009); *Reshaping sports – towards personal development, health and success. A medium-term strategic plan for enhancing sports culture in Malta 2007–2010* (2007); *Draft National Environment and Health Action Plan 2006 – 2010* (2006); and *Sports Act Chapter 455* (2003).<br>- Since 2003, there has been a coordinating mechanism in the area of PA promotion, and the leading institution has been the Health Promotion and Disease Prevention Directorate. The participating stakeholders are: government departments on education, health, youth, sport; local government; academia; and Malta Olympic Committee and Education.
World Health Organization, 2007 [227]	- The Malta Environment and Planning Authority, the Malta Transport Authority, and the Ministry of Health are represented within a transport and environmental committee. The committee has been established for promotion of safer transport, including active transport, such as cycling and walking to school.
Marshall Islands	
Ramirez Varela et al., 2016 [32]	- NCD plan includes PA (details are not specified).
Mauritius	
Lachat et al., 2013 [140]	- The Ministry of Health & Quality of Life—Nutrition Unit issued the *National plan of action for nutrition 2009–2010*.<br>- National policy includes the following targets and actions for PA promotion: stressing out in the dietary guidelines the maintenance of healthy weight by undertaking sufficient PA; cooperation of ministries of health and finance; and focus on the promotion of PA in elderly population.<br>- The policy included detailed actions and an implementation plan for stakeholders.<br>- The policy contained strategies for PA promotion that targeted educational institutions.
Mexico	
Galaviz et al., 2016 [106]	- The assigned grade for the indicator *Government strategies* from *RC on PA for Children and Youth* is C.<br>- New *National Strategy to Prevent Obesity and Diabetes* replaced the *National Food Health Strategy.*<br>- Under the *National Program for Physical Culture and Sports* two programmes were introduced: *Muevete en 30* (the promotion of PA in adults and children) and *Ponte al 100* (an assessment of children's functional capacity).<br>- The budget for PA promotion was $180 million in 2013 and $202 million Mexican pesos in 2014.<br>- Government initiatives related to active transportation included, for example: Sunday open streets for pedestrians and bicyclists; bicycle sharing programmes; public bicycle parking; pedestrisation of streets.<br>- Even though PA policy has been introduced in Mexico, a "leadership from the public health sector is lacking".
Pérez-Escamilla, 2016 [167]	- Interdisciplinary and intersectoral committee of eleven experts and eleven external advisors developed the *Mexican Dietary and Physical Activity Guidelines.* One of the recommendations relates to PA and mentions that in addition to routine daily activities

	it is necessary to engage in PA every day for 30 minutes or more (e.g. fast paced walking, playing, or dancing).
Pratt et al., 2016 [171]	- In Mexico, a policy drives PA research and not the other way around.
Ramirez Varela et al., 2016 [32]	- PA is mentioned in the document entitled *Ponte al 100: Checate, midete, muevete; Estrategia Nacional para la Prevencion y Control del Sobrepeso la Obeisidad y la Diabetes.*
Méndez, 2015 [153]<br>(in Spanish language)	- *General Law on Physical Culture and Sports* (2014) and *National Programme of Physical Culture and Sport* (2014) are the key documents for PA promotion.<br>- There is a lack of genuine implementation of PA public policy, but some separate actions are in place that contribute to the education and public health.<br>- Three key issues that could lead to the failure of PA promotion policy are: generality (a policy includes general objectives but lacks methods of evaluation and specific operative projects); institutional vision; and difficulty in defining proper distribution of responsibilities on different government levels.<br>- The public policy for PA promotion lacks concrete tasks to reduce people's sedentary lifestyles through the development of specific programmes for different target groups and strategies for rebuilding public spaces for sport practice.
Rodriguez Martinez et al., 2014 [183] [182]	- The assigned grade for the indicator *Government* from *RC on PA for Children and Youth* is "6" (C).<br>- Most of the PA policies were intersectoral and mostly created by the Ministry of Public Education and the Ministry of Health and their cooperation with the private sector.<br>- The *National Food Health Strategy* (2010) mentions specific strategies for PA promotion within schools.
Pilar Rodriguez et al., 2012 [170]	- The assigned grade for the indicator *Policy and programs* from *RC on PA for Children and Youth* is "7" (good), (B-).<br>- The main purpose of the *General Law on Physical Culture and Sports* and its Regulations (2003) are: the establishment of coordination between municipal and federal state governments and creation of agreements for inclusion of private and social sectors in matters related to sports and physical culture.<br>- National Physical Culture and Sports Commission is a leading body for sports at the national level that drafted *National Plan for Physical Culture and Sports* (2008).<br>- Programmes by the Ministry of Health that promote PA are *Five Steps for Your Health* and *The National Healthy Food Agreement: Strategy against Overweight and Obesity* (2010).
Micronesia, Fed. Sts.	
Ramirez Varela et al., 2016 [32]	- NCD plan includes PA (details are not specified).
Moldova	
Ramirez Varela et al., 2016 [32]	- PA is mentioned in the document entitled *National health policy: 2007 – 2021.*
Lachat et al., 2013 [140]	- The document entitled *National health policy: 2007–2021* is available.<br>- National policy includes the following targets and actions for PA promotion: expand special grounds for PA and urban public green space, for the whole population; enable wide communication at the population level for PA promotion; including sedentary population and elderly; enable revival of regular short brakes at workplace and in schools; and encourage PA through school curricula and school support.<br>- The policy mentioned the need for urban planning and sports infrastructure and mentioned specific strategies to address sedentary lifestyles.
World Health Organization, 2010 [229]	- Policy documents in the PA area are not yet available.
World Health Organization, 2007 [227]	- A plan to strengthen PA is in preparation and there is political will to increase the amount allocated resources in the budget for the sports facilities exist.
Monaco	
World Health Organization, 2010 [229]	- Policy documents in the PA area are not yet available.
Mongolia	
Ramirez Varela et al., 2016 [32]	- NCD plan includes PA (details are not specified).

Chimeddamba et al., 2015 [92]	- *The National Strategy on Healthy Diet and Physical Activity 2010–2021* was issued in 2009 by the Government of Mongolia. It aims to reduce mortality and morbidity caused by NCDs by creating supportive environments for PA at community, individual, national, and organisational levels.<br>- When the Mongolian NCD prevention policy was weighted against objectives of the *WHO 2008–2013 Action Plan for the Global Strategy for the Prevention and Control of NCDs* authors of the paper found gaps and negligence regarding the PA guidelines and PA promotion.<br>- Strengthening and reinforcing the existing national PA policies and action plans with additional strategies is recommended.
Lachat et al., 2013 [140]	- In 2007, the Ministry of Health issued the *National programme on prevention and control of noncommunicable diseases*.<br>- The national policy included the following targets and actions for PA promotion: create market incentives and tax measures to support PA promotion; enhance quality and accessibility of facilities and areas related to sport and improve their safety lighting; promote PA at the population level; develop and implement distance and informal learning training programme about PA; provide advice for the promotion of active movement and physical culture; design population specific standards and PA guidelines; and introduce elementary PA knowledge into secondary schools' curricula.<br>- The policy mentioned the need for urban planning and sports infrastructure, and contained strategies for PA promotion that targeted educational institutions.<br>- The policy included detailed actions and an implementation plan for stakeholders.
Montenegro	
Lachat et al., 2013 [140]	- In 2009, the Montenegro Ministry of Health issued the *Action plan for nutrition and food safety for Montenegro 2010–2014*.<br>- National policy includes the following targets and actions for PA promotion: create awareness in media; help local government in building safe paths for pedestrians and bicycle riders and designing models for PA facilities; local governments should enable conditions for the development of PA facilities (such as playgrounds, swimming pools, cycling and walking roads, parks) and infrastructure; develop PA programmes for universities, schools, and kindergartens; organise educational programmes in schools about the significance of PA; and develop conditions to support walking and cycling in traffic.
World Health Organization, 2010 [229]	- Policy documents in the PA area are not yet available.
Morocco	
World Health Organization, 2014 [232]	- There is a policy for healthy lifestyles promotion (details not specified).<br>- Legislation that mentions the requirements for the PE curriculum across different school grades was identified.<br>- Besides the general population, the population groups that are covered in national policy documents are: children and young people; older adults; workforce/employees; women; the clinical population with chronic diseases; sedentary/the most inactive; low socioeconomic groups; and families.<br>- Settings covered by national policy documents are: primary schools; high schools; colleges/universities; primary health care; clinical health care; workplace; sport and leisure; transport; environment; and urban design/planning.
Lachat et al., 2013 [140]	- In 2013, the Ministry of Health, United Nations Children's Fund issued *La strategie de la nutrition 2011–2019*.<br>- National policy includes the following targets and actions for PA promotion: by 2019, 80% of children and 70% of the general population should become active; and advocate for the environment and public space suitable for PA.<br>- The policy contained strategies for PA promotion that targeted educational institutions.
Mozambique	
Prista et al., 2016 [173]	- The assigned grade for the indicator *Government* from *RC on PA for Children and Youth* is F.<br>- Policies for PA and sport promotion exist, but they are not implemented or put into action.<br>- There is no plan or strategy for PA and sport promotion in children.<br>- There is a lack of data from authorities related to PA promotion.

Ramirez Varela et al., 2016 [32]	- No national/subnational PA plan.
Prista et al., 2014 [174]	- The assigned grade for the indicator *Policy* from *RC on PA for Children and Youth* is C.<br>- There are several government regulations and laws on the promotion of participation in sport and for PA promotion, but they refer less to PA in general than to formal sport.<br>- Three main policy documents for PA promotion in adolescents and children are: *Strategic Plan for Education from 2012 to 2016*; the *General Regulation for Physical Activity and Sports*; and *Decree Law for Scholar Sports Regulation*.<br>- Policies did not create the necessary conditions for proper PA promotion, but they have established an official stand that recognises the importance of PA in youth development and public health in general.
Myanmar	
Ramirez Varela et al., 2016 [32]	- No national/subnational PA plan.
Nepal	
Ramirez Varela et al., 2016 [32]	- No national/subnational PA plan.
Netherlands	
Coenen et al., 2017 [38]	- *Physical Activity Guidelines* (Ministry of Health, Welfare and Sport, 2014) do not include recommendations on SB.
Burghard et al., 2016 [87] [88]	- The indicator *Government Initiatives* from *RC on PA for Children and Youth* did not receive a grade, it was marked as incomplete, because of unclear benchmark/criteria.<br>- The Ministry of Health, Welfare, and Sports published the *National nota health policy. Health near by* (2011). The Ministry has been providing the resources to stimulate activities such as: exercise close to home; customised and reliable information; and accessible opportunities. The budget for this policy declined over time; it was €64 million, €59 million, €53 million, and €48 million in 2011, 2012, 2013, and 2014, respectively.<br>- There is no national norm related to SB.<br>- Three key policy vision points by the Government are: the Government will help people to protect their health with laws, regulations and a surveillance system; sport and health care should be easily accessible and close to home for everyone; and the Government would not like to dictate people's lifestyle choice, as it is a person's own decision to engage in certain activities or not.
Hämäläinen et al., 2016 [115]	- Relevant HEPA national policy documents include: *Health close People 2012–16*, *Sports & Physical Activity in the Neighbourhood 2012–16*; and national/local - *Youth on healthy weight 2010–15*.<br>- HEPA policies cooperation and coordination processes and structures include: government/regional/local committees or working groups with cross-sector representatives; contacts between public sector officers responsible for HEPA between levels; established systems of policymaking; steering committees; private sector involvement in policymaking; and formal consultation on HEPA policy for stakeholders.<br>- There are no: field visits to make a policy; public hearings for citizens; national/regional/local politically elected councils; intersectoral committees or working groups for HEPA; scientific advisory groups/institutes/individuals; and administrative working groups including only public sector officers.
Ramirez Varela et al., 2016 [32]	- A national or subnational PA plan is available (details are not specified).
Kahlmeier et al., 2015 [127]	- The national PA recommendations were published in the document entitled *The power of sport,* issued in 2005.<br>- Specific recommendations are provided for children/young people, adults, and older adults.<br>- The PA recommendations are not fully aligned with the WHO recommendations.<br>- The document includes recommendations on muscle-strengthening activities for children/young people.<br>- The document does not include recommendations on SB.
Stuji & Stokvis, 2015 [200]	- Involvement in sport by the Government intensified by the end of 1960s. In 1969, a *Discussion document concerning sport policy* was issued by the Minister of Culture, Recreation, and Social Work and stated that sufficient amount of PA is important for

	good physical health, which was the first-time health was linked with sport in a policy document in the Netherlands.<br>- In 1983, *Memo accents sport policy 1984 and further* was issued by the Ministry of Welfare, Public Health, and Culture. It contained more precise information on the relationship between sport and certain aspects of health. It also noted that only competitive and elite sports were considered as sport, whilst this concept is now much broader and also includes recreation.<br>- In 2001, the document *Sport, exercise and health,* issued by the same Ministry, for the first time included scientific references to support stated objectives.
Bull et al., 2014 [85] [83] [84]	- The Ministry of Health, Welfare, and Sport is the responsible body for PA.<br>- Key documents related to PA include: *Excellence at Every Level* (2009); *The power of Sport* (2008); *The Sport, Physical Activity and Education Policy (2008)*; *Being Healthy and Staying Healthy: A Vision of Health and Prevention* (2007); *Together for Sport* (2006); *Opting for a healthy life, Public Health policy in the Netherlands 2007-2010* (2006); *Time for Sport* (2005); and *Dutch Bicycle Master Plan* (1999).<br>- The document *Agenda for a living countryside: multi-year programme for a living countryside 2007-2013* puts a strong emphasis on walking.<br>- PA is not a Government's priority, but there is a significant political commitment to PA promotion.
Christiansen et al., 2014 [44]<br>World Health Organization, 2011 [231]	- The policy document *Time for sport: exercise, participate, perform* was issued in 2005. It was complemented (but not replaced) by the new policy document *The power of sport*, issued in 2008. Both documents were issued by the Ministry of Health. In this study, the two policy documents were referred to as a single strategy.<br>- The strategy includes the following quantifiable targets: by 2012, at least 70% of adults should engage in the recommended amount of exercise; and, by 2012, there should be less than 5% of inactive people.<br>- The strategy also mentioned the following vulnerable groups with low PA levels: chronically ill people and people with disabilities; elderly; residents of deprived areas; and immigrants. It is also stated that sport and PA are especially important for their social integration and health.
Kalman & Hamrik, 2013 [128]<br>(in Czech language)	- In a policy document (name not specified) related to PA the following target groups are specifically addressed: chronically ill people; people with disabilities; elderly; youth; and people with professional difficulties.
Ceccarelli et al., 2011 [90]	- The quantifiable targets mentioned in the analysed policy document (details not specified) are: by 2010, at least 65% of adults should meet the exercise standards, compared to 60% in 2004; the number of young people (12-17 y.o.) meeting the exercise standards should increase to 40% compared to 35% in 2004; and 90% of young people should have the opportunity to participate in sport after school or in school, five times a week.<br>- The stakeholders related to PA and sport mentioned in the policy document are: sports goods manufacturers; media; and advertising and recreation businesses.
World Health Organization, 2010 [12]	- The following national documents related to PA were identified: *Memorandum on obesity – out of balance: the burden of obesity* (2010); *National Action Plan for Sport and Exercise* (2006); *Sport action plan against obesity* (2005); *Time for sport: exercise, participate, Perform* (2005); *Covenant on overweight and obesity, a balance between eating and physical activity* (2005); *National Plan of Action for Children 2004*, *Living longer in good health, also a question of a healthy lifestyle* (2004); and *Towards an active policy* (2003).<br>- Since 2003, there has been a coordinating mechanism in the area of PA promotion and the leading institution has been the National Institute for Sports and Physical Activity. The participating stakeholders are: government departments on sport, food, research, education, transport and labour; communities; and academia.
Daugbjerg et al., 2009 [11]	- The policy document *Time For Sport—Exercise, Participate, Perform* (2005) contains quantifiable PA goals and a budget for policy implementation. It does not contain a requirement/intention for evaluation. From the three components mentioned, the document *National Plan of Action for Children* (2004) contains only a requirement/intention for evaluation.<br>- The document *Living longer in good health—also a question of a healthy lifestyle* (2004) has the time frame from 2004 until 2007 and it contains budget and a requirement/intention for evaluation, but it does not contain quantifiable PA goals.

	- The document *Toward an 'active' policy* (2003) does not contain either of the three components.
Branca et al., 2007 [80]	- The document *Living longer in good health: also a question of healthy lifestyle. Netherlands Health-Care Prevention Policy* (2004) stated that in 2004 the aim was to spend €45 billion on health care, including €625 million for health protection and promotion. It was issued by the Ministry of Health, Welfare and Sport.
World Health Organization, 2007 [227]	- Policy documents related to PA are: *Sport action plan against obesity* (2005), *Towards an active policy* (2003) and *Time for sport* (2005).<br>- *Time for sport* pays special attention to HEPA. The main target for the year 2010 is to increase the percentage of population engaged in PA at least 30 minutes/day or the number of people in the population who play sports at least three times a week, by 5%.
Bull et al., 2004 [26]<br>Schöppe et al., 2004 [187]<br>Bull et al. in Bull et al., 2004 [86]	- The following documents related to PA promotion were identified: *What sport sets in motion. Contours and priorities of central government policy on sport* (1996); *Sport and Sports Policy in the Netherlands* (1997); *Opportunities for Top-Class Sport. The Government's Policy on Top-Class Sport* (1999); *Sport for All incentive in the Netherlands* (2000); and *Towards an 'active' policy* (2003). All the documents were issued by the Ministry of Health, Welfare and Sport. The focus of the last document was placed on the relationship between exercise, sport, and health. Some of the quantifiable targets are: increase the prevalence of PA to 45% in 2005 and to 50% in 2010 (compared to 40% in 1998); and decrease the prevalence of physical inactivity to 10% in 2005 and to 8% in 2010 (compared to 12% in 1998).
Van Mechelen in Simonopoulos (ed.), 1997 [210]	- There is a national policy for the promotion of PA entitled *Netherlands on the Move* that aims at the following target groups: children; elderly; working population; and chronically ill people. The policy is endorsed and financed by the Government.
New Caledonia	
Ramirez Varela et al., 2016 [32]	- NCD plan includes PA (details are not specified).
New Zealand	
Coenen et al., 2017 [38]	- Measures to reduce occupational SB included in the *Guidelines for using computers - Preventing and managing discomfort, pain and injury*, issued by the New Zealand Government in 2010, specify how often and for how long brakes should be taken in different working contexts.
Maddison et al., 2016 [149]<br>Maddison et al., 2015 [148]	- The assigned grade for the indicator *Government Initiatives* from *RC on PA for Children and Youth* is B-.<br>- The PA guidelines for children and youth state that children and youth (5 to 18 y.o.) should accumulate 60 minutes or more of MVPA per day and recommend to spend less than two hours daily in front of the computer, television, and gaming consoles.<br>- There is no national-level framework for PA promotion.<br>- There are no clear strategies for evaluation.<br>- The *Childhood Obesity Plan*, published by the Government in 2015, is focused on PA, environment, and nutrition.<br>- Some examples of actions and initiatives include: *Guidelines for Sustainable Physical Activity in School Communities*; *Good Practice Principles for the Provision of Sport and Recreation for Young People*; *ActiveSmart*; *BikeWise*; and *Sport in Education*.
Ramirez Varela et al., 2016 [32]	- NCD plan includes PA (details are not specified).
Maddison et al., 2014 [147]	- The indicator *Government Initiatives* from *RC on PA for Children and Youth* did not receive grade. It was marked as incomplete.<br>- The following sector-based and government-led strategies, policies, and funding programmes were identified: sport and recreation (e.g. *Kiwisport Partnership Fund*, *ActivePost*); transport (e.g. model communities and *BikeWise*); education (e.g. *Health and PE curriculum*, *Kiwisport school funding*, *Sport in Education*); and injury prevention and health (e.g. *Physical Activity Guidelines*, *Health Promoting Schools*).
Brown et al., 2011 [43]	- National documents related to PA are: *Health of Older People Strategy* (Ministry of Health, 2002); Physical Activity (Ministry of Sport Fitness and Leisure, 1999); and *New Zealand Physical Activity Guidelines* (Hillary Commission 2001). The guidelines were based on the *Surgeon General's Report* (USA, 1996).
Ceccarelli et al., 2011 [90]	- The identified goals in policies that deal with nutrition, PA, and obesity are general

	and not quantifiable and measurable.<br>- The analysed policy document (details not specified) explicitly refers to the international assessment of the obesity problem or to the *WHO Global Strategy on Diet, PA and Health.*
Gillon, 2010 [107]	- Sport and Recreation New Zealand (SPARC) is a government organisation responsible for the promotion and monitoring of PA.<br>- SPARC issued a *Strategic Plan 2009-2015* and listed government priorities related to recreation and sport, including: improvement of resources for high performance sport; enhancing school-based initiatives; strengthening of grassroots sports delivery; and improving accessibility of opportunities related to physical recreation.<br>- *Sport and Recreation Act 2002* balances between the importance of PA and sport and emphasises the importance of participation of all people in sport and recreation to achieve wellbeing and health. It mentions the following specific target groups and their inclusion and active participation in recreation: Maori; women; Pacific peoples; people with disabilities; and elderly.
Piggin, 2008 [168]	- The report *Getting set for an active nation* (informally called *Graham Report*) by the Ministerial Taskforce made a number of recommendations for the national recreation and sport policy such as: increasing the school day for 30 min per day for an obligatory PE session (for 5-12 y.o. children); replacing the Hillary Commission for Sport, Fitness and Leisure with a new organisation; and better rationalisation of elite sport.<br>- In 2002, Hillary Commission was replaced by Sport and Recreation New Zealand (SPARC).<br>- Throughout time, SPARC was perceived as, for example: an "omnipotent state monolith"; an organisation of individuals that are doing their best to encourage people to engage in PA; and an arrogant organisation that aimed at reinforcing masculine historical understanding of sport.
Aman, 2005 [66]	- *The Physical Welfare and Recreation Act* (1937) was the first leisure-related legislation. It aimed to: raise the standard of fitness and health; encourage active participation in recreation activities; enhance formation of youth clubs; and provide training to leaders of various organisations.<br>- *The Recreation and Sport Act* 1973 had a much wider goal than just sport promotion. It aimed to improve mental and physical health of the population.<br>- *Recreation and Sport Act* 1987 mentioned disestablishment of the Ministry and Council for Recreation and Sport. The Council was replaced by the Hillary Commission for Recreation and Sport ("quasi-autonomous non-government organisation"). The establishment of the Comission was the way towards "depoilitisation" of recreation and sport.<br>- *The Sport, Fitness and Leisure Amendment Act* 1992 was mainly focused on achieving exercise and fitness through involvement in sport.<br>- New Zealand has no comprehensive, formal leisure policy.
Bull et al., 2004 [26]<br>Schöppe et al., 2004 [187]<br>Bull et al. in Bull et al., 2004 [86]	- National Physical Activity Taskforce was established in 1998, with a goal to address the problem of physical inactivity.<br>- The document *Getting set - For an Active Nation* was issued in 2001. It provided a 25-year vision for increasing PA levels. In the same year the *Guidelines for Promoting Physical Activity (movement = health!)* were issued by the Hillary Commission.<br>- In 2003, SPARC produced a document *Towards an Active New Zealand – Developing a National Policy Framework for Physical Activity and Sport*, stating that effective strategies have to be provided for the: whānau; people with disabilities; Pacific people; hapu; women; iwi; and older adults. SPARC's policies and programmes were funded by the New Zealand Lottery Grants and the Government. The financial support varied from $50 million (in 2002/03) to $70 million (by 2005/06).
Nicaragua	
Ramirez Varela et al., 2016 [32]	- No national/subnational PA plan.
Niger	
Lachat et al., 2013 [140]	- The document *Politique nationale en matiere d'alimentation et de nutrition* was issued in 2006.<br>- The national policy target/action for PA promotion is to promote a healthy lifestyle in communities and families to enable relaxation and sport to become widespread within the local surroundings.

Nigeria	
Ramirez Varela et al., 2016 [32]	- No national/subnational PA plan.
Adeniyi et al., 2016 [59]	- The assigned grade for the indicator *Government, Nongovernmental Organizations, and Private Sector (Strategies and Investments)/Policy* from *RC on PA for Children and Youth* is B.<br>- There are school policies for teaching health and PE. They contain a provision that mentions suitable environment for youth and children to participate in sport activities.<br>- In 2004, the Federal Ministry of Education issued a *National Policy on Education.* The document includes policy on PE and PA.
Akinoroye et al., 2014 [61]	- The indicator *Government Strategies and Investment* from *RC on PA for Children and Youth* did not receive a grade. It was marked as incomplete.<br>- Government policy on sports exists, but there is no further information on implementation and investments related to the policy. According to the policy, the Schools Sports Federation and the Nigeria Academicals Sports Committee were legally obliged to cooperate with the local and state governments in sports promotion in schools. However, no evidence is available related to the effectiveness of this policy or regarding any other policy objectives.
Northern Ireland	
Coenen et al., 2017 [38]	- In the publication *Start active, stay active - A report on physical activity for health from the four home countries' chief medical officers*, issued by the Government's Department of Health in 2011, it is stated that "all adults should minimise the amount of time spent being sedentary (sitting) for extended periods".
Harrington et al., 2016 [120] [121]	- The indicator *Government* from *RC on PA for Children and Youth* did not receive a grade. It was marked as incomplete.<br>- The reasons for not assigning a grade are the gaps in national level surveillance of PA in children and uncertainty whether investments in this area exist or not.<br>- The following documents that include targets for children's PA were identified: *A Fitter Future for All Obesity Action Plan 2012-2022* and *Sport Matters: the Northern Ireland Strategy for Sport & Physical Recreation 2009 – 2019*.<br>- Minimisation of the time spent in SB has been recommended within the guidelines *Start Active, Stay Active: A Report on Physical Activity for Health from the Four Home Countries* issued by the United Kingdom Chief Medical Officers.<br>- The following documents related to active travel were identified: *An Action Plan for Active Travel in Northern Ireland 2012 – 2015* and the *Northern Ireland Changing Gear – A Bicycle Strategy for Northern Ireland 2015.*
Ramirez Varela et al., 2016 [32]	- PA is mentioned in the documents entitled *Obesity Framework for Northern Ireland 2012-2020, Sport Matters* and *The Northern Ireland Strategy for Development of Sport and Physical recreation 2009-2019.*
Kahlmeier et al., 2015 [127]	- The national PA recommendations were published in the document entitled *Physical Activity, Health Improvement and Protection. Start Active. Stay Active: A report on physical activity from the four home countries',* issued in 2011.<br>- Specific recommendations are provided for children/young people, adults, and older adults.<br>- The PA recommendations for children/young people and adults are fully aligned with the WHO recommendations.<br>- The document includes recommendations on muscle-strengthening activities for adults and older adults and recommendations on SB for children/young people, adults, and older adults.
Christiansen et al., 2014 [44]<br>World Health Organization, 2011 [231]	- The following documents related to sport promotion were issued in 2009: *Sport matters: a culture of lifelong enjoyment and success in sport 2009–2019,* and the action plan *Sport Northern Ireland, Corporate plan 2008–2011.* In this study, the two policy documents were referred to as a single strategy<br>- The strategy mentions PA-related aims such as: ensure that 90% of the population has access to sport facilities by 2019 (within twenty minutes travel time); and increase participation of adults in recreation and sport by at least 3% compared to the 2011 baseline.
Harrington et al., 2014 [118] [119]	- the indicator *Government* from *RC on PA for Children and Youth* did not receive a grade. It was marked as incomplete, because national PA plan and health surveillance system were lacking.

Pate et al., 2011 [37]	- Policy related to PE in schools states that: children should engage in at least two hours of PE a week; PE should be taught by highly qualified and certified teachers; and PE curriculum should be included in the school review process.<br>- PA policy within the area related to community environmental support states that: access to public sports facilities and venues should be free or offered at discounted prices for students; parks, playgrounds, and open spaces that are interesting and challenging for youth and children should be created; community organisations and local authorities should be support, funded, and encouraged to develop PA promotion programmes for families to help them become active and use the existing infrastructure; and cooperation between colleges, youth clubs, and schools with community groups, local authorities, and health professionals should be encouraged to design programmes to increase involvement in PA.<br>- PA policy in the area related to school environmental support states that: access to a broad range of activities such as dance, sports, active travel, play, exercise and being active in daily tasks should be provided around and in school; schools should be encouraged to conduct fitness tests of their students annually and store the collected data; parks around schools and school playgrounds should be designed and renovated to inspire sport, movement, play, and outdoor education; awards should be given to schools for promoting holistic health in the school settings, including the promotion of PA in students, parents, and staff.<br>- PA policy within area related to active transport/urban design states that: schools and education boards should collaborate with Department of the Environment to provide safe routes to schools; designated car drop off zones half kilometre from schools should be established and walking from the zones to school should be organised and supported.<br>- The data were extracted from the following documents: *Physical Activity: An Investment in Public Health* and *The Northern Ireland Physical Activity Strategy Action Plan 1998–2002*., issued in 1997 by the Health Promotion Agency for Northern Ireland.
World Health Organization, 2010 [12]	- The following national documents related to PA were identified: *Walking Northern Ireland, an Action Plan* (2003); *Northern Ireland's road safety strategy 2002–2012* (2002); *Northern Ireland Cycling Strategy* (2000); and *The Northern Ireland Physical Activity Strategy Action Plan 1998–2002* (1998).
Bornstein et al., 2009 [79]	- In the document *Physical Activity: An Investment in Public Health: The Northern Ireland Physical Activity Strategy Action Plan 1998-2002* some of the targeted population groups are: people older than 50; young mothers and young people in general; unemployed; and young people of school age (with female teenagers as a special subgroup).<br>- The document provides a detailed accountability chart for all bodies that participate in carrying out the objectives of the action plan. Some of the mentioned goals to be achieved by 2002 were to: reduce the number of people (older than 16) who are classified as sedentary from 20% to 15%; and increase the number of people who achieve recommended PA levels from 30% to 35% among men and from 20% to 25% among women.
Musingarimi, 2009 [158]<br>Musingarimi, 2008 [157]	- The document *The Northern Ireland Physical Activity Strategy Action Plan 1998-2002* provides a framework for policy development and implementation of PA programmes.<br>- The document *Fit Futures: Focus on Food, Activity and Young people* aims to tackle obesity and overweight in children and youth by identifying opportunities that support active living and healthy eating.
Northern Mariana Islands	
Ramirez Varela et al., 2016 [32]	- NCD plan includes PA (details are not specified).
Norway	
Ramirez Varela et al., 2016 [32]	- The PA plan entitled *The Action Plan on Physical Activity 2005-2009. Working Together for Physical Activity. 8-2-0005* is available.
Kahlmeier et al., 2015 [127]	- The national PA recommendations were published in the document entitled *Physical activity. In: Nordic Nutrition Recommendations 2004 Integrating nutrition and physical activity,* issued in 2004 by the Nordic Council of Ministers.<br>- Specific recommendations are provided for children/young people and adults.<br>- The PA recommendations are not fully aligned with the WHO recommendations.<br>- The document includes recommendations on muscle-strengthening activities for

	children/young people.<br>- The document does not include recommendations on SB.
Bull et al., 2014 [85] [83] [84]	- The following documents related to PA promotion were identified: *Outdoor Recreation Act*, *The Planning and Building Act* (2009); *White Paper No.39 Outdoor recreation (Friluftsliv) - A way to better the quality of life* (2001); *Government's Environmental Policy and the State of the Environment in Norway* (2005); *White Paper No. 14 to the Storting (1999) Sport in a State of Change - About the State's relationship to sport and physical.*<br>- *The Action Plan on Physical Activity 2005-2009* is a product of the intersectoral work of eight ministries. It contains 108 measures for increasing PA and was evaluated.<br>- The environment sector is mentioned as a key area for PA promotion.
Kalman & Hamrik, 2013 [128]<br>(in Czech language)	- In the policy document (details not specified) related to PA it is mentioned that the "recipe" for a healthy Norway is in emphasising the importance of PA, health, and well-being for the whole population.
Brown et al., 2011 [43]	- The national document the *Action plan on physical activity 2005–2009 – Working together for physical activity* was issued in 2005 by the Norwegian Government.
Ceccarelli et al., 2011 [90]	- *Health, food and physical activity: Nordic Plan of Action on better health and quality of life through diet and physical activity* was issued by the Nordic Council of Ministers in 2006.<br>- The document includes a thorough analysis of PA and eating habits of the population.
Pate et al., 2011 [37]	- PA policy measures were found in the following areas: a) PE in school (e.g. requirement of at least two hours of weekly PE for all children that should be taught by highly qualified and certified teachers; b) health education (e.g. enable training for healthcare professionals in schools in motivational interviewing techniques related to PA, establishment of cooperation with universities to provide teacher education classes for inclusion of the *PA and Health* topic into the curriculum); c) community environmental support (e.g. support, fund, and encourage community organisations and local authorities to develop PA promotion programmes for families to get them active and use the existing infrastructure); d) school environmental support (e.g. provide access to a broad range of activities such as dance, sports, active travel, play, exercise, and being active in daily tasks around and in schools); e) active transport/urban design (e.g. a car drop off zones half kilometre from schools and support it by organised walking to school from the zone); and f) mass media/advertising campaigns (e.g. support a comprehensive, community wide PA campaign that provides opportunities and education to children and families in schools, communities and neighbourhoods).<br>- The data were extracted from the *Action Plan on Physical Activity 2005-2010*, issued in 2005 by the Ministry of Health and Care Services.
Skille & Sobakken, 2011 [195]	- In 1946, the Department of Sport Policy was established. Its work had been guided by the "Sport for All" vision which was a part of a greater idea to (re)build the welfare state. In contemporary Norway, this institution, along with the Norwegian Olympic and Paralympic Committees and the Confederation of Sport (NOC), is in charge for achieving the overall "Sport for All" vision. The Government has the responsibility to accomplish this goal, which can be achieved by sharing the work between voluntary and public institutions.<br>- All white papers related to sport in 1970s and 1980s were a part of White Papers on culture because sport is defined as culture, because of its associated intrinsic values, such as mastery, achievement, and joy. The White Papers published from 1990s (St. meld. no. 41, 1991–1992 and St. meld. no. 14, 1999–2000) were exclusively on sport.<br>- The White Paper *Prescription for a healthier Norway* (Government, 2002–2003), states that the goal of health policy is to "treat less and prevent more". It mentioned PA as one of five key concepts.<br>- Documents published by NOC contain "lofty formulations". Measurable and quantifiable goals are only related to elite sport (*Idrettspolitisk dokument 2003–2007*, *Idrettspolitisk dokument 2007–2011*).
World Health Organization, 2010 [230]	- The National Board of Nutrition and Physical Activity was established in 1999 when national policy started to focus on PA. In 2002, the Board split into a board for PA and a board for nutrition.<br>- In 2003, the Ministry of Health published the *Prescription for a healthier Norway* (a white paper). The document mentioned PA as one of the five most significant public

	health areas for the next ten years.<br>- In 2003, the Ministry of Education published *Culture for learning* a white paper that showed a focus on PA among education authorities.
Bornstein et al., 2009 [79]	- In the *Action Plan on Physical Activity 2005-2009: Working Together for Physical Activity,* the timeline for achieving goals was not identified. Key aims of the plan are to increase the proportion of youth and children who engage in PA for at least 60 minutes per day and the proportion of elderly and adults who are moderately active for at least 30 minutes per day.
Daugbjerg et al., 2009 [11]	- The policy documents *National Report on Youth Policy in Norway* (2004), *The Government's Environmental Policy and the State of the Environment in Norway* (published in 2005, time frame until 2007), and *Prescriptions for a Healthier Norway. A broad policy for public health—short version* (published in 2003, ten-year time frame) do not contain quantifiable PA goals, budget for policy and a requirement/intention for evaluation. From the three components, the document *Working together for physical activity. The Action Plan on Physical Activity 2005-2009* (2005) contains only a requirement/intention for evaluation.
Bergsgard et al., 2007 [76]	- "Sport for All" has been continuously recognised in Government's policy documents as the key objective of the sport policy and responsibility for its development is at the national level. The Government supports "Sport for All" by providing support for facilities and activity programmes. In the past 10-15 years, sport has received increasing financial support, almost completely from the lottery. The Ministry for Culture and Church Affairs is responsible for all national sport policies, concerning both elite and mass sport.
Branca et al., 2007 [80]	- *Working together for physical activity. The Action Plan on Physical Activity 2005–2009* (2006) proposed to include a provision in the legislation *Working Environment Act* that obliges employers to consider PA as part of company's support of safe and healthy work environments.
World Health Organization, 2007 [227]	- In 2005, the Parliament adopted the *Action plan for physical activity 2005–2009*, which is the result of a joint effort of 8 ministries. It comprises of 108 measures across different areas such as schools, workplaces, leisure activities, kindergartens, urban planning, and transport.<br>- A communication strategy for 2005–2009 was created to increase the knowledge about health and PA and to motivate people to adopt active lifestyles.
Oman	
Ramirez Varela et al., 2016 [32]	- PA is mentioned in the document entitled *Oman National Policy for the Prevention and Control of NCDs 2015-2025.*
Al-Bahlani & Marby, 2014 [62]	- The Ministry of Manpower issued a regulation *Occupation Safety and Health Organisational Regulation in the Institutions Subject to the Labour law* (2008) for promoting PA at the workplace.<br>- The *Sports Strategy* issued by the Ministry of Sports Affairs for promoting sports<br>- A policy for PE curriculum was issued by the Ministry of Education (details are not specified).<br>- Two documents that include PA recommendations were issued by the Ministry of Health (one document is entitled *Omani Guide to Healthy Eating*, whilst the name of the second document is not specified).
World Health Organization, 2014 [232]	- There is a national strategy or policy addressing PA and diet/nutrition (details not specified).<br>- It has been reported that national PA recommendations have been developed.<br>- The National Olympic Committee, under the supervision of the Ministry of Sports, is providing leadership in PA promotion.<br>- Besides the general population, the population groups that are covered in national policy documents are: children and young people; workforce/employees; the clinical population with chronic diseases; and sedentary/the most inactive people.<br>- The settings covered by national policy documents are: primary schools; high schools; primary health care; workplace; sport and leisure; transport; environment; and urban design/planning.
Pakistan	
Nishtar et al., 2006 [160]	- The *National Action Plan on NCD Prevention, Control, and Health Promotion* was released in May 2004. It was developed through joint efforts of the Ministry of Health, an NGO focused on prevention of chronic diseases *Heartfile* (an Islamabad, Pakistan-

	based non-profit organisation), and the WHO. They also jointly funded the first implementation phase of the Plan (May 2004 – July 2006). This phase had many action items and three priority areas related to the research, institutional mechanisms, and policies and legislation. One of the action items under *policies and legislation* area was the development of PA policy.
Palau	
Ramirez Varela et al., 2016 [32]	- NCD plan includes PA (details are not specified).
Papua New Guinea	
Ramirez Varela et al., 2016 [32]	- NCD plan includes PA (details are not specified).
Paraguay	
Ramirez Varela et al., 2016 [32]	- A national or subnational PA plan is available (details are not specified).
Peru	
Ramirez Varela et al., 2016 [32]	- No national/subnational PA plan.
Philippines	
Lachat et al., 2013 [140]	- In 2011, the Philippines Department of Health issued the *National policy on strengthening the prevention and control of chronic lifestyle related non communicable disease*s.<br>- National policy includes the following targets and actions for PA promotion: regulate built environment for PA promotion; implement and develop health promotion activities for PA; reduce the prevalence of "high physical inactivity" in adults from 60.5% to 50.8%.<br>- The policy mentioned specific strategies to address sedentary lifestyles.
Poland	
Ramirez Varela et al., 2016 [32]	- PA is mentioned in the document entitled *National Health Programme 2007 – 2015.*
Zembura et al., 2016 [225]	- The assigned grade for the indicator *Government—Strategies, Policies, Investments* from *RC on PA for Children and Youth* is C.<br>- The Government showed increased commitment and strategic interest for PA promotion.<br>- The Ministry of Sport and Tourism has undertaken four national interventions related to PA under the *Sport of All Children Programme*, but their reach was considered relatively low as they included only 6-8% of children and youth.<br>- Existing programmes are regularly evaluated and recognised by the local community and authorities.
Kahlmeier et al., 2015 [127]	- National PA recommendations are currently in the development process (the documents published until summer 2012 were reviewed).
Christiansen et al., 2014 [44]<br>World Health Organization, 2011 [231]	- In 2007, the policy document related to sport promotion entitled *Strategy of sport development in Poland to 2015* [*Strategia Rozwoju Sportu W Polsce Do Roku 2015*] was published. It has a goal to improve sports infrastructure. It mentions the needs of people with disabilities and the importance of balancing discrepancies in the availability of sports infrastructures between country's regions. It states that all people should have equal access to sport and PA opportunities, including those from the deprived and rural areas.
World Health Organization, 2010 [230]	- The central objective of the *Strategy of sport development until 2015* (Ministry of Sport and Tourism) is achieving "active and fit society". The Strategy aims to be implemented in the following priority areas: 'Sport for All'; improvement in sport achievements; and the development of sports and recreational infrastructure. Some of the key goals are: improvement of physical fitness in children and youth; reduction of the number of obese individuals; reduction of the number of people with a sedentary lifestyle; development of "active ways" for spending free time; and reduction of morbidity among youth.<br>- Physical Culture Development Fund is responsible for funding further development of sports infrastructure.
World Health Organization, 2010 [229]	- The *National programme for the obesity, overweight, and NCD prevention through diet and improved PA 2007−2011 (POL-HEALTH*) was approved by the Minister of

	Health and developed in 2007 by the National Food and Nutrition Institute. Activities within the programme mainly focus on increasing the awareness about the importance of PA and healthy diet and at providing comprehensive information and education to consumers.
World Health Organization, 2010 [12]	- The following national documents related to PA were identified: *National Health Programme 2007–2015*, issued in2007, and *National prevention programme of overweight, obesity and noncommunicable diseases through diet, and physical activity improvement 2007–2016*, issued in 2006.<br>- There has been a coordinating mechanism in the area of PA promotion, and the leading institution has been the Ministry of Sport and Tourism, the “Sport for All” Department. The participating stakeholders are: government departments on health, sport, and education; NGOs; academia; communities; and research institutes.
World Health Organization, 2007 [227]	- Some of the key objectives of the *National Programme for the Prevention of Overweight, Obesity and NCD through Diet and Improved Physical Activity 2007–2016* are to increase PA and improve nutrition habits in order to reduce the prevalence of obesity and overweight.
Portugal	
Mota et al., 2016 [155]	- The assigned grade for the indicator *Government* from *RC on PA for Children and Youth* is C.<br>- The *National Sports for All Programme* aims at providing strong sport base and better quality of life to all citizens. It highlights the importance of sport for enhancing social cohesion.<br>- Sport policies are integrated in the *Portuguese National Health Plan*, issued by the Ministry of Health, with the time frame until 2010.<br>- The School Sport Council established that organising sport activities is obligatory for all schools.
Ramirez Varela et al., 2016 [32]	- PA is mentioned in the document entitled *Health national plan.*
Kahlmeier et al., 2015 [127]	- National PA recommendations have not yet been developed.
Bull et al., 2014 [85] [83] [84]	- The Portuguese Sports Institute is the most important institution responsible for sport public policy.<br>- Key legislation including *Law No. 5 / 2007 of January 16 - Law on Physical Activity and Sport established the legal base* and *Law No. 46/86 of 14 October - Law of the Education* states that PE is obligatory for primary and secondary school children.<br>- There are no national PA recommendations.
Rütten et al., 2013 [185]	- Public policies related to LTPA include: *More sports, better quality of life* (2009); *National programme of walking and running* (2009); *National Sports for all Programme - MexaSe* (2005); *National Health Plan 2004-2010*; and *National Programme against obesity* (2004).<br>- Supportive environments for LTPA (indoor/outdoor sport facilities and infrastructure for LTPA) are broadly available. Green space, usable for LTPA, is partially available.
Costa Januario et al., 2012 [94]<br>(in Portuguese language)	- In 2002, the Government’s Programme (*Programa do Governo Constitutional*) declared that Portugal was incapable of fulfilling its mission towards sport. Until 2004, no precise goals for the promotion of PA and sport were established by the Government. From 2005, more focused goals were mentioned, for example creating the national “Sport for All” programme by engaging public-private partnerships. Goals from 2005 were reinforced in 2009 and the new programme also focused on more specific targets such as women in sport, elderly, and obligatory PE in schools.<br>- The Government emphasised that participation in sports is the right of every citizen. However, mentioned goals are vague and not precise enough in order to fulfil this right. Besides, no clear idea was identified on how sport should be developed within the framework of local authorities.
Ceccarelli et al., 2011 [90]	- The identified goals in policies that deal with nutrition, PA, and obesity are general and not quantifiable and measurable. Time frame is ten years.<br>- The analysed policy document explicitly refers to the international assessment of the obesity problem or to the *WHO Global Strategy on Diet, PA and Health.*
World Health Organization, 2010 [12]	- The following national documents related to PA were identified: *National programme against obesity 2005–2009* (2005); *National Health Plan 2004–2010* (2005); and *National Sports for All Programme Mexa-Se* (2005).<br>- There has been a coordinating mechanism in the area of PA promotion, with the

	National Institute for Sport as the leading institution. The participating stakeholders are government departments on sport.
Daugbjerg et al., 2009 [11]	- Policy document *National Health Plan 2004-2010 Volume I—Priorities* (2004) contains<br>quantifiable PA goals and a requirement/intention for evaluation, but it does not contain a budget for policy implementation.
Branca et al., 2007 [80]	- *National Programme against Obesity 2005–2009* was published in 2005 by the Ministry of Health.
World Health Organization, 2007 [227]	- The *National programme against obesity* and the *National health plan 2004–2010* both include PA.<br>- The key initiative is *Move it* campaign that aims to promote PA and sport.
Puerto Rico	
Ramirez Varela et al., 2016 [32]	- No national/subnational PA plan.
Qatar	
Al-Kuwari et al., 2016 [63] [64]	- The assigned grade for the indicator *National Policy*, *Strategy, and Investment* from *RC on PA for Children and Youth* is B.<br>- Health and PA of youth and children are key concerns.<br>- National PA guidelines, issued in 2014, and a national action plan for PA and nutrition have been developed.<br>- There is inadequate data on evaluation and implementation of health and PA policies.
Ramirez Varela et al., 2016 [32]	- A national or subnational PA plan is available (details are not specified).
World Health Organization, 2014 [232]	- There is a national strategy or policy addressing PA and diet/nutrition (details not specified).<br>- It has been reported that national PA recommendations have been developed.<br>- There is a national coordinating committee for PA.<br>- National policy documents cover: the general population; early years children; children and young people; older adults; workforce/employees; women; people with disabilities; the clinical population with chronic diseases; sedentary/the most inactive people; and families.<br>- The settings covered by national policy documents include: kindergarten; primary schools; high schools; colleges/universities; primary health care; clinical health care; workplace; senior/older adult services; sport and leisure; transport; tourism; environment; and urban design/planning.
Romania	
Hämäläinen et al., 2016 [117]	- The Prime minister and the Ministry of Education and Research are responsible for the document *Movement for health* (2003). It aims to contribute to improving population's health through PE and sport. The document does not include research or other evidence and equality or equity issues. Some of the goals/actions in the document include the following: sport facilities administered by public authorities shall be available for PA recreational purposes at least two hours, three times a week to every citizen; organisation of sports events for the whole population; development of actions and programmes for children and youth.<br>- The Romanian Federation "Sport for All", the Ministry of Health and Family, and the Ministry of Youth and Sport are responsible for the document *Sport for all 3rd Millennium Romania – A Different Lifestyle* (2001). The policy does not mention equality of equity issues. It aims to promote health and "Sport for All", recreation, and education to become a part of people's lifestyles. Some of its subprogrammes include *Old-Sport, Rural Sport, Baby-Sport, Fun-Sport,* and *A chance for everybody.* Some of its goals are to: improve partnerships between economic agents, civil society, and government structures; ensure everyone has free access to PA; and ensure essential conditions like logistics, quality services, management, and human resources for engaging in LTPA in organised settings or independently, in a clean and safe environment.<br>- Besides the general population, some of the target groups mentioned in the documents are:<br>students and teachers; seniors; preschool children; the population in the rural areas; women; and Romanian diaspora.

Hämäläinen et al., 2016 [115]	- HEPA policies cooperation and coordination processes and structures include: government/regional/local committees or working groups with cross-sector representatives; contacts between public sector officers responsible for HEPA between levels; scientific advisory groups/institutes/individuals; formal consultation on HEPA policy for stakeholders; and field visits to make a policy.<br>- There are no: national/regional/local politically elected councils; established systems of policymaking; intersectoral committees or working groups for HEPA; steering committees; administrative working groups including only public sector officers; private sector involvement in policymaking; and public hearings for citizens.
Ramirez Varela et al., 2016 [32]	- PA is mentioned in the document entitled *Joint Programming Initiative A Healthy Diet for a Healthy Life.*
Kahlmeier et al., 2015 [127]	- National PA recommendations are reported to exist, but no other details are available.
World Health Organization, 2010 [229]	- Policy documents in the PA area are not yet available.
World Health Organization, 2010 [12]	- The following national documents related to PA were identified: *Sport Law No. 69/2009 with further amendments and supplements*, issued in 2009, and *Governmental Decision No. 1573/2004 for approval the List of 410 investment objectives of the Programme "Construction of sport facilities"*, issued in 2004.<br>- Since 2005, there has been a coordinating mechanism in the area of PA promotion, and the leading institution has been the National Authority for Youth and Sport. The participating stakeholders are: government departments on youth and sport, research, and education; and the Romanian Olympic and Sports Committee.
Russian Federation	
Ramirez Varela et al., 2016 [32]	- No national/subnational PA plan.
Kahlmeier et al., 2015 [127]	- The national PA recommendations were published in the document entitled *Modern science-based recommendations to optimise the level of physical activity in the population,* issued in 2011.<br>- Recommendations are provided for adults only.<br>- The PA recommendations are fully aligned with the WHO recommendations.<br>- The document includes recommendations on muscle-strengthening activities and SB.
World Health Organization, 2010 [229]	- Policy documents in the PA area are not yet available.
World Health Organization, 2007 [227]	- A PA action plan is under development.
Rwanda	
Ramirez Varela et al., 2016 [32]	- No national/subnational PA plan.
San Marino	
World Health Organization, 2010 [229]	- Policy documents in the PA area are not yet available.
Samoa	
Ramirez Varela et al., 2016 [32]	- PA is mentioned in the document entitled *Health sector plan 2008-2018.*
Lachat et al., 2013 [140]	- In 2008, the Ministry of Health issued the *Health sector plan 2008–2018*.<br>- National policy includes the following targets and actions for PA promotion: health promotion programmes should focus on PA as one of four high-risk areas; focus should be placed on women communities, government workers and community groups to support PA and a healthy lifestyle; and PA should be promoted in homes for elderly people.
Saudi Arabia	
Ramirez Varela et al., 2016 [32]	- PA is mentioned in the document entitled *The Health Promotion Programme and healthy lifestyle (2015).*
World Health Organization, 2014 [232]	- There is a national strategy or policy addressing PA and diet/nutrition (details not specified).<br>- There is a policy that mentions the obligatory inclusion of both girls and boys in PE.<br>- International recommendations and guidelines on PA are used instead of national ones.

	- National policy documents cover: the general population; children and young people; older adults; women; and families.<br>- The settings covered in national policy documents include: primary schools; high schools; colleges/universities; primary health care; senior/older adult services; sport and leisure; and environment.
Scotland	
Coenen et al., 2017 [38]	- In the publication *Start active, stay active - A report on physical activity for health from the four home countries' chief medical officers*, issued by the Government's Department of Health in 2011, it is stated that "all adults should minimise the amount of time spent being sedentary (sitting) for extended periods".
Ramirez Varela et al., 2016 [32]	- The PA plan entitled *A More Active Scotland (2014)* is available.
Reilly et al., 2016 [180] [181]	- The assigned grade for the indicator *National Policies, Strategies and Investment* from *RC on PA for Children and Youth* is B.<br>- Scotland's PA policy "has sought to take advantage of the hosting of the 2014 *Commonwealth Games* to provide a population-wide PA 'legacy'".<br>- There is still limited evidence on policy implementation, but the situation is better than in 2013, with "outcome agreements between national and local government in relation to policy".<br>- There are multiple relevant policies for PA promotion, but there is a lack of policies on SB.
Kahlmeier et al., 2015 [127]	- The national PA recommendations were published in the document entitled *Physical Activity, Health Improvement and Protection. Start Active. Stay Active: A report on physical activity from the four home countries',* issued in 2011.<br>- Specific recommendations are provided for children/young people, adults, and older adults.<br>- The PA recommendations for children/young people and adults are fully aligned with the WHO recommendations.<br>- The document includes recommendations on muscle-strengthening activities for adults and older adults and recommendations on SB for children/young people, adults, and older adults.
Vallgårda, 2015 [209]	- In 2010, Government published *Preventing overweight and obesity in Scotland. A Route Map Towards Healthy Weight.* In the plan, stronger responsibility is put on the government (to act by changing the environment) than on individuals (to change their behaviours).
Christiansen et al., 2014 [44]<br>World Health Organization, 2011 [231]	- The following policies related to HEPA and sport promotion were identified: *Reaching higher – building on the success of sport 21*, issued in 2007 and the action plan *Our plan: corporate plan 2009–2011, Sportscotland*, issued in 2009. In this study, the two policy documents were referred to as a single strategy<br>- The strategy mentions the *Active Schools* programme as a way of reaching "hard-to-reach groups" such as children with disabilities, girls, and inactive children.
Reilly et al., 2014 [179]<br>Reilly et al., 2013 [178]	- The assigned grade for the indicator *National Policies, Strategies and Investment* from *RC on PA for Children and Youth* is B.<br>- There are several limitations mentioned related to this indicator, including: no policies on SB; most policies have not been evaluated; policy implementation may be limited; and there is no policy designed to address rural/urban or socio-economic disparities.
Halliday et al., 2013 [114]	- Strategy *Let's Make Scotland More Active* is one of few policy documents that was in depth, systematically reviewed in 2008 - *Physical activity policy review*, by the NHS Health Scotland. It was found that more supportive PA policies for elderly should be addressed, And that the strategy should put more focus on inclusion in general, and that there is a gap between action and intent at various levels of government, that is, insufficient resources and political commitment towards PA promotion. The strategy stimulated local development of strategies across the country.
Woods & Mutrie, 2012 [218]	- In 2003, all political parties agreed on *Let's Make Scotland More Active policy.* It was reviewed in 2009 and one of the conclusions was that policy is still relevant and that the targets set for 2022 could be met with refined efforts. For example, adolescent girls and older adults are making slower progress towards achieving the targets. More attention should be given to such population groups.
Brown et al., 2011 [43]	- National policy document *Lets make Scotland more active* was published by the Physical Activity Task Force in 2003.

Pate et al., 2011 [37]	- Policy related to PE in schools states that: children should engage in at least two hours of PE a week; PE should be taught by highly qualified and certified teachers; and PE curriculum should be included in the school review process.<br>- PA policy in the area related to community environmental support states that: access to public sports facilities and venues should be free or offered at discounted prices for students; parks, playgrounds, and open spaces that are interesting and challenging for youth and children should be created; community organisations and local authorities should be support, funded, and encouraged to develop PA promotion programmes for families to help them become active and use the existing infrastructure; and cooperation between colleges, youth clubs, and schools with community groups, local authorities, and health professionals should be encouraged to design programmes to increase involvement in PA..<br>- The data were extracted from the following documents: *Let's Make Scotland More Active: A Strategy for Physical Activity* (2003) and *Five-year Review of 'Let's Make Scotland More Active'—A Strategy for Physical Activity* (by the Scottish Physical Activity Research Collaboration, 2009).
World Health Organization, 2010 [12]	- The following national documents related to PA were identified: *Let's make Scotland more Active: A Strategy for Physical Activity* (2003), *Improving Health in Scotland* (2003), and *Transport Scotland, framework document* (2005).
Bornstein et al., 2009 [79]	- The document *Let's Make Scotland More Active—a Strategy for Physical Activity* (2003) has the time frame for achieving goals from 2003 until 2022. It aims to increase the population level of PA by one per cent a year. Some of the targeted population groups are: young people and children; adults later in life; and adults.
Musingarimi, 2009 [158]<br>Musingarimi, 2008 [157]	- *Let's Make Scotland More Active—a Strategy for Physical Activity* Some aims to, achieve that by 2022 50% of people (aged 16 years and older) and 80% of children (younger than 16 years) meet recommended PA levels. The progress towards targets is measured with the Scottish Health Survey.
Bull et al., 2004 [26]<br>Schöppe et al., 2004 [187]<br>Bull et al. in Bull et al., 2004 [86]	- The Scottish Executive initiates national PA policies and provides funding. In 2003, it launched *Sport 21 2003-2007 shaping Scotland's future* for which Sportscotland is the main implementation agency. The agency promotes the impact of sport on a broader policy agenda, especially in areas of health and social inclusion.<br>- Following a commitment in Government's *White Paper Towards A Healthier Scotland* (1998), in 2001, Scottish Ministers established the National Physical Activity Task Force. - Other PA promotion related documents include: *A Walking Strategy for Scotland* (2003); *Building better Transport* (2003); *Coronary Heart Disease and Stroke Strategy for Scotland* (2002); and *The National Cycling Strategy in Scotland* (1996).
Senegal	
Ramirez Varela et al., 2016 [32]	- No national/subnational PA plan.
Serbia	
Kahlmeier et al., 2015 [127]	- National PA recommendations have not yet been developed.
Lachat et al., 2013 [140]	- In 2010, the Ministry of Health issued the *National programme for prevention, treatment*<br>*and control of cardiovascular diseases in Republic of Serbia till 2020*.<br>- National policy included the following targets and actions for PA promotion: promote PA in adults, elderly, healthy people, patients with cardiovascular disease, children, and adolescents; the NGO and Government campaign "Sport for All"; "institutional organisation of sport and recreative occasions" (e.g. sport activities or recreation for pensioners or workers); implement and promote PA in everyday life at the population level; perform moderate PA as stressed out in the national guidelines; update PA programmes in the school curriculum; educate PA and medical professionals about benefits of PA for patients with cardiovascular disease; and develop and implement cooperation between NGOs and government in the implementation of PA recommendations.<br>- The policy mentioned concrete actions for involvement of private sector in PA promotion.
Seychelles	
Ramirez Varela et al., 2016 [32]	- The PA plan entitled *Sports Strategic Plan for 2014 -2018* is available.

Singapore	
Ramirez Varela et al., 2016 [32]	- The PA plan entitled *Physical activity strategy paper* is available.
Pate et al., 2011 [37]	- PA policy in the area related to school environmental support for PA promotion in children and youth states that: access to a broad range of activities such as dance, sports, active travel, play, exercise and being active in daily tasks should be provided around and in school; schools should be encouraged to conduct fitness tests of their students annually and store the collected data; parks around schools and school playgrounds should be designed and renovated to inspire sport, movement, play, and outdoor education; awards should be given to schools for promoting holistic health in the school settings, including the promotion of PA in students, parents, and staff.<br>- PA policy in the area related to community environmental support for PA promotion in children and youth states that: access to public sports facilities and venues should be free or offered at discounted prices for students; parks, playgrounds, and open spaces that are interesting and challenging for youth and children should be created; community organisations and local authorities should be support, funded, and encouraged to develop PA promotion programmes for families to help them become active and use the existing infrastructure; and cooperation between colleges, youth clubs, and schools with community groups, local authorities, and health professionals should be encouraged to design programmes to increase involvement in PA.<br>- The data were obtained from the Health Promotion Board Singapore Government (information provided by co-author, further details not specified).
Slovak Republic	
Ramirez Varela et al., 2016 [32]	- PA is mentioned in the document entitled *National Health Promotion Programme.*
Christiansen et al., 2014 [44]<br>World Health Organization, 2011 [231]	- The following policy related to HEPA and sport promotion was identified: *National programme for the development of sport 2001–2010* (2001). It identified PE as one of the seven key focus areas. It mentioned variety of actions such as: the inclusion of disadvantaged children; enabling access to facilities; providing opportunity for children to have three PE lessons per week; ensuring training of teachers; and ensuring quality standards.
World Health Organization, 2010 [12]	- The following national documents, related to PA were identified: *Č.300/2008 Law on the organisation and promotion of sport* (2008); *National programme for sport development* (2001), and *National Health Enhancing Physical Activity Programme* 2007–2012. The first two documents were issued by the Government and the last one was issued by the Ministry of Health.<br>- Since 2001, there has been a coordinating mechanism in the area of PA promotion, with the Ministry of Education as the leading institution. The participating stakeholders are: government departments on sport, health, transport, labour, education, research, and food; communities; and academia.
World Health Organization, 2007 [227]	- There are two main documents dealing with overweight and obesity: *Health state policy* (2006) and the *National health promotion programme*.<br>- The *National programme for sport development* was approved in 2001 by the Government. Some of the main priorities are sports as a leisure activity and PA in schools. A legislative document related to sports is under development.
Slovenia	
Ramirez Varela et al., 2016 [32]	- PA is mentioned in the document entitled *Joint Programming Initiative A Healthy Diet for a Healthy Life.*
Sember et al., 2016 [192]	- The assigned grade for the indicator *Government—Strategies, Policies, Investments* from *RC on PA for Children and Youth* is B +.<br>- The Slovenian Parliament adopted the *National Programme of Sport 2014–2023* in 2014 and the *National Programme of Nutrition and Physical Activity for Health 2015–2025* in 2015.<br>- The strategies aim to: provide grounds for the implementation of high-quality, publicly funded, and organised PA programmes at all levels; encourage general population to engage in healthy lifestyles; and raise the quantity and the quality of children and adolescents' PA. The actions proposed in the *National Programme of Sport* include: free cycling and swimming lessons; providing leisure-time for sport activities; and ensuring at least 180 minutes PE per week.

	- Constant investments in sport infrastructure at all governmental levels are visible in the last two decades. Public funding for sport infrastructure exceeded €300 M in the period from 2001 to 2008.<br>- The *Ministry of Education, Science, and Sports* supports the SLOfit monitoring system and uses the evidence to plan its future interventions and activities.<br>- The government's PA intervention programme *Healthy Lifestyle* aimed to combat obesity and decline in children's physical fitness was evaluated and proved to be effective.
Kahlmeier et al., 2015 [127]	- The national PA recommendations were published in the document entitled *National Health Enhancing Physical Activity Programme 2007–2012,* issued in 2007.<br>- Recommendations are provided for adults only.<br>- The PA recommendations are not fully aligned with the WHO recommendations.<br>- The document includes recommendations on muscle-strengthening activities.<br>- The document does not include recommendations on SB.
Bull et al., 2014 [85] [83] [84]	- PE is a mandatory subject from kindergarten to university.<br>- The key document for PA promotion is the *National Health Enhancing PA Programme 2007-2012.*<br>- Other relevant documents related to PA promotion are*: Law of Sport of the Republic of Slovenia* (1998); *National Programme of Sport in the Republic of Slovenia, 2000-2010, Occupational Health and Safety Act* (1999); *Resolution on National Programme of Safety and Health at Work* (2003); Spatial Planning Act (2002, 2007); *Spatial Development Strategy of the Republic of Slovenia* (2004); and *Resolution on the Transport Policy of the Republic of Slovenia 2006* (particularly important for cycling and walking).<br>- There are national PA guidelines for adults only.
Christiansen et al., 2014 [44]<br>World Health Organization, 2011 [231]	- The document *National programme on sports* [Nacionalni program športa v Republiki Sloveniji] was published in 2000. It mentions short-term, medium-term, and long-term time frames. It aims at increasing the number of people who regularly engage in sport activities by 2.5 per cent with one per cent increase a year.<br>- Standardisation of sports facilities and the need to provide young people access to use them on a "non-profit basis" have been addressed. Reconstruction or construction of 25,000m^2 of covered space per year is also one of the aims mentioned in the document.
Ceccarelli et al., 2011 [90]	- The analysed policy document (details not specified) explicitly refers to the international assessment of the obesity problem or to the *WHO Global Strategy on Diet, PA and Health.*<br>- The document includes a thorough analysis of PA and eating habits of the population.<br>- The specific quantifiable targets mentioned in the document are:<br>a) for adolescents and children: increase the number of those who engage in PA at least one hour a day by 30% and who cycle and/or walk for transport purposes by 20%, and decrease the number of those with more than four hours of leisure screen time a day by 30%.<br>b) for adults: increase the number of those who are regularly and sufficiently engaged in PA by 20% and who cycle and/or walk for transport purposes by 20%, and decrease the number of those with more than four hours of leisure screen time a day by 30%.
World Health Organization, 2010 [12]	- The *National Health Enhancing Physical Activity Programme 2007–2012* was issued in 2007 by the Ministry of Health.<br>- Since 2007, there has been a coordinating mechanism in the area of PA promotion, with the Ministry of Health and the Ministry of Education and Sport as the leading institutions. The participating stakeholders are government departments on sport, health, research, and education.
World Health Organization, 2007 [227]	- The Ministry of Health prepared a draft of the *National Plan for Physical Activity*. In 2006, it was sent to the Government.<br>- Some of the National Institute of Public Health's initiatives are: *The Healthy Nutrition and Physical Activity for Secondary School Teachers Programme (2004–2005*); *Body Weight for Adolescents and Getting Active (2004-2006)*; and *That's me* – a web site with information for adolescents that includes topics on PA and nutrition.
Solomon Islands	
Ramirez Varela et al., 2016 [32]	- NCD plan includes PA (details are not specified).

Lachat et al., 2013 [140]	- National policy (details not specified) includes the following targets and actions for PA promotion: help individuals with disabilities caused by a traumatic injury, disease or other factors to maximise their potential for engaging in PA; and promote maintenance of body weight by combining regular PA and balanced diet.
South Africa	
Ramirez Varela et al., 2016 [32]	- PA is mentioned in the documents entitled *National Plan for the Prevention and Control of Non-Communicable Diseases 2013-2017* and *National Strategy for the Prevention and Control of Obesity 2015-2020.*
Uys et al., 2016 [208]	- The assigned grade for the indicator *Government—Strategies, Policies*, *Investments* from *RC on PA and nutrition for Children and Youth* is B.<br>- Sports and Recreation South Africa (SRSA) published the *Sports and Recreation South Africa Annual Performance Plan (APP) 2016/17* and mentioned the importance of joint commitment with the Department of Basic Education (DBE) to maximise access to PA, recreation, and sport in all schools.<br>- SRSA and other stakeholders have been actively engaged in ensuring funding and supporting private partnerships to increase the participation in school sport.
Draper et al., 2014 [100] [101]	- The assigned grade for the indicator *Government—Strategies, Policies*, *Investments* from *RC on PA and nutrition for Children and Youth* is B.<br>- *Integrated School Sports Framework* (2011) was launched by the DBE and SRSA and it guides the delivery of sport in all schools by hosting and funding tournaments, providing equipment, and building capacity of trainers.<br>- The *National Sport and Recreation Plan* increased the number of active students at school.<br>- The *Sport and Recreation South Africa strategic plan for the fiscal years 2012 – 2016* mentioned the increase of the school sport investment by SRSA of 18%.
de Villiers et al., 2010 [97]	- The following grades were assigned to the four components of the indicator *Policies, programmes and interventions to promote PA* from *RC on PA, Nutrition and Tobacco use for Children and Youth*:<br>1) Department of Education's Curriculum and Assessment Policy Statement – the grade "NE" (i.e. "promising initiatives but for which there is no evaluation").<br>- *Curriculum and Assessment Policy Statements* (2011) mentions fixed periods dedicated to PE in grades 10-12, each week;<br>2) School based interventions – grade "NE";<br>3) Non-motorised transport initiative – grade "NE";<br>4) Sport for development initiatives – grade "B".
Reddy et al., 2007 [177]	- The assigned grades for the indicator related to PA promotion *Legislation: Sport and Education* from *RC on PA, Nutrition and Tobacco use for Children and Youth* are B and NE.<br>- A framework document for cooperation was developed in 2005 by the Departments of Education and Sport and Recreation (details not specified). It mentioned several factors that are preventing the transformation of recreation and sport, including: the lack of safe and appropriate facilities and improvement of existing ones; lack of participation in PE by learners and educators; and the fact that population groups such as girls and women, rural communities, and people with disabilities are disadvantaged.
Spain	
Ramirez Varela et al., 2016 [32]	- The PA plan entitled *Plan Integral de Actividad Fisica y Deporte (Integral Plan for Physical Activity and Sport Promotion)* is available.
Roman-Viñas et al., 2016 [184]	- The iandicator *Government* from *RC on PA for Children and Youth* did not receive a grade. It was marked as incomplete, because the impact of policies on increased PA levels was unclear.<br>- The *Integral plan for Physical Activity and Sport 2010-20* promotes universal access to sport at the population level. It mentions specific policies for PA promotion at the worksite, in school, and in the private and health sectors.
Kahlmeier et al., 2015 [127]	- National PA recommendations have not yet been developed.
Rütten et al., 2013 [185]	- The *Spanish Strategy for Nutrition, PA, and prevention of obesity National Strategy on sport and sustainability* issued in 2007 includes public policy related to LTPA. More policies related to LTPA promotion are available at local/regional levels.<br>- Supportive environments for LTPA (indoor/outdoor sport facilities and infrastructure for LTPA) are broadly available. Urban and green spaces usable for LTPA are partially

	available.
Ceccarelli et al., 2011 [90]	- The identified goals in policies that deal with nutrition, PA, and obesity are general and not quantifiable and measurable (details not specified).<br>- The analysed policy document explicitly refers to the international assessment of the obesity problem or to the *WHO Global Strategy on Diet, PA and Health.*
World Health Organization, 2010 [12]	- The following national documents related to PA were identified: *Integral Plan for Physical Activity and Sport Promotion* (2009); and *Strategy for Nutrition,* and *Physical Activity and Prevention of Obesity* (2005).<br>- Since 2001, there has been a coordinating mechanism in the area of PA promotion, with the Spanish Food Safety and Nutrition Agency, Ministry of Health as the leading institution. The participating stakeholders are: government departments on agriculture, food, health, consumer affairs, education and research, social welfare, and sport; civil society; communities (regional councils); academia; media; and the private sector.
Daugbjerg et al., 2009 [11]	- The *Spanish strategy for Nutrition, Physical Activity and Prevention of Obesity* (2002) does not contain quantifiable PA goals, the time frame, and the budget for policy implementation, but it contains a requirement/intention for evaluation.
Ballesteros Arribas et al., 2007 [70] (in Spanish language)	- *Spanish strategy for nutrition, physical activity and the prevention of obesity* (NAOS strategy) was issued by the Ministry of Health and Consumer Affairs in 2005. The least developed part of the strategy is related to PA. It is more focused on nutrition. It contains the pyramid that for the first time provided combined recommendations on diet and PA, because the authors of the Strategy considered the recommendations on these health behaviours should be delivered as “one message”.
World Health Organization, 2007 [227]	- The *Spanish Strategy for nutrition, physical activity and the prevention of obesity* addressed obesity through various working groups that focus on, for example: targets for PA and diet; genetic, environmental and educational determinants of obesity; and scientific research. Some of its key objectives include the promotion of PA and healthy eating and increasing the awareness about how regular PA and balanced diet may affect health. Various stakeholders participated in the consultation process for its development. During the process, the Ministry of Health and Consumer Affairs signed various cooperation agreements with the private sector.
Sri Lanka	
Ramirez Varela et al., 2016 [32]	- A national or subnational PA plan is available (details are not specified).
Lachat et al., 2013 [140]	- In 2004, Sri Lanka Inter Ministerial Committee on Food Security issued the *Food and nutrition policy of Sri Lanka, 2004–2010*.<br>- National policy includes the following targets and actions for PA promotion: reactivate the young farmers club and sports and youth clubs to promote PA; raise awareness of PA; promote higher amount of PA among school children and adults to minimise the risk of chronic degenerative diseases; develop awareness programme on the importance of PA for the employees in institutions and promote the messages about the importance of PA through the mass media; supply facilities for outdoor recreation PA; and invest in road safety for cyclists and pedestrians.
St. Kitts and Nevis	
Ramirez Varela et al., 2016 [32]	- PA is mentioned in the document entitled *Strategic Plan of Action for the Prevention and Control of Non-communicable diseases for countries of the Caribbean Community 2011-2015.*
St. Lucia	
Ramirez Varela et al., 2016 [32]	- PA is mentioned in the document entitled *Strategic Plan of Action for the Prevention and Control of Non-communicable diseases for countries of the Caribbean Community 2011-2015.*
St. Martin (French part)	
Ramirez Varela et al., 2016 [32]	- No national/subnational PA plan.
St. Vincent and the Grenadines	
Ramirez Varela et al., 2016 [32]	- PA is mentioned in the document entitled *Strategic Plan of Action for the Prevention and Control of Non-communicable diseases for countries of the Caribbean Community 2011-2015.*
Swaziland	

Ramirez Varela et al., 2016 [32]	- No national/subnational PA plan.
Sweden	
Coenen et al., 2017 [38]	- The document *Swedish Guidelines on Call Centre Workplaces*, issued by the Government's Work Environment Authority in 2007, includes the recommendation to alternate between standing and sitting to reduce occupational SB.
Nyström et al., 2016 [161] [162]	- The assigned grade for the indicator *Government Strategies and Investments* from *RC on PA for Children and Youth* is B.<br>- *Nordic Nutrient Recommendations* provide guidelines for children and adults on diet, nutrition, and PA levels. Specific recommendations on SB are not provided. It is only recommended to reduce the amount of time spent in SB.<br>- In 2009, the Swedish Sports Confederation issued *Sports for Life – Strategic Plan for the Sport Movement's Public Health Work* and *Sports Wants – Policy Programme of Ideas*.
Ramirez Varela et al., 2016 [32]	- No national/subnational PA plan.
Kahlmeier et al., 2015 [127]	- The national PA recommendations were published in the document entitled *Physical activity. In: Nordic Nutrition Recommendations 2004 Integrating nutrition and physical activity,* issued in 2004 by the Nordic Council of Ministers.<br>- Specific recommendations are provided for children/young people and adults.<br>- The PA recommendations are not fully aligned with the WHO recommendations.<br>- The document includes recommendations on muscle-strengthening activities for children/young people.<br>- The document does not include recommendations on SB.
Kalman & Hamrik, 2013 [128]<br>(in Czech language)	- In a policy document (details not specified) related to PA it is mentioned that special emphasis needs to be put on environmental conditions for children and youth, considering their socioeconomic status, gender, and ethnic background. It is stated that the interest for LTPA has increased in the last decade, but low levels of PA related to workplace and transport have not been addressed properly.
Ceccarelli et al., 2011 [90]	- The analysed policy document (details not specified) explicitly refers to the WHO Global Strategy on Diet, Physical activity and Health or to some other document assessing the problem of obesity.<br>- Sports goods manufacturers and advertising and recreation businesses were mentioned in the policy document as the stakeholders related to PA and sport.:.<br>- *Health, food and physical activity: Nordic Plan of Action on better health and quality of life through diet and physical activity* was issued by the Nordic Council of Ministers in 2006. The document established a number of common goals and the policy agenda for all Nordic countries, although individual countries have adopted "partly different sets of actions within the designated areas of common priority".
Pate et al., 2011 [37]	- PA policy measures were found in the following areas: a) PE in school (e.g. requirement of at least two hours of weekly PE for all children that should be taught by highly qualified and certified teachers); b) health education (e.g. enable training for healthcare professionals in schools in motivational interviewing techniques related to PA, establishment of cooperation with universities to provide teacher education classes for inclusion of the *PA and Health* topic into the curriculum); c) school environmental support (e.g. provide access to a broad range of activities such as dance, sports, active travel, play, exercise, and being active in daily tasks around and in schools, award schools for promoting holistic health in the school setting, including the promotion of PA in students, parents, and staff.<br>- The data were extracted from the *Background Material to the Action Plan for Healthy Dietary Habits and Increased Physical Activity* published by the Swedish National Food Administration and National Institute of Public Health in 2005.
Bornstein et al., 2009 [79]	- In the *Background Material to The Action Plan for Healthy Dietary Habits and Increased Physical Activity* timeline for achieving goals was not identified. The target groups include, for example: elderly; children; and people with immigrant backgrounds. Some of the targets are: increase the number of healthy adults who engage in at least 30 minutes of moderate PA every day or in total 3.5 hours of moderate PA per week; and increase the number of healthy children who engage in moderate PA for at least 60 minutes every day or in total for seven hours per week.
Branca et al., 2007 [80]	- *Background material to the action plan for healthy dietary habits and increased*

	physical activity was published in 2005 by the National Institute of Public Health. It is a comprehensive document, containing 79 detailed measures with the description of responsible stakeholders, rationale for the measures, and in some cases the cost estimates. It proposes to establish a consortium with representatives from different research councils to initiate intervention research on diet and PA. It also focuses on housing policy, because it can have major effects on PA and public health.
World Health Organization, 2010 [12]	- The following national documents related to PA were identified: *Government Bill: future outdoor recreation, 2009/10:238* (2010); *A renewed public health policy* (2008); *Future travel and transport – infrastructure for sustainable growth* (2008); *Nordic Plan of Action on better health and quality of life through diet and physical activity* (2006); *Action Plan for healthy dietary habits and increased physical activity* (2005); *Sweden's environmental policy: Environmental Quality Objective 15 – A good built environment* (2005); *Sweden's new public health policy* (2002); *The will of sports* (issued in 1995, updated in 2005 and 2009); and *Physical education and health* (1995).<br>- Since 2001, there has been a coordinating mechanism in the area of PA promotion, with the Swedish National Public Health Institute and the Swedish National Centre for Child Health Promotion as the leading institutions. The participating stakeholders are: government departments on sport and health; communities; NGOs; and the private sector.
Daugbjerg et al., 2009 [11]	- The policy document entitled *Sweden's new public health policy— National public health objectives for Sweden* (2003) does not contain quantifiable PA goals, the time frame, the budget for policy implementation, and a requirement/intention for evaluation. From all the abovementioned components, the document *Healthy dietary habits and increased physical activity—the basis for an action plan* (2005) contains only a requirement/intention for evaluation.
World Health Organization, 2007 [227]	- *Sweden's new public health policy: national public health objectives for Sweden*, published in 2003, deals with both nutrition and PA and mentions increased PA as one of its 11 target areas. The policy highlights the significance of 'good sports policies that enhance opportunities of people to exercise and engage in sports activity. PA in preschools and schools is seen as crucial. It is stated that the area of health and sports should develop new working methods that encourage and allow all children to participate in PA and that PA during working hours is also of great importance.
Van Mechelen in Simonopoulos (ed.), 1997 [210]	- There are no national policies for the promotion of PA or physical fitness. A national programme is in development, whilst some local initiatives for promotion of active lifestyle are already in place.
Switzerland	
Ramirez Varela et al., 2016 [32]	- The PA plan entitled *National Programme on Diet and Physical Activity* is available.
Kahlmeier et al., 2015 [127]	- The national PA recommendations were published in the document entitled the *Physical activity and health-what are the recommendations and where do we find the Swiss population?,* issued in 2009.<br>- Specific recommendations are provided for children/young people and adults.<br>- The PA recommendations are not fully aligned with the WHO recommendations.<br>- The document includes recommendations on muscle-strengthening activities for children/young people and adults.<br>- The document includes recommendations on SB for children/young people.
Bull et al., 2014 [85] [83] [84]	- *The Freedom to Roam* (1907) and *Federal Law on Walking and Hiking Paths* (1985) are important parts of historic legislation related to transport and environment. The *Mission statement on human powered mobility* (only a draft document) aims to increase physically active transport in the next ten years by 15%.<br>- *The Federal Law on the Promotion of Gymnastics and Sport* (1972) besides sports covers also the provision of PE.<br>- *Concept of the Federal Council for a Sports Policy in Switzerland* (2000) set the target of increasing the number people who engage in PA by 1% per year from 2003 to 2006.<br>- The *National Programme on Diet and Physical Activity* 2008-2012 does not include an action plan.<br>- National HEPA recommendations for adults and children were issued in 1999 and in 2006, respectively.
Bellew et al., 2011 [8]	- The *Youth+Kids* programme targets elementary school children.<br>- The *Switzerland Mobility* programme provides national tourism offers for cycling,

	hiking, leisure-orientated walking, and other activities.
Ceccarelli et al., 2011 [90]	- The analysed policy document (details not specified) explicitly refers to the *WHO Global Strategy on Diet, Physical activity and Health* or to some other document assessing the problem of obesity.<br>- The document includes a thorough analysis of PA and eating habits of the population.
World Health Organization, 2010 [229]	- *The Swiss Nutrition and Physical Activity Monitoring Programme* was set up to be in effect from 2007 to 2013. Under the programme, the Federal Office of Public Health launched an *Action santé* initiative. The three key principles of the initiative were subsidiarity, openness, and action. The key goal of the initiative was to improve people's quality of life by enabling them to live in an environment that is health-promoting and suitable for the implementation of healthy lifestyles, including a healthy diet and sufficient PA. The initiative has four key action areas, including: advertising and marketing; food composition; consumer information; and "the promotion of an environment conducive to physical activity".<br>- Intersectoral cooperation is enabled through involvement of non-health departments, for example, the Sport and Transport Department.
Daugbjerg et al., 2009 [11]	- The document *Mission statement on human powered mobility—English summary* (published 2002, with the time frame until 2004) contains quantifiable PA goals, the budget for policy implementation, and a requirement/intention for evaluation. *Concept of the Federal Council for a sports policy in Switzerland* (2000) does not contain quantifiable PA goals, time frame and budget for policy implementation, but it does contain a requirement/intention for evaluation.
World Health Organization, 2007 [227]	- *Concept for a national sports policy* was issued by the Federal Council in 2000. It mentions the foundations for the political contribution in creation of PA culture, where sport is an integral part of an economic, ecological, social, and sustainable development.<br>- The Health Promotion Switzerland and the Swiss Federal Office of Public Health launched the joint *Suisse Balance* programme. Its two key goals are to: increase the proportion of people with healthy weight through healthy diet and PA; and develop the structural conditions necessary for the stable development of population's healthy body weight by 2010. It mainly targets children and youth and it encourages the development of national, regional, and local, projects that promote healthy behaviour through PA and nutrition.
Cavill et al, 2006 [89]	- The Swiss Confederation was responsible for sports promotion, including "sport promotion to improve public health", since 1972.<br>- The *Concept for a National Sports Policy in Switzerland* was accepted by the Federal Government in 2000 and its first main objective was "more physically active people".<br>- *Youth+Sports* is a national programme for 10-20 year olds, which has been in place for over 30 years and receives more than 40% of the Federal Office of Sports' budget.
Bull et al., 2004 [26]<br>Schöppe et al., 2004 [187]<br>Bull et al. in Bull et al., 2004 [86]	- The following agencies are responsible for national PA policies: the Swiss Federal Office of Public Health; the Swiss Federal Department of Defence; Civil Protection and Sport; and the Health Promotion Unit (under the Swiss Federal Office of Sports). The Health Promotion Unit is perceived as a national centre for HEPA. Guidelines for evaluating and developing activities in the HEPA promotion were published and summarised in the *PA pyramid*.<br>- The key documents that define PA as a priority area are a strategy document *HEPA Promotion in Switzerland* and the *Swiss Federal Government's Concept for a National Sport Policy.* Both documents were published in 2000. Other documents that address PA promotion are*: Policy and Strategy Document for the Promotion of Cycling and Walking with a particular Focus on Children: Transport related Health, Impacts – Costs and benefits with a particular focus on children* (2003) and the *Action Plan Environment and Health* (2001).
Syrian Arab Republic	
Ramirez Varela et al., 2016 [32]	- A national or subnational PA plan is available (details are not specified).
Tajikistan	
World Health Organization, 2010 [229]	- Policy documents in the PA area are not yet available.

World Health Organization, 2007 [227]	- The programme on PA and diet was recently developed (details not specified).<br>- Policy on obesity prevention is to be developed.
Tanzania	
Ramirez Varela et al., 2016 [32]	- No national/subnational PA plan.
Thailand	
Amornsriwatanakul et al., 2016 [67] [68]	- The assigned grade for the indicator *Government Strategies, Policies, and Investments* from *RC on PA for Children and Youth* is C.<br>- The documents related to PA are the *Thailand Healthy Lifestyle Strategic Plan 2011-2020*, issued by the Ministry of Public Health, and the *National Physical Activity Plan*, issued by the Ministry of Public Health, the Division of Physical Activity and Health.<br>- The Ministry of Tourism and Sport allocated resources for the implementation of recreational and sport programmes and for building infrastructure to promote children's health.<br>- The Government's investments and policies related to PA do not focus on children and existing plans usually do not highlight PA but mainly rather sports and exercise.<br>- It is recognised that PA policy and physical environments may be some of the reasons why levels of children's PA are low.
Ramirez Varela et al., 2016 [32]	- A national or subnational PA plan is available (details are not specified).
Pate et al., 2011 [37]	- Policy related to PE in schools states that: access to a broad range of activities such as dance, sports, active travel, play, exercise, and being active in daily tasks should be provided around and in schools; schools should be encouraged to conduct fitness tests of their students annually and store the collected data; parks around schools and school playgrounds should be designed and renovated to inspire sport, movement, play, and outdoor education; awards should be given to schools for promoting holistic health in the school settings, including the promotion of PA in students, parents, and staff.<br>- The data were obtained from the Thai Health Promotion Foundation (information provided by co-author, further details not specified).
Tonga	
Ramirez Varela et al., 2016 [32]	- NCD plan includes PA (details are not specified).
Trinidad and Tobago	
Ramirez Varela et al., 2016 [32]	- A national or subnational PA plan is available (details are not specified).
Tunisia	
Ramirez Varela et al., 2016 [32]	- No national/subnational PA plan.
Turkey	
Ramirez Varela et al., 2016 [32]	- PA is mentioned in the document entitled *Turkey Healthy Nutrition and Active Life Programme (2014 – 2017).*
Kahlmeier et al., 2015 [127]	- The national PA recommendations were published in the document entitled the *Obesity prevention and control programme of Turkey (2010–2014),* issued in 2010.<br>- Recommendations are provided for adults only.<br>- The PA recommendations are not fully aligned with the WHO recommendations.<br>- The document does not include recommendations on muscle-strengthening activities.<br>- The document does not include recommendations on SB.
Lachat et al., 2013 [140]	- In 2010, the General Directorate of Primary Health Care, Ministry of Health issued the *Obesity prevention and control programme of Turkey 2010–2014*.<br>- National policy includes the following targets and actions for PA promotion: establish mainstream obesity fighting strategies in the national health strategies and policies; provide accurate information to the public by written and visual media on obesity and active life; establish provincial coordination centres in 81 provinces (Obesity Prevention, Nutrition, and Active Life); increase the level of knowledge about PA at worksites; develop national PA guidelines; improve programme related to PA and PA-related environment in the educational system; build recreational areas and sports facilities to make PA widespread, with the leadership of the local administration; and develop "PA applications that can be easily applied inside the house".

	- The policy includes concrete actions for the involvement of private sector in PA promotion and it highlights the need for urban planning and sports infrastructure.
World Health Organization, 2007 [227]	- *The National Plan of Action for Food and Nutrition* covers the period from 2002 to 2010.<br>- The National Food and Nutrition Committee was established to implement and coordinate the realisation of the National plan. The Committee consists of the representatives from the Ministry of Agriculture, the Ministry of Education, and the Ministry of Health, as well as from nongovernmental organisations, universities, private sectors, and production sectors. The Committee is composed of different working groups. One of the working groups deals –is entitled *Prevention of obesity and chronic diseases related to obesity and encouraging an active lifestyle*. The Committee started its work in the fields of obesity and PA and will develop recommendations on advertising aimed at children.
Tuvalu	
Ramirez Varela et al., 2016 [32]	- NCD plan includes PA (details are not specified).
Turkmenistan	
World Health Organization, 2010 [229]	- Policy documents in the PA area are not yet available.
Uganda	
Ramirez Varela et al., 2016 [32]	- No national/subnational PA plan.
Ukraine	
Ramirez Varela et al., 2016 [32]	- The PA plan entitled *Physical Activity - Health Lifestyle - Healthy Nation 2025* is available.
Kahlmeier et al., 2015 [127]	- National PA recommendations have not yet been developed.
United Arab Emirates	
Ramirez Varela et al., 2016 [32]	- No national/subnational PA plan.
Zaabi et al., 2016 [223] [224]	- The assigned grade for the indicator *Government Strategies and Investments* from *RC on PA for Children and Youth* is B+.<br>- Government demonstrated commitment and leadership in providing PA opportunities for all youth and children and invested significant funds in PA promotion programmes for children and adults (e.g. redesigning the transport infrastructure and improving the urban environment).<br>- Since 2010, PE classes are obligatory in all schools, from year one (kindergarten) to year 12 (high school).<br>- As one of the goals, the document *UAE Vision 2021* stressed out achieving the status of a world-class healthcare system with major performance indicators related to PA (e.g., reduction of the prevalence of obesity among 5-17 year olds by 12% by 2012).
World Health Organization, 2014 [232]	- A national policy document related to PA in the health sector was not identified.<br>- Other findings from this study related to PA policy in United Arab Emirates are related to local level (*Dubai Sports Strategy*, *Dubai Pulse*).
United States of America	
Coenen et al., 2017 [38]	-The *Physical Activity Guidelines for Americans*, issued by the Department of Health and Human Services in 2008, do not include recommendations on SB.
Ramirez Varela et al., 2016 [32]	- The PA plan entitled *US National Physical Activity Plan* is available.
Guo&Pan, 2016 [113] (in Chinese language)	- The recent history of PA policy in the USA had five turning points.<br>- The first three turning points relate to PA recommendations issued by professional organisations (by the American College of Sports Medicine in 1990; by the American Heart Association in 1992; and by the American College of Sports Medicine and the Centres for Disease Control and Prevention in 1995).<br>- The fourth turning point relates to the development of the *Physical Activity Guidelines for Americans* in 2008. These were the first PA guidelines issued by the Federal Government.<br>- The fifth turning point relates to the development of the *National Physical Activity Plan* in 2010, with the core objective to change the social environment (e.g. communities,

	schools, and media).
Gomez, 2015 [108]	- The *Federal Obesity Prevention Act*, issued in 2008, required responsible agencies to evaluate how their budgets impacted nutrition, PA, and obesity in the population.<br>- The *Let's Move!* Campaign, started in 2010 by the First Lady Obama, mentions increase in population PA as one of its 4 goals. The programme was continued in 2012 under the name *Let's Move! Cities, Towns, and Counties initiative*.<br>- Some of the obesity prevention initiatives enacted by the Congress in the period from 2007 to 2009 include *the National Physical Education and Sports Week*, and *National Youth Sports Week*.
Katzmarzyk et al., 2016 [133] [134]	- The indicator *Government Strategies and Investment* from *RC on PA for Children and Youth* did not receive a grade. It was marked as incomplete, because of insufficient data were available for robust benchmarking.<br>- There are no guidelines related to SB.<br>- The national initiative *Let's Move! Active Schools* provides schools with tools, funding opportunities, resources, technical assistance, and professional development to create an active school environment where PA is integrated during, after, and before school, for at least 60 minutes per day.
Bornstein et al., 2014 [78]	- The *National Physical Activity Plan for the United States* was issued in 2010. It was developed through public-private partnerships. It involved government agencies, but it was not driven by the federal government, because its creators did not want to tie it to any political administration.
Bornstein & Pate, 2014 [77]	- The *Physical Activity Guidelines for Americans* were released in 2008.<br>- As the Department of Health and Human Services was completing the guidelines, representatives from the Centers for Disease Control and Prevention and PA and public health researchers and practitioners reached an agreement on the necessity of a national PA plan for the USA.<br>- The *National Physical Activity Plan for the United States* includes 52 evidence-based strategies and 215 tactics for increasing population PA levels.
Dentro et al., 2014 [98] [99]	- The indicator *Government Strategies and Investment* from *RC on PA for Children and Youth* did not receive a grade. It was marked as incomplete, because there were no benchmarks or data to inform the assessment of the indicator.<br>- The National Physical Activity Plan Alliance is a comprehensive set of initiatives, policies, and programmes aiming to increase PA in the general population.<br>- Besides PA guidelines, important PA promotion initiatives include: *President's Council on Fitness, Sports, and Nutrition;* the *Federal Safe Routes to School Program*; the *Community Transformation Grant Program*; *Let's Move!*; and *NHANES National Youth Fitness Survey.*
Xu et al., 2014 [220]<br>(in Chinese language)	- The first *Heathy People:*[4]*The Surgeon General's Report on Health Promotion and Disease Prevention* strategy, issued in 1979, mentioned negative health effects of prolonged sitting.<br>- The second strategy *Heathy People 1990: Promoting Health/Preventing Disease: Objectives for the Nation* introduced PA as a component of a healthy lifestyle.<br>- The third strategy *Heathy People 2000: National Health Promotion and Disease Prevention Objectives* mentioned PA as one of the key priority areas.<br>- The fourth strategy, *Heathy People 2010*, and the fifth strategy, *Heathy People 2020:* mentioned PA as a leading health indicator and specified detailed objectives regarding population PA.<br>- This initiative has a high level of sustainability because it is constantly improving, its objectives are measurable, and it has a good and interactive on-line platform. It is a result of a joint effort of different stakeholders and PA is perceived as one of the main indicators for health benefits.
Bellew et al., 2011 [8]	- The *Coordinated Approach to Childhood Health* school programme was established to promote healthy food choices and PA in school children.
Brown et al., 2011 [43]	- The use of *Physical Activity Guidelines for Americans*, issued by the US Department of Health and Human Services in 2008, is strongly recommended according to the Appraisal of Guidelines for Research and Evaluation quality grading (AGREE tool).

[4] A wrong translation of the programme name (*Healthy Citizen* instead of *Healthy People*) was used in the paper.

Eyler, 2011 [103]	- The *Healthy People 2020* strategy includes two goals related to PE: increase the number of students who participate in PE daily; and the increase number of schools that require daily PE.<br>- The strategy also includes a developmental goal to "increase legislative policies for the built environment that aim to enhance access to and availability of physical activity opportunities".<br>- The importance of active transport has also been emphasised in the strategy. The stated goals are to increase the number of adolescents and children (5-15 y.o.) that walk or cycle to school. These policies were financially supported by the *National Safe Routes to School Program*, Federal Highway Administration.
Oja & Titze, 2011 [234]	- In the document *Physical Activity Guidelines for Americans*, issued by the Department of Health and Human Services in 2008, there are specific recommendations for the following population groups: adults with disabilities; children and adolescents (6-17 y.o.); adults (18-64 y.o.); older adults (more than 65 y.o); and women during and after the pregnancy.
Pate et al., 2011 [37]	- Policy related to PE in schools states that: children should engage in at least two hours of PE a week; PE should be taught by highly qualified and certified teachers; and PE curriculum should be included in the school review process.<br>- PA policy in the area related to health education states that: cooperation with universities should be established to provide teacher education classes for inclusion of the *PA and Health* topic into the curriculum; training in motivational interviewing techniques related to PA should be provided to healthcare professionals in schools; the promotion of PA for achieving health benefits should be incorporated in health education classes; and education should be provided to parents to encourage them to regularly engage in PA and to be positive role models for their children.<br>- The data were extracted from the document *Physical Activity and Health: A Report of the Surgeon General,* published by the Department of Health and Human Services in 1996.
Koh, 2010 [136]<br>(in Korean language)	- The *Physical Activity Guidelines for Americans*, issued by the Department of Health and Human Services in 2008, include recommendations on PA and mention that inactivity should be avoided and that some PA is better than none.
Alderman et al., 2007 [65]	- In the Government report *Exercise and Health*, issued in 1915, it was mentioned that Americans were too sedentary and the Government encouraged citizens to be more active, that is, at least 20 minutes per day.<br>- In 1980s, the Government started to promote cycling and walking because it became obvious that engaging daily in exercise is unrealistic for many people.<br>- Important acts that address obesity and mention PA that have not yet become laws are:<br>a) The *Lifestyles and Prevention America Act* (HeLP America Act) – "A bill to improve the health of Americans and reduce health care costs by reorienting the Nation's health care system<br>toward prevention, wellness, and self-care"; and<br>b) The *Improved Nutrition and Physical Activity Act* (IMPACT Act) – "A bill to establish grants to<br>provide health services for improved nutrition, increased physical activity, obesity and eating disorder prevention, and for other purpose".
Uruguay	
Ramirez Varela et al., 2016 [32]	- The PA plan entitled *Plan Nacional de Actividad Fisica para la Salud* is under development.
Lachat et al., 2013 [140]	- In 2011, the Ministry of Health issued the national programme on nutrition entitled *Programa nacional prioritario de nutricion 2005–2009*.<br>- The programme includes the following targets and actions for PA promotion: achieve the recommended average of one hour of moderate PA per day for children and adolescents and 30 minutes of moderate PA a day for adults; and develop population-level guidelines for "PA and lifestyle".
Uzbekistan	
World Health Organization, 2010 [229]	- Policy documents in the PA area are not yet available.
Kahlmeier et al., 2015 [127]	- National PA recommendations have not yet been developed.
Vanuatu	

Kobayashi et al, 2017 [135]	- The document *National Physical Activity Development General Policy Directives 2007–2011: Key Policy Areas and 5 Years National Physical Activity Development Targets,* issued in 2007 by the Ministry of Youth Development and Training defined four key policy objectives and areas: carry out mapping and assessment of sports development resources; develop, facilitate, and review the implementation of specific policies related to protecting and promoting the "'right' of equal access and participation in physical activities, healthy and safe environment"; undergo resourcing, capacity development, and restructuring of the Ministry; and continue to develop and build partnership and cooperation with the international and national sports partners. The document promotes the development of "PA for all" as a key tool for the social and economic development in the country, through facilitation of programmes that promote employability, social cohesion, good health, and teamwork.<br>- The implementation of sports policy is mainly the responsibility of The Ministry of Youth Development and Training and the Vanuatu Sports Association and National Olympic Committee (VASANOC).<br>- It is obligatory for secondary and primary school students to take PE lessons.<br>- Sport policy has the three major components: social development through sport; development of sport to encourage youth to regularly participate in PA; and elite sports development. Despite sport being relatively low on the Government's priority list compared to other public policy areas, there is a growing interest in the first component of the sport policy.
Ramirez Varela et al., 2016 [32]	- NCD plan includes PA (details are not specified).
Venezuela, RB	
Herrera-Cuenca et al. 2016 [122] [123]	- The assigned grade for the indicator *National Level Policies* from *RC on PA for Children and Youth* is D.<br>- The national-level policies for PA promotion are: the *Constitution of the Bolivarian Republic of Venezuela* (which mentions the right to perform PA in article 111 and emphasises role of state in providing resources for promotion of recreation and sport); *The Organic Law of Sports, Physical Activity and Physical Education*, and the *National Fund for the Development of Sport, Physical Activity and Education Physics.*<br>- The national policy lacks "specific and articulated actions".<br>- An outdoor gyms proposal has been adopted by the Ministry of Popular Power for Youth and implemented by the National Sports Institute.
Ramirez Varela et al., 2016 [32]	- The PA plan entitled *Plan Nacional de Deporte, actividad fisica y Educacion Fisica 2013-2025* is available.
Vietnam	
Ramirez Varela et al., 2016 [32]	- NCD plan includes PA (details are not specified).
Lachat et al., 2013 [140]	- In 2012, the Vietnamese Prime Minister issued the *National nutrition strategy for 2011–2020, with a vision toward 2030*.<br>- The national policy includes target/action for PA promotion related to the development of physical exercise programmes at all educational levels, from the preschool to the undergraduate level).
Virgin Islands (U.S.)	
Ramirez Varela et al., 2016 [32]	- No national/subnational PA plan.
Wales	
Coenen et al., 2017 [38]	- In the publication *Start active, stay active - A report on physical activity for health from the four home countries' chief medical officers*, issued by the Government's Department of Health in 2011, it is stated that "all adults should minimise the amount of time spent being sedentary (sitting) for extended periods".
Ramirez Varela et al., 2016 [32]	- The PA plan entitled *Creating an Active Wales* is available.
Tyler et al., 2016 [207]<br>Stratton et al, 2016 [198]	- The assigned grade for the indicator *National Government Policy, Strategies and Investments* from *RC on PA for Children and Youth* is B-.<br>- Key documents that mention PA include: *Every Child Hooked on Sport*; *The Right to Play*; *Healthy Schools*; *Future Generations*; *Together for Health* (a 5-year strategy); and *Our Healthy Future.*<br>- The PA policies have not resulted in increased PA levels in the last ten years.

	- Reducing sedentary time is not mentioned in the documents.
Kahlmeier et al., 2015 [127]	- The national PA recommendations were published in the document entitled *Physical Activity, Health Improvement and Protection. Start Active. Stay Active: A report on physical activity from the four home countries',* issued in 2011.<br>- Specific recommendations are provided for children/young people, adults, and older adults.<br>- The PA recommendations for children/young people and adults are fully aligned with the WHO recommendations.<br>- The document includes recommendations on muscle-strengthening activities for adults and older adults and recommendations on SB for children/young people, adults, and older adults.
Christiansen et al., 2014 [44]<br>World Health Organization, 2011 [231]	- The following two documents related to HEPA and sport promotion were published in 2005: *Framework for the development of sport and physical activity* (action plan issued by the Sports Council Wales) and *Climbing higher – Welsh Assembly strategy for sport and physical activity.*<br>In this study, the two policy documents were referred to as a single strategy<br>- The strategy mentioned that people who are physically active are essential for the health of the nation. It is one of the rare strategies that have specific, measureable, achievable, relevant, and time-bound targets. Some of the targets include: all primary school children will participate in PA and sport for at least 60 min, five times a week; at least 90% of secondary school children will participate in PA and sport for at least 60 minutes, five times a week; in the next twenty years, Wales will match the best global standards for PA and sport levels.
Stratton et al., 2014 [199]	- The assigned grade for the indicator *National Policy, Strategy, and Investment* from *RC on PA for Children and Youth* is B.<br>- Some of the key documents that include PA are: *Climbing Higher*; *Creating an Active Wales*; *Sport Wales*; *Play Wales*; *Active Travel Wales Act*; *Turning Heads*; *Sustrans*; *and Food and Fitness.*
Brown et al., 2011 [43]	- The national policy document *Climbing Higher – The Welsh Assembly Government Strategy for Sport and Physical Activity* was published by the Welsh Assembly Government in 2005.
World Health Organization, 2010 [12]	- The following national documents related to PA were identified: *Healthy Ageing Action Plan for Wales* (2005); C*limbing Higher, the Welsh Assembly Strategy for Sport and Physical Activity* (2005); *Framework for the development of sport and physical activity* (2005); *Walking and cycling strategy for Wales* (2003); and *Welsh Assembly Government's Play Policy* (2002).
Musingarimi, 2009 [158]<br>Musingarimi, 2008 [157]	- The national PA-related documents are: *Food and Fitness – Promoting Healthy Eating and Physical Activity for Children and Young People in Wales: 5 Year Implementation Plan* and *Climbing Higher: The Welsh Assembly Government Strategy for Sport and Physical Activity* (2005). The latter document sets a strategy for PA and sport promotion for the next twenty years.
West Bank and Gaza	
Ramirez Varela et al., 2016 [32]	- No national/subnational PA plan.
World Health Organization, 2014 [232]	- There is a national NCD strategic plan or a policy that includes goals focused on PA (details not specified).<br>- There is an NCD committee that also coordinates PA promotion.<br>- There is legislation that mentions the requirements for the PE curriculum across different school grades.<br>- National policy documents cover the general population, children, and young people.<br>- Settings covered by national policy documents are: primary schools; high schools; primary health care; and sport and leisure.
Zimbabwe	
Manyanga et al., 2016 [150] [151]	- The assigned grade for the indicator *Government Strategies and Investments* from *RC on PA for Children and Youth* is D.<br>- The Ministry of Sports and Recreation has drafted *National Sports and Recreation Policy* (unpublished report from 2015).<br>- The *Sports and Recreation Commission Act* mentioned several PA-related goals, such as: monitor training programmes for sports persons; ensure that all people have

	opportunities to engage in recreation and sport; and develop recreational facilities.<br>- The Sports and Recreation Commission was established in 1991. It has set priorities for the organised sport promotion.<br>- More government commitment, evaluation, monitoring, and resource allocation for policies and strategies are needed.

AHK = Active Healthy Kids; EU = European Union; HEPA = health-enhancing physical activity; HEPA PAT = Health-enhancing physical activity policy audit tool; LTPA = Leisure-time physical activity; MET = metabolic equivalent; MVPA = moderate-to-vigorous physical activity"; NCD = Noncommunicable disease; NGO = Nongovernmental organization; PA = physical activity; PE = physical education; RC = report card; SB = sedentary behaviour; USA = United States of America; WHO = World Health Organization

Full text of the article is available in English, if not noted otherwise.
