# Supplementary material for: A global systematic scoping review of studies analysing indicators, development, and content of national-level physical activity and sedentary behaviour policies
Source: Int J Behav Nutr Phys Act. 2018 Nov 28;15:123. doi: 10.1186/s12966-018-0742-9 (PMC6263060; doi:10.1186/s12966-018-0742-9)
Supplement: Supplementary file 4 — Summary results of studies analysing indicators, development, and content of national-level physical activity and sedentary behaviour policies: international findings. (PDF 138 kb) [file 12966_2018_742_MOESM4_ESM.pdf]

Additional file 4 – Summary results of studies analysing indicators, development, and content of national-level physical activity and sedentary behaviour policies: international findings

Study	Countries included	Summary findings
Branca et al., 2007 [80]	19 European countries, including: Bulgaria, Croatia, Denmark, Estonia, Finland, France, Georgia, Hungary, Ireland, Latvia, Lithuania, the Netherlands, Norway, Portugal, Slovenia, Spain, Sweden, Turkey, and the United Kingdom	- Only a few countries had specific goals for PA or obesity, but most of the countries had nutrition-related goals.<br>- Most countries are addressing the obesity problem through various strategies.<br>- Obesity prevention strategies share the common goal of changing the school environment by providing a good platform for PA participation and improving health education.<br>- Eight documents propose building safe cycling and walking paths, reducing car use, and efficient public transport.<br>- Many countries included universities, research institutes, and associations of health professionals in the policy development.
Bellew et al., 2008 [6]	Australia Brazil, Canada, Finland, the Netherlands, New Zealand, Scotland, and Switzerland	Regarding PA policy development:<br>- all countries undertook broad consultation processes with key stakeholders from various sectors;<br>- all countries attempted to integrate PA policy with other national policies;<br>- all countries incorporated multiple strategies; "particularly multiple individual-oriented components, and to a lesser extent, environmentally-focused interventions";<br>- all countries had some monitoring of PA through national-level surveys in place;<br>- a systematic approach for evaluation and monitoring of the PA policy implementation has not been established in any of the countries;<br>- some countries developed clear branding or identity of the initiative.
Ceccarelli et al., 2011 [90]	34 countries from the Organisation for Economic Cooperation and Development and the EU, including: Australia, Austria, Belgium, Canada, Cyprus, Denmark, Estonia, Finland, France, Germany, Hungary, Ireland, Italy, Iceland, Japan, Korea, Latvia, Lithuania, Luxembourg, Malta, Mexico, the Netherlands, New Zealand, Norway, Poland, Portugal, Romania, Slovakia, Slovenia, Spain, Turkey, and the United Kingdom[1]	- Obesity is considered a key public health issue in all the countries.<br>- To tackle the problem of obesity, specific population groups were targeted in all the countries. The emphasis was put on children.<br>- Policies to improve healthy nutrition and promote PA were adopted in most of the countries.<br>- The countries of the WHO European region have policies to combat obesity that are mainly focused on food and nutrition practices, whilst only a few policies are related to PA.<br>- Quantifiable aims related to PA were set by only a few countries.<br>- Thorough analyses of population PA are present in policies from only eight

[1] Some documents from Malta, Cyprus, Latvia, Germany, Romania, Lithuania, Slovakia, Turkey, and Poland may not have been included in the analysis, because they were not written in English.

		countries.
Christiansen et al., 2014 [44]<br>World Health Organization, 2011 [231]	15 countries, including: Belgium, Bulgaria, Czech Republic, Estonia, Finland, Hungary, Ireland, Latvia, Lithuania, Malta, the Netherlands, Poland, Slovakia, Slovenia, and the United Kingdom	All analysed strategies (*n* = 18) mentioned:<br>- health benefits of sport;<br>- infrastructure as one of the important factors for population PA;<br>- children, young people, and people with disabilities as target groups;<br>- that the ministry responsible for sport has the responsibility for the implementation;<br>- the importance of "local-level involvement with regard to different aspects" and local-level implementation;<br>- schools as an important setting for PA and sport promotion in children and young people;<br>- health enhancing physical activity (HEPA);<br>- overall objectives of participation in PA and sport.
Daugbjerg et al., 2009 [11]	14 European countries, including: Czech Republic, Denmark, Finland, Germany, Hungary, Iceland, Ireland, the Netherlands, Norway, Portugal, Spain, Sweden, Switzerland, the United Kingdom (England)	- One or more ministries were involved in the development of all the analysed documents.<br>The implementation plan was emphasised in all the analysed documents.<br>- All analysed documents were legally non-binding.<br>- All analysed documents were published after 2000.<br>- Plans for the implementation of the policies were described in most of the analysed documents.<br>- Most policy documents targeted the whole population<br>- Few analysed documents targeted specifically people with low level of PA and people with disabilities.<br>- Only six documents included quantified PA goals.<br>- Most documents specified timeframes for the policy implementation.<br>- Less than a half of the documents indicated budgets for the policy implementation.<br>- Most of the documents highlighted the importance of evaluation plans and surveillance systems for monitoring the policy implementation.<br>- Only a half of the policies included a requirement/intention for evaluation.
Hämäläinen et al., 2015 [116]<br>Aro et al., 2016 [69]	6 countries, including: Denmark, England, Finland, Italy, the Netherlands, and Romania	- The policy documents lacked the use of evidence from "citable research" (i.e. book chapters, journal articles, working papers, and reports produced by universities, research institutes, and other independent research units). In the policy documents evidence from the following sources was rarely used: research based on surveys; peer-reviewed research articles; and evaluation, monitoring, and implementation studies.<br>- Research evidence that was used in the policy documents "was identified in an ad hoc manner in the policymaking phase and consisted of epidemiological research, population studies or statistics, and case studies".<br>- In the policy documents, "implicit evidence" (e.g. common knowledge, facts, and practices) was primarily used.

		- Most of the analysed countries do not have “routine reporting mechanisms for policy decisions using research evidence during the policymaking process”.<br>- The use of evidence in policymaking seems to depend on how close are the contacts between policymakers and researches.<br>- Research evidence was more used when developing policy at the local level than at the national and regional levels.
Ramirez Varela et al., 2017 [176]	139 countries, including: American Samoa, Angola, Antigua and Barbuda, Argentina, Aruba, Australia, Austria, Bahrain, Bangladesh, Barbados, Belgium, Bermuda, Bhutan, Bolivia, Botswana, Brazil, Brunei Darussalam, Bulgaria, Cameroon, Canada, Cayman Islands, Chile, China, Colombia, Costa Rica, Croatia, Cuba, Czech Republic, Denmark, Dominica, Dominican Republic, Ecuador, Egypt, Arab Rep., England, Estonia, Faeroe Islands, Fiji, Finland, France, French Polynesia, Germany, Ghana Greece, Greenland, Grenada, Guam, Guatemala, Guyana, Haiti, Hong Kong, Hungary, Iceland, India, Indonesia, Iraq, Ireland, Israel, Italy, Japan, Jordan, Kazakhstan, Kenya, Kiribati, Korea, Rep., Lao PDR, Lithuania, Luxemburg, Macao SAR, China, Macedonia, FYR, Malaysia, Maldives, Malta, Marshall Islands, Mexico, Micronesia, Fed. Sts., Moldova, Mongolia, Mozambique, Myanmar, Nepal, the Netherlands, New Caledonia, New Zealand, Nicaragua, Nigeria, Northern Ireland, Northern Mariana Islands, Norway, Oman, Palau, Palestine/West Bank and Gaza, Papua New Guinea, Paraguay, Peru, Poland, Portugal, Puerto Rico, Qatar, Romania, Russian Federation, Rwanda, Samoa, Saudi Arabia, Scotland, Senegal, Seychelles, Singapore, Slovak Republic, Slovenia, Solomon Islands, South Africa, Spain, Sri Lanka, St. Kitts and Nevis, St. Martin (French part), St. Lucia, St. Vincent and the Grenadines, Swaziland, Sweden, Switzerland, Syrian Arab Republic, Tanzania, Thailand, Tonga, Trinidad and Tobago, Tunisia, Turkey, Tuvalu, Uganda, Ukraine, United Arab Emirates, the United States of America, Uruguay, Vanuatu, Venezuela, RB, Vietnam, Virgin Islands (U.S.), and Wales	- Almost a half of the 139 analysed countries had plans related to NCDs that included PA.<br>- Standalone PA plans were found in 37 countries.<br>- No country in Middle East and North Africa, South Asia, and Sub-Saharan Africa had a standalone PA plan.<br>- Europe and Central Asia have the highest proportion of standalone PA plans.<br>- Sub-Sharan Africa is the region with the highest proportion of countries without PA plans.

Tremblay et al., 2016 [34]	38 countries, including: Australia, Belgium, Brazil, Canada, Chile, China, Colombia, Denmark, England, Estonia, Finland, Ghana, Hong Kong, India, Ireland, Japan, Kenya, Malaysia, Mexico, Mozambique, the Netherlands, New Zealand, Nigeria, Northern Ireland, Poland, Portugal, Scotland, Slovenia, South Africa, South Korea, Spain, Sweden, Thailand, United Arab Emirates, the United States of America, Venezuela, Qatar, Wales, and Zimbabwe	- For *Government Strategies and Investments* indicator, out of all the countries Denmark had the highest grade ("A- "), followed by Slovenia and the United Arab Emirates (both with grade "B+").<br>- Twelve countries reported grades in the "B" range.<br>- Six countries did not assign grades for this indicator.<br>- Most countries reported good governmental PA policies and strategies, but several indicated the lack of implementation, evaluation, and quality assurance.
Tremblay et al., 2014 [33]	15 countries, including: Australia, Canada, Colombia, England, Finland, Ghana, Ireland, Kenya, Mexico, Mozambique, New Zealand, Nigeria, Northern Ireland, Scotland, South Africa, and the United States of America	- Five countries did not assign grades for the indicator *Government Strategies and Investments.*<br>- Grades were within "B" and "C" ranges, regardless of the country's income level.<br>- Many countries had high grades for policy environments in governments and schools and low grades for health behaviour indicators policies were targeting.<br>- More developed policy environments were found in countries with higher economic standard.
World Health Organization, 2010 [228]	27 countries, including: Austria, Belgium, Bulgaria, Cyprus, Czech Republic, Denmark, Estonia, Finland, France, Germany, Greece, Hungary, Ireland, Italy, Latvia, Lithuania, Luxembourg, Malta, the Netherlands, Poland, Portugal, Romania, Slovakia, Slovenia, Spain, Sweden, and the United Kingdom	- A considerable increase in the number of policy documents related to PA promotion was noticed.<br>- Most of the identified policies were national-level documents.<br>- Policy documents within the public health sector were found in twenty-one countries.<br>- Documents in the transport sector were found in 11 countries.<br>- Three countries had documents in the environment sector.<br>- Only one country had a document related to road infrastructure and safety.<br>- The documents (n = 129) focused on the following key sectors or population groups: nutrition (n = 38); public health (n = 28); obesity (n = 14); PA and nutrition (n = 12); children (n = 11); NCDs (n = 9); education (n = 6); agriculture (n = 6); consumers (n = 2); healthy ageing (n = 2); and inequalities (n = 1).<br>- Time frames in the documents varied from three to ten years.
World Health Organization, 2015 [233]	26 countries, including: Austria, Belgium, Bulgaria, Croatia, Cyprus, Czech Republic, Denmark, Estonia, Finland, France, Germany, Greece, Hungary, Ireland, Italy, Latvia, Lithuania, Luxembourg, Malta, the Netherlands, Poland,	- Twenty-two countries have national "Sport for all*"* policies.<br>- Twenty-seven countries have adopted policies in the sports sector, 22 in the health sector, and 19 in the education sector.<br>- More than 50% of countries (n = 16) developed national coordination leadership and mechanisms related to HEPA promotion.

	Portugal, Romania, Slovakia, Slovenia, Spain, Sweden, and the United Kingdom	- Twenty countries are members of international networks (mainly the WHO Healthy Cities Network).<br>- One third of policies is focused on elderly and socially disadvantaged groups.<br>- Women during and before pregnancy are the least represented target group in PA policies.

EU = European Union; HEPA = health-enhancing physical activity; NCD = noncommunicable disease; PA = physical activity; RC = report card; WHO = World Health Organization
