# Supplementary material for: A global systematic scoping review of studies analysing indicators, development, and content of national-level physical activity and sedentary behaviour policies
Source: Int J Behav Nutr Phys Act. 2018 Nov 28;15:123. doi: 10.1186/s12966-018-0742-9 (PMC6263060; doi:10.1186/s12966-018-0742-9)
Supplement: Supplementary file 5 — Definitions of policy in general, public policy, physical activity policy, health policy, and policy document included in studies analysing indicators, development, and content of national-level physical activity and sedentary behaviour policies. (PDF 114 kb) [file 12966_2018_742_MOESM5_ESM.pdf]

Additional file 5 – Definitions of policy in general, public policy, physical activity policy, health policy, and policy document included in studies analysing indicators, development, and content of national-level physical activity and sedentary behaviour policies

Publication	Definitions
Active Healthy Kids Canada, 2012 [54]	"Public policies have consequences for communities and people. Examples of public policies include government budgets and taxation rules. Public policies provide direction to the central branch of most modern governments – the executive (e.g., Prime Minister's Office, cabinet and the bureaucracy at the federal governmental level in Canada) – and find expression in government laws and regulations [238]".
Active Healthy Kids Canada, 2014 [56] Active Healthy Kids Canada, 2013 [55]	"'Policy can be defined as a legislative action, organized guidance or rule that may affect people's physical activity environment or behaviour' [239]. Policies can be in the form of written codes or standards that guide choices or common practices. Both government and non-government organizations have a role to play in shaping policies that aim to increase physical activity and decrease sedentary behavior in Canadian children and youth".
Al-Bahlani and Mabry, 2014 [62]	"The following definitions, based on Merriam Webster dictionary, were used to classify the documents: …policy—a method of action . . . to guide and determine present and future decisions…"
Bellew et al., 2008 [6]	"Physical activity policy has been defined as: 'a formal statement that defines physical activity as a priority area, states specific population targets and provides a specific plan or framework for action. Further, it is held that a policy should 'describe the procedures of institutions in the government, non government and private sector to promote physical activity in the population, and defines the accountabilities of the involved partners' [26]".
Bellew et al., 2011 [8]	"'Health policy' is a formal statement or procedure within institutions (notably government) that defines priorities and the parameters for action in response to health needs, available resources, and other political pressures [240]. The main aim of physical activity–related public policy is to create supportive environments, infrastructure, and pro- grams to enable people to lead active lives. It makes the social and physical environments health-enhancing [240]. Our use of the term 'public policy' in this article combines these two definitions proposed in the WHO health promotion glossary; we emphasize that the actions required to promote physical activity involve multiple agencies and sectors and not merely the health sector. Policy may be conceptualized at three levels reflecting social and political commitment [16]: (1) formal written codes, regulations, or decisions with legal authority (legislation and urban planning zoning are examples of this type of policy); (2) written standards that guide choices (guidelines suggesting physical education standards for all school-age children are an example of standards that guide but do not mandate policy); and (3) unwritten social norms that influence behavior (including the culture of sedentariness, reduced energy expenditure in everyday lives). 'Evidence-based public policy' is based on research that has undergone quality assurance and methodologic scrutiny".
Brown et al., 2011 [43]	"A policy is a formal statement that should define priorities for action, goals and strategies as well as accountability and allocation of resources [26]".
Bull et al., 2004 [26]	"Policy is a formal statement that defines priorities for action, goals and strategies, as well as accountabilities of involved actors and allocation of resources. Policy provides a guide to action to achieve intended goals, initiated by government, non-government or private sector organisations, and can occur on a written (eg, within legislation, policy documents) or on an unwritten basis (eg, within usual practice). Although sometimes confusing, policy can also refer to political or bureaucratic processes, as well as to particular decisions… …the following definition of a policy on PA was developed: a formal statement that defines physical activity as a priority area, states specific population targets and provides a specific plan or framework for action. Further, it is held that a policy should 'describe the

	procedures of institutions in the government, non government and private sector to promote physical activity in the population, and defines the accountabilities of the involved partners”.
Bull et al., 2004 [86]	“Policy describes a procedure to gain desired outcomes, initiated by government, non-government or private sector organisations. It is a formal statement that defines priorities for action, goals and strategies, as well as accountabilities of involved actors and allocation of resources. They provide a guide to action to achieve the intended goals. Policy can occur on a written (e.g. within legislation, policy documents) or on an unwritten basis (e.g. within usual practice), and it can refer to particular decisions as well as to political and bureaucratic processes… Physical activity policy is a formal statement that defines physical activity as a priority area, states specific targets and provides a specific plan or framework for action. It describes the procedures of institutions in the government, non-government and private sectors to promote physical activity in the population. In addition it should define the accountability of the involved partners”.
Chimeddamba et al., 2015 [92]	“Health policy is defined as the decisions, plans, and actions undertaken to achieve specific health care goals within a society [241]”.
Christiansen et al., 2014 [44]	“A policy is defined as a written document, which has been endorsed, including statements and decisions defining goals, priorities and main directions for attaining these goals”.
Craig, 2011 [95]	“…policy is defined as written documents or statements that identify PA as a priority area for the population or target populations and outlines a framework for action”.
Daugbjerg et al., 2009 [11]	“…policy documents are written documents that contain strategies and priorities, define goals and objectives, and are issued by a part of the public administration”.
Eyler, 2011 [103]	“Physical activity policy is a legislative action, organized guidance, or rule that may affect the physical activity environment or lifestyle behavior. These policies can be in the form of formal written codes, written standards that guide choices, or common practices [16].”
Guo and Pan, 2016 [113] (in Chinese language)	“PA policy is a formal written document that provides guidelines for PA promotion”. –translation from Chinese.
Kalman et al., 2008 [129]	“Policy is understood as the process and method of binding decision making of a certain group of people with pluralistic interests and opinions. In terms of these collective decisions, policy constitutes the art of administering public affairs, the art of governing a country and protecting the interests of one country against another one, creating and maintaining relationships”.
Lachat et al., 2013 [140]	“…a broad definition of policy was used, and all national documents that included the national objectives and guidelines for action in the domain of diet and/or physical activity and/or prevention of NCDs were included”.
Mendez, 2015 [153] (in Spanish language)	“The definition of public policy can be constructed from two basic aspects: a) policies as the set of interrelated decisions about the selection of goals and the means to achieve them, starting from the exercise of public authority [243] and b) the public, which for Nora Rabotnikof [242], is observed in three senses: the public as interest or common utility, as opposed to private; the public as the manifest and ostensible, a contrary to the secret and preserved; and the public as accessible for all, a contrary to the closed”. - translation from Spanish.
Milton and Bauman, 2015 [40]	“…four key aspects of physical activity policy: 1) national recommendations on physical activity levels; 2) national goals and targets; 3) surveillance or health monitoring systems; and 4) public education”.
Pate et al., 2011 [37]	“We defined physical activity policy as ‘a formal written document that provides guidelines to promote physical activity in the public’”.
Piggin, 2008 [168]	“A working definition here however is borrowed from Jenkins (1978) who describes public policy as ‘a set of interrelated decisions taken by a political actor concerning the selection of goals and the means of achieving them

	within a specified situation where these decisions should, in principle, be within the power of these actors to achieve' [244]."
Pratt et al., 2016 [171]	"'Policy provides an organizing structure and guidance for collective and individual behavior. It may be defined as legislative or regulatory action taken by federal, state, city, or local governments, government agencies, or nongovernmental organizations such as schools or corporations. Policy includes formal and informal rules and design standards that may be explicit or implicit'[16]".
Rütten et al., 2013 [185]	"Physical activity promotion policies are complex and multidisciplinary policy approaches that depend on multiple actors at various levels of policy-making…"<br>Definition outlined in connected study: "The WHO defines health policy as a "formal statement or procedure within institutions (notably government) which defines priorities and the parameters for action in response to health needs, available resources and other political pressures" [240]. Our approach, by contrast, is based on a broader definition of policy, which also includes informal institutional arrangements and procedures as well as rationales for action on health- related issues". [245]
Schöppe et al., 2004 [187]	"Policy describes a procedure or a guide to action to achieve intended goals, initiated by governmental, non governmental or private sector organisations. It determines the means by which the environment is to be altered to gain desired outcomes. At best, because this makes policy more obvious to the public, it is based on a formal statement that defines priorities for action, goals and strategies, as well as accountabilities of involved actors and allocation of resources. Policy can occur on a written (e.g. within legislation, policy documents) or on an unwritten basis (e.g. within usual practice), and it can refer to particular decisions as well as to political and bureaucratic processes. A main characteristic of policy is the procedure aspect, in the literature described as policy process or policy cycle involving the phases initiation, adoption, implementation, evaluation, reformulation... Policy is often not a single decision, but a web of decisions or sometimes rather a non-decision… 'Physical activity policy is a formal statement that defines physical activity as a priority area, states specific population targets and provides a specific plan or framework for action. It describes the procedures of institutions in the government, non government and private sector to promote physical activity in the population, and defines the accountabilities of the involved partners.'"
Seppälä et al., 2017 [39]	"Health policy is defined by the World Health Organization as 'decisions, plans, and actions that are undertaken to achieve specific health care goals within a society' [241]. Policies are a means for generating and/or supporting the implementation of health behaviour change interventions, which are a set of activities designed to bring about change; thus policies are crucial for the interventions' implementation and outcomes".
Woods and Mutrie, 2012 [218]	"A physical activity policy is an example of a public health policy. It is a document that defines physical activity as a priority area; that identifies specific population goals and targets, and that provides a framework for action, or an action plan to achieve these goals [6]. Ideally, a physical activity policy should also define of roles and responsibilities of involved partners, allocation of resources, and clearly identify accountability for implementation of specific components of the policy aligned to a realistic and achievable timeframe [26]".
World Health Organization, 2010 [12]	"…policy documents were defined as written documents that contain strategies and priorities, define goals and objectives, and were issued by part of the public administration".
World Health Organization, 2010 [228]	"The definition of 'policy' as used in the glossary of terms, i.e. a 'written document that contains strategies and priorities, define goals and objectives and is issued by a part of the administration', was highlighted…".
World Health Organization, 2010 [229]	"The following definition of 'policy' was used in identifying national policies: 'a written document that contains strategies and priorities, defines goals and objectives, and is issued by a part of the administration'".

World Health Organization, 2011 [231]	“A policy was defined as a written document, which has been endorsed, including statements and decisions defining goals, priorities and main directions for attaining these goals. It may also include an action plan on implementation”.

PA = physical activity

Full text of the articles available in English, if not noted otherwise.
